# One‐Step Radical‐Intensified Selective Etching (RISE) Strategy for High‐Yield Synthesis of Monolayer MXene with Tailored Nanoholes

**DOI:** 10.1002/anie.9523099

**Published:** 2026-05-04

**Authors:** Chenxu Liu, Hao Zhang, Anirban Sikdar, Kanglei Pang, Guangyuan Ma, Kai Xi, Shujiang Ding, Jiayin Yuan, Miao Zhang

**Affiliations:** ^1^ School of Chemistry Xi'an Jiaotong University Xi'an P. R. China; ^2^ Department of Chemistry Stockholm University Stockholm Sweden

**Keywords:** MXene, selective etching, high yield, defect engineering, capacitive deionization

## Abstract

MXenes have gathered immense scientific attention due to their unique combination of high electronic conductivity, hydrophilicity, and reduced dimensionality. While considerable advances in synthetic methodologies, achieving rapid, high‐yield production of dispersible monolayer MXenes with controllable in‐plane structure remains a daunting challenge. Herein, we report an ultrafast radical‐intensified selective etching (RISE) tactic that enables one‐step mild synthesis of monolayer Ti_3_C_2_T*_x_* MXene bearing customized in‐plane nanoholes with near‐quantitative etching efficiency (∼99.9%) within merely 3 h. By fine‐tuning the dosage of H_2_O_2_, which generates hydroxyl radicals (**·**OH) in situ, defect‐lean monolayer MXene was made in a high yield of 81.6%. Liters of such colloidal dispersion of monolayer MXene were obtained within hours, which could be readily processed into conductive films with improved oxidation resistance. Mechanistic studies reveal that the RISE protocol follows a radical‐driven redox pathway fundamentally distinct from traditional proton‐mediated etching routes. As a proof of concept, holey MXene‐derived conductive films demonstrated an exceptional desalination capacity of 32.71 mg g^−1^ in capacitive deionization, outperforming most pure MXene‐based electrode materials. Our method can potentially revolutionize the prevailing wet chemical etching protocol used for a decade for yielding monolayer MXene and establishes a swift pathway toward customizable MXene architectures for energy and environmental applications.

## Graphical abstract

Radical‐intensified selective etching (RISE) delivers monolayer MXenes with >80% yield in just 3 h etching process. Controlled **·**OH dosage enables one‐step access to holey MXenes with abundant nanopores or defect‐lean flakes. This mild, safe strategy couple ultrafast kinetics with structural tunability, advancing precision design of MXene architectures.

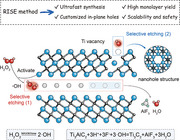

## 1 Introduction

Two‐dimensional (2D) colloidal nanosheets featuring atomic thickness, outstanding electronic conductivity, and accessibility to large‐scale production are of considerable scientific interest in cutting‐edge materials science research [1]. Among them, 2D transition metal carbides and nitrides known as MXenes, in a general formula of M_n+1_X_n_T*_x_*, where M represents a transition metal (Ti, Ta, Nb, V, Mo), X denotes C and/or N, and T refers to surface termination groups (–O, –OH, or –F), have emerged as a uniquely versatile family of nanomaterials. MXenes combine metallic conductivity, rich and tunable surface chemistry, and broad compositional diversity, and are promising candidates for applications ranging from energy storage to catalysis and water purification [2, 3]. Since the first discovery of Ti_3_C_2_T*_x_*, researchers worldwide have been actively pursuing reliable, high‐yield, and time‐efficient methods for synthesizing multi‐ and/or monolayer MXenes [4, 5, 6, 7, 8]. Except for a few recent reports on bottom‐up methods for multilayer MXenes, top‐down methods remain the predominant ones for MXene synthesis to date [9, 10, 11]. Among them, the solution‐based wet‐chemical etching protocol was the first established for selectively removing A‐layer from MAX phase precursors. Hydrofluoric acid (HF), as a potent etchant, offers high etching efficiency, fast reaction kinetics, and good yield. However, this approach typically results in multilayer “accordion‐like” structures due to strong interlayer interaction and uncontrollable defects derived from harsh etching conditions. Further delaminating them into monolayer necessitates inorganic/organic intercalation and delamination steps. To alleviate the environmental and safety concerns associated with HF, greener fluorine‐free alternatives, such as the electrochemical etching strategy and high‐temperature alkaline treatment have been proposed consecutively [12, 13, 14]. Nevertheless, these methods primarily yield multilayer MXenes with undesirable oxide/hydroxide byproducts or generate a very low monolayer yield, hindering further processing and hybridization with other functional materials. Following the wet‐chemical methods, a molten salt‐based etching tactic has been developed and significantly expanded the spectrum of accessible MXenes [15, 16, 17]. This versatile method, however, primarily yields multilayer MXene products bearing inevitable impurities even after post‐synthetic repeated washing. Despite efforts on the delamination of MXenes produced via molten salt etching, the yield of the monolayer remains unsatisfactory [18, 19, 20]. Moreover, dry selective extraction through gas‐solid reaction emerged as a promising alternative to rapidly etching MAX within a short timeframe [21]. However, these newly established methods in principle deliver multilayer powders at the current stage, and scalable monolayer synthesis via these routes remains elusive.

Albeit with the abovementioned advances and efforts in MXene synthesis, acidic fluorine‐based etching—particularly the LiF/HCl system—continues to dominate large‐scale monolayer MXene production due to its favorable balance of etching efficiency, safety, and in situ intercalation benefits [22]. Nevertheless, a large room is left for improving its monolayer yield. Accordingly, extensive effort has been dedicated to optimizing the post‐etching delamination step, employing strategies such as hydrothermal‐assisted intercalation, freeze‐thaw cycling, high‐temperature ultrasonication, and high‐pressure homogenization [23, 24, 25, 26]. These two‐step strategies, where etching and delamination are conducted separately, appear better to some extent in terms of the monolayer yield at the cost of increased time and energy consumption. Taken together, all the methodologies proposed to date face multiple time‐ and energy‐intensive issues and fail to achieve high‐yield monolayer MXene nanosheets in a short processing duration. Furthermore, most current efforts prioritize etching and exfoliation efficiency, with limited attention paid to tailoring the structural quality of the resulting flakes. Yet, this aspect is critical: Monolayers with controlled in‐plane defects or nanopores offer enhanced functionality for ion transport and catalysis, while high‐quality, minimally defective flakes are desirable for electronic stability and conductivity. Therefore, a synthetic strategy that concurrently targets high yield and structural precision would offer transformative benefits, unlocking broader applicability across energy and environmental systems.

Herein, we established a radical‐intensified selective etching (RISE) strategy that leverages hydroxyl radicals (**·**OH) generated from H_2_O_2_ within a LiF/HCl medium to simultaneously accelerate A‐layer removal and enable precise intralayer structural engineering. This method achieves an 81.6% monolayer yield in a 3 h etching step under mild conditions (40 °C), producing defect‐lean nanosheets with a lateral size of 4.1 ± 2.4 µm, without additional intercalation and delamination steps. More strikingly, by fine‐tuning the concentration of H_2_O_2_ in the reaction mixture, we were allowed to access in one step holey MXene nanosheets featuring abundant in‐plane nanopores at a quantitative etching efficiency and 68.0% monolayer yield under the same mild conditions. To the best of our knowledge, it is the first report on one‐step synthesis of holey MXene nanosheets, not to mention its high yield and short processing time. It contrasts with conventional monolayer synthesis strategies, which are designed to preserve lattice integrity. In this approach, by leveraging controlled radical oxidation, we intentionally induce in‐plane porosity during the etching process, thereby integrating synthetic control with structural precision. It allows for a high level of tunability in MXene design, where engineered nanopores can be harnessed to modulate properties such as ionic transport, surface accessibility, and potential catalytic activity. As a proof of concept, a film electrode fabricated from these holey MXene nanosheets exhibited an impressive desalination performance of 32.71 mg g^−1^ in capacitive deionization (CDI) that outperforms most single 2D materials‐based film electrodes. The method developed here will reshape the prevailing MXene synthesis paradigms and unlock a wide window of applications demanding monolayer holey MXene nanosheets.

## 2 Results and Discussion

**FIGURE 1:**
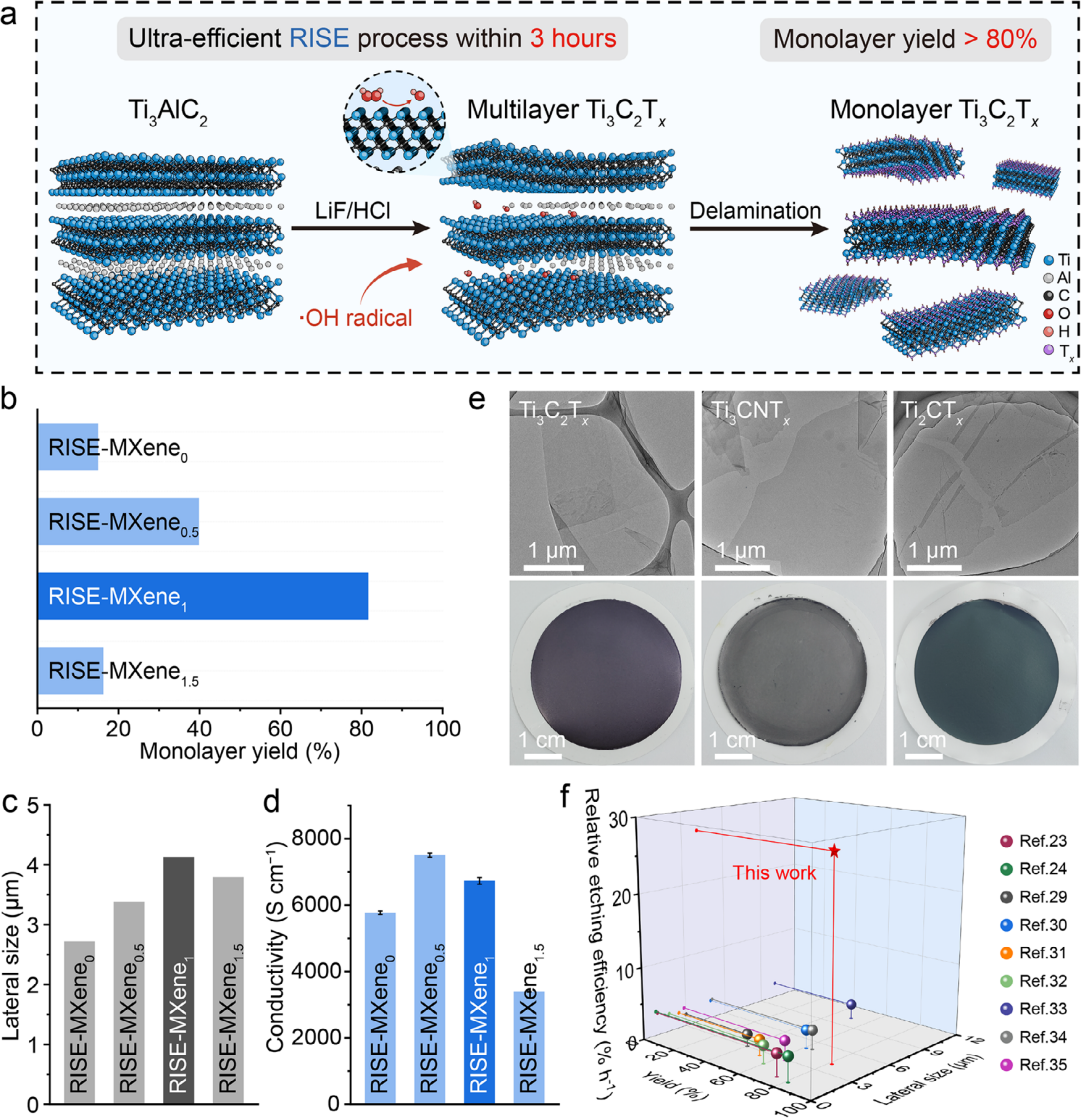
(a) Schematic illustration of the RISE process for producing monolayer MXene. (b) Monolayer yield of Ti_3_C_2_T*_x_* MXene as a function of H_2_O_2_ content. (c) Lateral size and (d) electrical conductivity of Ti_3_C_2_T*_x_* nanosheets produced under varying H_2_O_2_ concentrations. (e) Transmission electron microscopy (TEM) images of Ti_3_C_2_T*_x_*, Ti_3_CNT*_x_*, and Ti_2_CT*_x_* nanosheets and corresponding photographs of freestanding films. (f) Comparison analysis of the RISE method with previous studies.

In the conventional LiF/HCl etching protocol, known as the minimally intensive layer delamination (MILD) method, HF generated in situ acts as the primary etchant to extract A‐layer from the MAX phase. In this process, protons (H^+^) and water (H_2_O) act as moderate oxidants to support the oxidation of aluminum (Al) atoms and facilitate the etching reaction [27]. The overall reaction rate is governed by the etching kinetics and mass transport of reactants and byproducts. In general, this process lasts over 48 h at 35° C till the etching process completes. Attempts to accelerate the reaction by using elevated HF concentrations or higher temperatures often raise serious safety and handling concerns. In this context, we bypass the toxic, highly concentrated HF and unfavorable high‐temperature conditions, and leverage oxidation reaction‐driven enhancement to speed up this sluggish process [28]. As shown schematically in Figure 1a, our approach, termed RISE, incorporates H_2_O_2_, a green oxidant, into the LiF/HCl system. The in situ generated **·**OH speeds up the oxidation and extraction of Al layers while simultaneously expanding interlayer spacing, thus promoting both etching efficiency and delamination readiness. The product is named as RISE‐MXene_x_, where “x” denotes the volume of H_2_O_2_ added. In a typical run, 1 g of MAX precursor powder (Figures S1–S3) was immersed in an etching solution containing a controlled amount of 30 wt% H_2_O_2_ (1 mL); the reaction was conducted at 40 °C for 3 h. Notably, a critical step involved the stepwise addition of H_2_O_2_ in six equal portions to mitigate its rapid self‐decomposition and maintain a sufficient radical concentration throughout the etching process. Via etching away, the intermediate Al atom layers, the resulting multilayer MXenes were thoroughly washed with deionized water (DIW) till a pH value of six (typically consuming 1.5 L of DIW per gram of MAX). The final step involved the collection of monolayer MXene through centrifugation after sequential shaking and ultrasonication. Taking Ti_3_C_2_T*_x_* as a case study, the impact of H_2_O_2_ dosage on the monolayer yield was systematically investigated (Figure 1b). At 1 mL H_2_O_2_ per gram of MAX, the monolayer yield peaked at 81.6%, defined as the gravimetrically determined mass of the monolayer collected from the supernatant after delamination/centrifugation relative to the initial MAX mass. This markedly surpasses the 14.9% yield obtained under identical conditions without radical assistance (RISE‐MXene_0_). Moreover, the scalability of this protocol was tested in an upscaled reaction using 10 g of MAX precursor, which yielded a high monolayer yield of 70% (Figure S4). It should be mentioned that further improvement of the monolayer yield is attainable through optimization of the delamination process, such as extended ultrasonication time or increased energy input.

Next, we evaluated the effect of H_2_O_2_ concentration on the lateral size and conductivity of the prepared MXene nanosheets (Figures 1c, d, and S5–S6). A moderate amount of H_2_O_2_ (1 mL per batch) was found to promote sufficient selective etching of the intermediate Al layers, resulting in improved flake quality with an average lateral size of 4.1 ± 2.4 µm and a high conductivity of 6729.9 ± 96.6 S cm^−1^. Of note, raising the H_2_O_2_ dosage to 1.5 mL suppressed the lateral size to 3.8 ± 1.9 µm, accompanied by a slight drop in conductivity to 3390.2 ± 13.3 S cm^−1^. Understandably, as a green oxidant, H_2_O_2_ can attack the Ti atom accompanied by the reaction with Al atom. Insufficient H_2_O_2_ results in incomplete A‐layer removal, leaving partially etched multilayer particles that are difficult to delaminate into large monolayers. In contrast, excessive H_2_O_2_ accelerates etching, but can also induce over‐etching/oxidative attack on the MXene framework, disrupting the layer integrity and reducing lateral size. These results underscore the importance of optimizing the oxidant dosage to reach a balance between etching efficiency and material quality. Impressively, the one‐step RISE protocol demonstrated here is versatile and broadly applicable to the synthesis of a broad spectrum of MXene materials, including Ti_3_CNT*_x_* and Ti_2_CT*_x_*, as depicted in Figure 1e. Scanning electron microscopy (SEM) images of both Ti_3_CNT*_x_* and Ti_2_CT*_x_* (Figures S7, S8) reveal similar lamellar morphology, in support of the generalizability of this strategy across diverse MAX precursors. Figure 1f and Table S1 summarize the comparative analysis of the relative etching efficiency (defined as the ratio of monolayer yield to etching time), yield, and lateral size against other reported synthetic methods. Of note, the RISE strategy outperforms the state‐of‐the‐art methods in terms of both reduced etching time and enhanced monolayer yield, positioning itself as one of the best choices for large‐scale, high‐quality MXene production [23, 24, 29, 30, 31, 32, 33, 34, 35].

**FIGURE 2:**
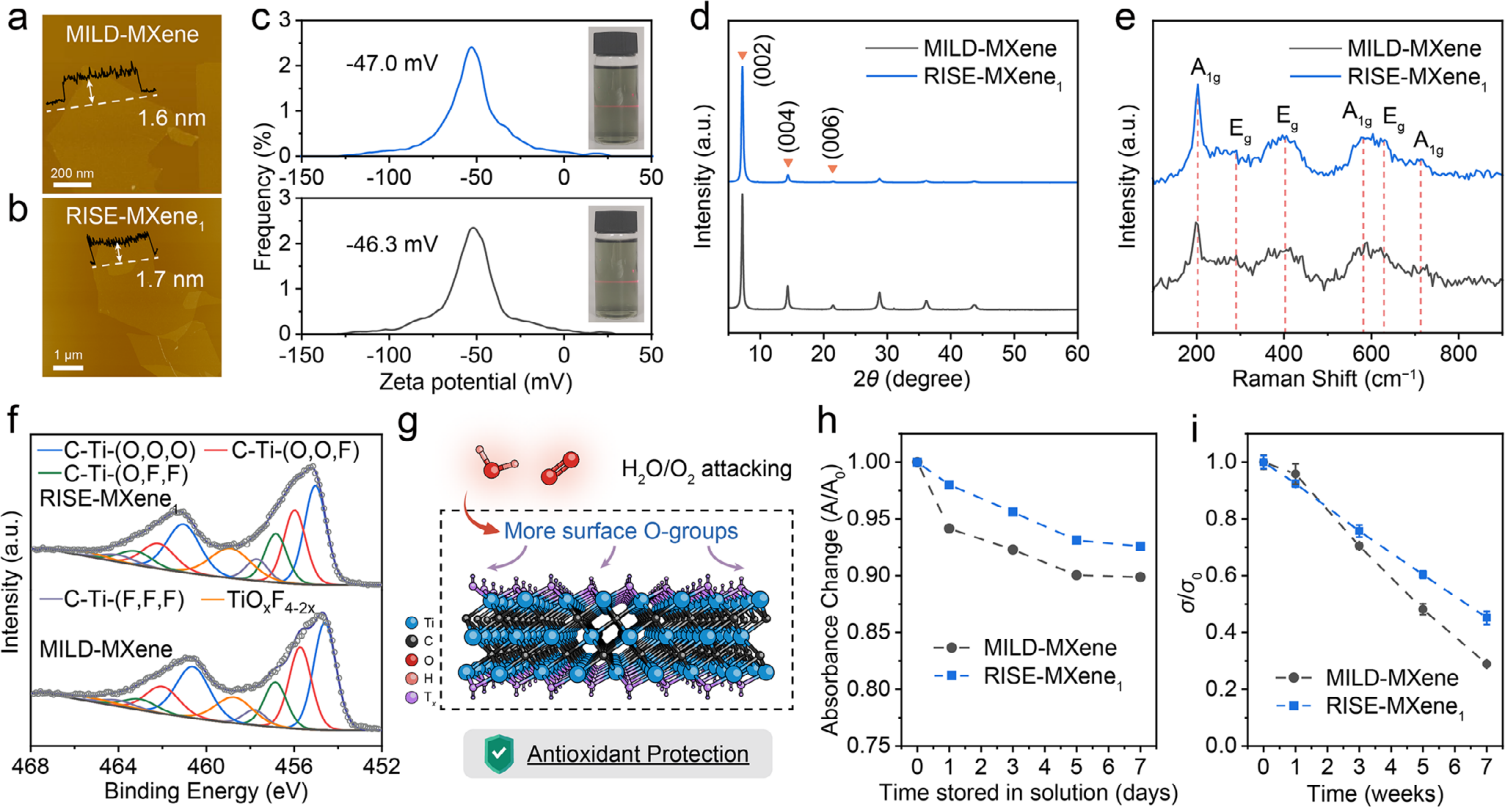
AFM images of (a) MILD‐MXene and (b) RISE‐MXene_1_, where insets correspond to the height profiles. (c) Zeta potential and Tyndall effect of MILD‐MXene and RISE‐MXene_1_. (d) XRD patterns of MILD‐MXene and RISE‐MXene_1_ films. (e) Raman spectra of MILD‐MXene and RISE‐MXene_1_ films. (f) High‐resolution XPS spectra of MILD‐MXene and RISE‐MXene_1_ with Ti 2p. (g) Schematic illustration of the RISE‐MXene_1_ surface chemistry. (h) Absorbance changes in the UV–vis spectra over one week for RISE‐MXene_1_ and MILD‐MXene aqueous dispersion at 327 nm. (i) Electrical conductivity changes for RISE‐MXene_1_ and MILD‐MXene films under ambient conditions.

To probe the potential impact of H_2_O_2_ on the product, RISE‐MXene_1_, which has the highest monolayer yield, was comprehensively characterized and compared with the Ti_3_C_2_T*_x_* MXene prepared by the conventional MILD method (hereafter referred to as MILD‐MXene). As determined by atomic force microscopy (AFM) in Figure 2a, b, both MILD‐MXene and RISE‐MXene_1_ nanosheets exhibited a clean surface with similar thicknesses of 1.6 and 1.7 nm, respectively. The smooth and lamellar morphology of the RISE‐MXene_1_ nanosheets was reconfirmed by high‐angle annular dark‐field scanning transmission electron microscopy (HAADF‐STEM) characterization (Figure S9a). High‐resolution TEM (HR‐TEM) and corresponding fast Fourier transform (FFT) images confirmed the hexagonal lattice symmetry and well‐preserved crystallinity of RISE‐MXene_1_, indicating that the RISE process does not compromise the lattice order (Figure S9b). Besides, energy‐dispersive x‐ray spectroscopy (EDS) elemental mapping identified the uniform distribution of Ti, F, C, and O elements (Figure S9c). These results demonstrate a high degree of consistency between RISE‐MXene_1_ and MILD‐MXene on the emblematic monolayer nanosheet structure. Figure 2c illustrates the Tyndall effect in both RISE‐MXene_1_ and MILD‐MXene dispersions, indicative of their colloidal nature. The Zeta potential of RISE‐MXene_1_ was measured as −47.0 mV, closely matching −46.3 mV of MILD‐MXene, explaining well its stable dispersion behavior. Similarly, the colloidal dispersions of Ti_3_CNT*_x_* and Ti_2_CT*_x_* also exhibited the Tyndall effect, with Zeta potential of −38.2 and −49.7 mV, respectively (Figure S10), manifesting the RISE strategy yields stable and processable dispersions across multiple MXene chemistries.

To further investigate the subtle structural variance and macro‐assembly characteristics granted by these new protocols, both RISE‐MXene_x_ and MILD‐MXene dispersions were processed into freestanding films via vacuum‐assisted filtration deposition (Figure S11). Surface SEM images of all MXene films presented varying degrees of wrinkles, while the cross‐section images displayed similar laminar architectures. Notably, the RISE‐MXene_1_ film showed a flatter surface, which is attributed to the larger lateral size. Figure 2d shows the x‐ray diffraction (XRD) patterns of RISE‐MXene_1_ and MILD‐MXene films. The characteristic peaks of RISE‐MXene_1_ resemble those of MILD‐MXene. The prominent (002) diffraction peak in both samples shifted from 9.5° (in the MAX phase) to 7.2°, which confirms the removal of Al layers from MAX (Figure S12). The Raman spectra of RISE‐MXene_1_ aligned with that of MILD‐MXene, with peaks at 202, 290, 405, 590, and 718 cm^−1^, which are ascribed to the vibrational modes of *A*_1g_ (Ti, O, C), *E*_g_ (T*_x_* = OH), *E*_g_ (T*_x_* = O), *E*_g_ (T*_x_* = OH), and *A*_1g_ (C), respectively (Figures 2e and S13) [36]. Upon increasing the H_2_O_2_ content to 1.5 mL within the etching system, the Raman peak of anatase TiO_2_ at 151 cm^−1^ emerged, signifying the oxidation effect of excessive H_2_O_2_ on the MXene structure.

The chemical states of the RISE‐MXene_x_ and MILD‐MXene were investigated by x‐ray photoelectron spectroscopy (XPS) as shown in Figures 2f and S14–S18. The survey spectra revealed four main peaks corresponding to C 1s, Ti 2p, O 1s, and F 1s. High‐resolution Ti 2p spectra (Figure 2f) showed similar peak positions for both samples, indicating no shift in chemical states. Elemental compositions derived from XPS (Table S2) reveal a notably lower fluorine content in RISE‐MXene_1_ than MILD‐MXene [37]. This reduction is attributed to the use of H_2_O_2_ as a green oxidant, which enhances the etching kinetics and shortens the reaction time, leading to enriched surface O‐containing groups and decreased F element (Figure 2g). Hydroxyl radicals may also play a critical role in the varied termination. This surface chemistry shift is beneficial for materials performance. It has been reported that reduced F termination on the surface of MXene will mitigate hydrolysis and improve oxidative resistance [38]. Therefore, we evaluated the stability of RISE‐MXene_1_ and MILD‐MXene aqueous dispersion over the course of one week at ambient temperature by monitoring their UV–vis spectra (Figures 2h and S19). While both spectra exhibited a slight decrease in the absorbance, the absorbance of RISE‐MXene_1_ dispersion experienced only 7.4% drop whereas the MILD‐MXene showed a 10.1% drop at 327 nm. Moreover, to provide more robust and application‐relevant assessment of stability, the long‐term electrical conductivity retention of RISE‐MXene_1_ and MILD‐MXene films was evaluated over seven weeks (Figure 2i). RISE‐MXene_1_ films retained 45% of their initial conductivity, whereas MILD‐MXene films retained only 29%. This result, together with the UV–vis absorption trends, consistently demonstrates the improved ambient stability of RISE‐MXene_1_. This stability enhancement is likely due to the higher surface coverage of ─O terminations, which reduces the Fermi energy level to limit the excitation of charge carriers to the surface of MXene, thus slowing down the oxidation process. However, this may also partially diminish conductivity, a phenomenon that is consistent with our experimental results (Figure S20). Notably, mechanical testing revealed comparable stress‐strain behavior between RISE‐MXene_1_ and MILD‐MXene films (Figure S21), confirming that the oxidative etching process does not compromise mechanical integrity. Collectively, these results highlight the superior stability and comparable mechanical performance of MXenes synthesized via RISE, underscoring the practical advantages and broad application potential of this approach over the conventional MILD method.

**FIGURE 3:**
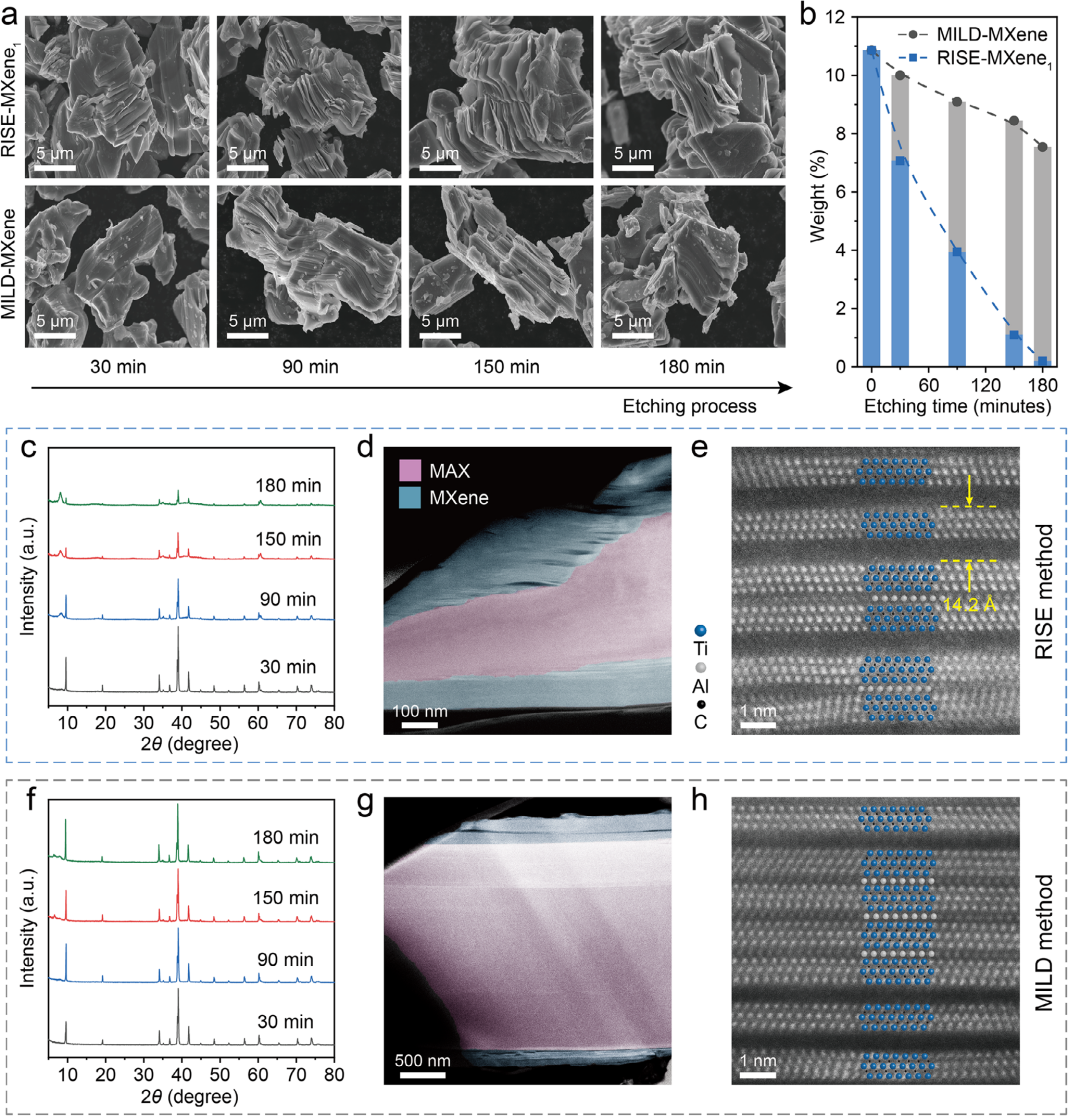
(a) SEM images of RISE‐MXene_1_ and MILD‐MXene with different etching processes. (b) The Al concentration of different etching stages according to the EDS result. (c) XRD patterns of RISE‐MXene_1_ prepared at different etching times. (d) HAADF‐STEM image and (e) corresponding atomic structure of the RISE method after etching for 90 min. (f) XRD patterns of the MILD‐MXene prepared at different etching times. (g) HAADF‐STEM image and (h) corresponding atomic structure of the MILD method after etching for 90 min.

To further elucidate the effect of H_2_O_2_ on the etching process of MXene, we examined the microstructural evolution of the MAX at various time intervals. The morphological and compositional change of RISE‐MXene_1_ and MILD‐MXene was evaluated using SEM and EDS analysis to monitor the etching process (Figure 3a, b, and Table S3). SEM images of oxidative‐etched Ti_3_AlC_2_ revealed an accordion‐like structure after only 30 min of etching, in stark contrast to its original compact bulk morphology. The concentration of Al in MAX gradually diminished while the F content increased over time. Notably, at equivalent time points, the Al residual content in RISE‐MXene_1_ was much lower than that in MILD‐MXene, indicating faster and more complete A‐layer extraction in the radical‐assisted system. We further analyzed the XRD patterns of RISE‐MXene_1_ and MILD‐MXene collected at different etching times to track the structural evolution of Ti_3_AlC_2_ during the etching process (Figure 3c, f). After etching for 30 min, a nascent, faint (002) characteristic peak appeared at 8.4° (*d*‐spacing ≈ 10.5 Å), indicating the initial formation of MXene layers. As the reaction was extended to 90 min, the (002) peak progressively shifted and intensified, aligning with the layer expansion in the SEM images (Figure 3a). After 3 h, only a tiny amount of unetched Ti_3_AlC_2_ remained. By contrast, MILD‐MXene samples retained the strong characteristic peaks of MAX across varying etching times, indicative of sluggish reaction kinetics in the absence of radical oxidants (Figure 3f). Taken together, RISE strategy enables rapid, efficient, and selective Al removal, far surpassing conventional methods in both rate and effectiveness.

HAADF‐STEM was employed to visualize the microstructural evolution of Ti_3_AlC_2_ along 90 min of etching via both the RISE and MILD methods (Figure 3d, e, g–h). STEM samples were prepared in parallel to the c‐lattice orientation of MAX using focused ion beam (FIB) for cutting. The peripheral regions of MAX particles exhibited signs of structural transformation, including cracking and exfoliation, whereas the core maintained a denser structure. The high‐magnification image of the edge region (corresponding to the etched areas) revealed the clear atomic‐scale configuration of the Ti_3_C_2_T*_x_* MXene phase (Figure 3e). During the etching process, the partial disappearance of Al atomic layers was observed, coinciding with the intercalation of Li^+^ ions and H_2_O_2_ molecules, and resulting in an expanded interlayer spacing of ∼14.2 Å. This spatial expansion is consistent with the shift in the (002) XRD reflection observed in Figure 3c. The core region shows a typical crystal structure of Ti_3_AlC_2_, indicating incomplete etching in the interior (Figure S22). The MILD etching route also demonstrated a similar stepwise etching pattern, wherein only the outermost edge regions showed evidence of cracking and etching, while the interior remained largely intact (Figures 3g, h, and S23). Of note, under similar precursor size and identical processing conditions, the residual unetched core in the MILD‐etched sample was significantly larger than that in the RISE system. This observation reflects enhanced etching efficiency for RISE approach. Post‐synthetic washing process leads to progressive delamination of etched layers, leaving a smaller core as illustrated in Figure S24. These findings support a stepwise edge‐to‐core etching mechanism, where Ti_3_AlC_2_ is gradually converted to Ti_3_C_2_T*_x_* starting from the edge toward the interior of the particles [39]. To further delineate the roles of different etchant components, a series of control experiments were conducted using H_2_O_2_ alone, H_2_O_2_/HCl, and H_2_O_2_/LiF as etchants under identical conditions (Figure S25). These three experimental conditions failed to produce the Ti_3_C_2_T*_x_* peak in XRD patterns, and SEM images also corroborated the same results. There is no delaminated MXene generated after etching and washing process, and only minor surface voids are spotted at the particle edges. These results underscore the critical synergistic role of the complete LiF/HCl/H_2_O_2_ formulation, which is essential for effective A‐layer extraction and monolayer MXene formation.

**FIGURE 4:**
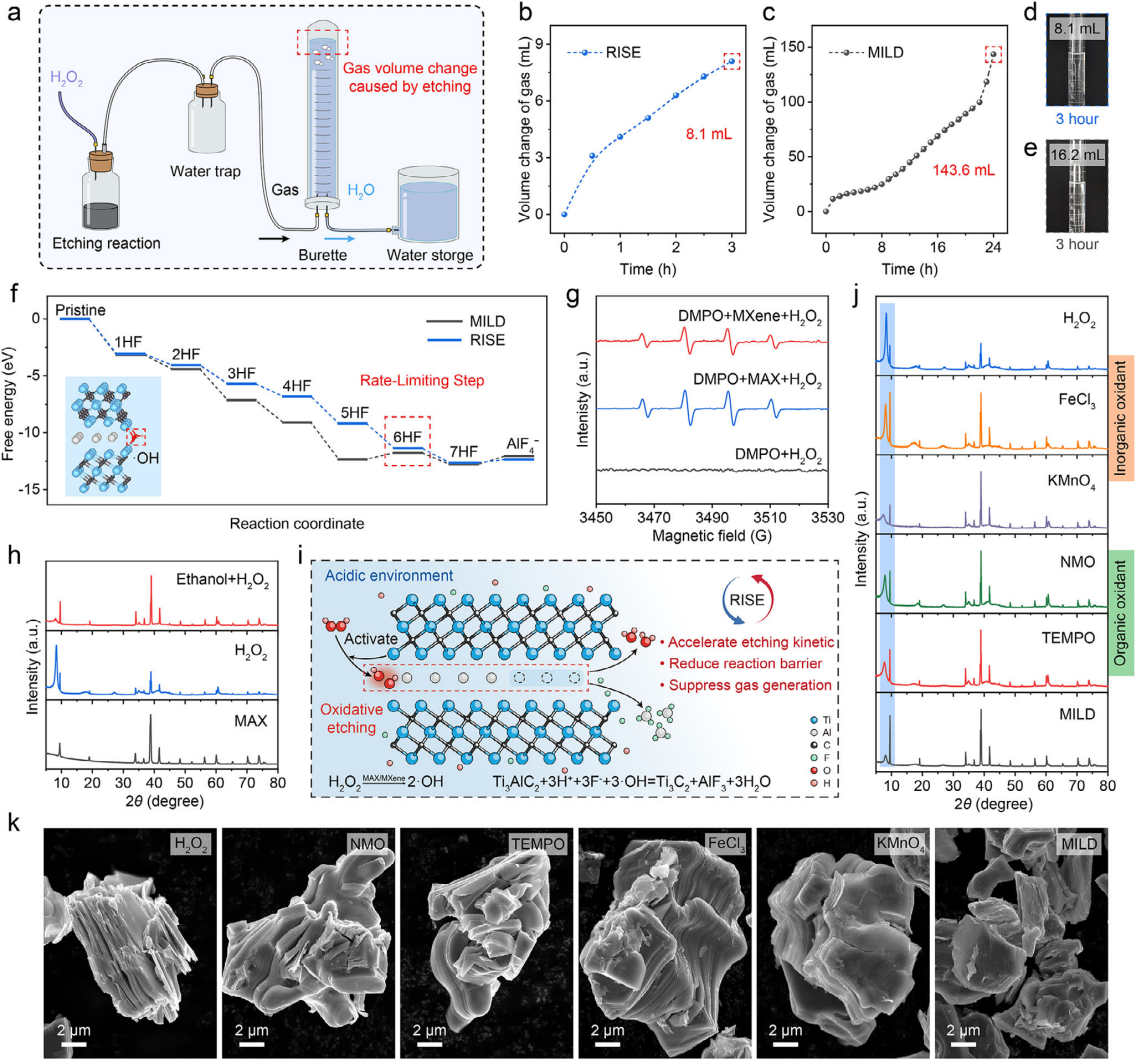
(a) Schematic illustration of the gas collection device. The change in gas volume of (b) RISE and (c) MILD strategies. Photographs of the gas volume produced by the (d) RISE and (e) MILD routes etching for 3 h. (f) Δ*G* for both etching routes at different stages. (g) EPR spectra of DMPO+H_2_O_2_, DMPO+MAX+H_2_O_2_, and DMPO+MXene+H_2_O_2_ solutions. (h) XRD patterns of the MAX, MXene etching with H_2_O_2_ and ethanol+H_2_O_2_ for 3 h. (i) Schematic illustration of the mechanism of RISE process. (j) XRD patterns and (k) SEM images of MAX etching with different oxidants for 3 h.

To gain deeper mechanistic insights into the etching process, in particular the role of oxidants in the removal of Al layers, a customized gas collection setup was designed to monitor the evolution of hydrogen (H_2_) gas, a byproduct indicative of reductive reactions involving Al in the MAX phase (Figure 4a) [28]. In the conventional MILD protocol, the removal of the A‐layer (e.g., Al) involves direct electron transfer between Al atoms and H^+^. This redox process reduces H^+^ to H_2_ gas while oxidizing metallic Al to Al^3+^, often accompanied by aggressive gas evolution and heat release. As shown in Figure 4b, c, the cumulative gas volume produced during etching was recorded for both the RISE and the MILD protocols. A sharp contrast was observed: while the MILD route resulted in a substantial release of H_2_ gas, the etching system incorporating H_2_O_2_ as an auxiliary oxidant produced only 8.1 mL of gas, highlighting a significant suppression of proton‐mediated reduction reactions (Figure 4d, e). This marked difference in gas production suggests that H^+^ is not the sole electron acceptor involved in the oxidation of Al layers. While a minor amount of gas is generated in the initial stage, likely due to early‐stage reaction between Al and H^+^ in the acidic medium, the rapid involvement of H_2_O_2_ as a more potent oxidant soon dominates the redox process through a non‐gas‐generating pathway.

Density functional theory (DFT) calculations were performed to clarify the atomic‐scale etching mechanism. The computational model structure of MAX was comprised of two layers of Ti_3_C_2_ and an intermediate layer of Al atoms as shown in Figure S26. To simulate the etching process, the Ti and Al atoms at the left edge of the MAX structure were immobilized, while the remaining atoms were allowed to completely relax to realistically model surface reactions and subsequent structural changes during etching [12, 40]. In the RISE etching route, H_2_O_2_ molecules interact with the exposed edge of MAX structure, followed by sequential attack by HF molecules. The electron localization function (ELF) highlighted the changes in chemical bonding throughout the oxidative etching stages (Figure S27). In the initial stage of the reaction, the H_2_O_2_ dissociated into two reactive **·**OH radicals and subsequently coordinated with exposed Ti1 and Ti2 atoms at the edge. Subsequently, HF molecules sequentially approached the activated edges, readily dissociating into H^+^ and F^−^. These ions aggressively attacked both Al and adjacent Ti atoms, significantly weakening and eventually breaking the Ti─Al bonds. Therefore, the Ti atoms became mobile and the interlayer gallery was opened by the terminations, facilitating further ingress of reactive species. Figures S28 and S29 present the DFT calculations of the MILD etching process and corresponding ELF plots. Thermodynamic analysis via calculated Gibbs free energy (Δ*G*) changes indicated that the RISE mechanism proceeded spontaneously and exothermically, whereas the MILD mechanism exhibited an endothermic barrier at the sixth HF insertion step (Figure 4f). At this critical reaction stage, Al atoms reacted with F^−^ ions to yield soluble AlF_4_^–^ complexes, accompanied by the breaking of Ti–Al bonds. Concurrently, the **·**OH radicals served as efficient electron acceptors, accepting electrons from Al atoms and subsequently reacting with H^+^ to form H_2_O. Despite the Δ*G* of MILD etching being more negative at certain steps, the rate‐limiting reaction step governed the overall etching kinetics. In contrast, the RISE pathway avoids such a strong uphill step. Critically, the **·**OH participated as an efficient electron acceptor and oxidant, lowering the effective barrier for A‐layer removal and enabling faster net kinetics. This illustrates that overall reaction rate is governed not by the thermodynamic favorability of individual intermediates, but by the highest free‐energy barrier along the reaction coordinate, which is a key distinction that reconciles the Δ*G* profiles in Figure 4f with the experimentally observed ultrafast etching of the RISE method. Based on the experiments and calculations, the etching reaction can be expressed as:

(1) $$Ti_{3}\mathrm{Al}C_{2}+3\mathrm{HCl}+3\mathrm{LiF}\to Ti_{3}C_{2}+\mathrm{Al}F_{3}+3\mathrm{LiCl}+\frac{3}{2}H_{2}$$

(2) $$Ti_{3}\mathrm{Al}C_{2}+3\mathrm{HCl}+3\mathrm{LiF}+\frac{3}{2}H_{2}O_{2}\to Ti_{3}C_{2}+\mathrm{Al}F_{3}+3\mathrm{LiCl}+3H_{2}O$$

The overall etching process involves two keys Equations (1) and (2), where both H^+^ and H_2_O_2_ participate in the redox reaction to oxidize the metallic Al atoms in Ti_3_AlC_2_ to the Al^3+^ state. The **·**OH, as a strong electrophilic radical, exhibits stronger oxidative potential and enhanced electron‐accepting capability than H^+^. Therefore, reaction pathway involving **·**OH (i.e., Equation (2)) becomes kinetically dominant, thereby dictating the overall etching rate in the RISE system.

To further clarify the underlying reaction mechanism, electron paramagnetic resonance (EPR) spectroscopy was employed to detect the generation of reactive oxygen species (ROS), particularly **·**OH radicals, arising from H_2_O_2_ decomposition during etching. As shown in Figure 4g, no EPR signal was observed when the spin‐trapping agent 5,5‐dimethyl‐1‐pyrroline N‐oxide (DMPO) was mixed with H_2_O_2_ alone, indicating that H_2_O_2_ is relatively stable at room temperature and does not spontaneously generate radicals under ambient conditions. Upon introduction of either MAX or MXene into the system under acidic conditions to simulate the etching process, distinct signals ascribed to **·**OH radical were clearly detected. These phenomena suggest that, MAX and MXene act as Fenton‐like catalysts to decompose H_2_O_2_ into radicals during the etching process [41]. The resulting **·**OH radicals accelerate the etching reaction to realize synergistic etching. To validate the functional role of **·**OH radicals in the etching process, ethanol, a known radical scavenger, was introduced to replace H_2_O in the etching system. The resulting XRD pattern closely resembled that of MILD‐etched samples, with pronounced Ti_3_AlC_2_ peaks and minimal MXene formation, thus confirming the essential role of **·**OH radicals in enabling efficient etching (Figure 4h).

A schematic of the proposed RISE mechanism is accordingly presented in Figure 4i. During the etching process, the Ti_3_AlC_2_ MAX phase or partially etched MXene surface acts as a Fenton‐like catalyst to facilitate the in situ decomposition of H_2_O_2_ to generate highly reactive **·**OH radicals. These reactive species, in synergy with F^−^, simultaneously attack and oxidize the Al atomic layers, promoting rapid oxidation of Al to Al^3+^ and subsequent formation of soluble Al‐F complexes. The implications of this shift in redox chemistry are twofold. First, the involvement of **·**OH as a primary oxidant accelerates the etching kinetics by significantly lowering the activation energy barrier, as corroborated by DFT calculations. The enhanced electron transfer capability of **·**OH, relative to H^+^, facilitates more efficient bond cleavage at the Ti–Al interface, thereby accelerating A‐layer extraction and structural delamination. Second, the reduction in gas volume enhances operational safety and scalability by mitigating the risks associated with pressurized H_2_ buildup in sealed or batch processing environments, a concern that is often overlooked in wet chemical MXene synthesis. Taken together, these results provide compelling evidence that the RISE mechanism introduces a distinct and more controlled reaction pathway compared to traditional acidic etching methods, offering a more sustainable and inherently safer synthetic route for high‐yield monolayer MXene production. Expanding on this, a range of alternative oxidants was employed for selective etching of MAX under identical etching conditions. Figure 4j displays the XRD patterns of the MAX etched with various oxidants and LiF/HCl mixtures. Although all oxidants promoted some degree of Al layer removal, the H_2_O_2_‐based system consistently yielded the strongest MXene (002) diffraction peaks, signifying the highest degree of conversion from MAX to MXene. Corresponding SEM image of the H_2_O_2_‐etched product revealed a characteristic lamellar morphology with expanded interlayer spacing and abundant crevices, confirming the optimal etching effect (Figure 4k). These findings collectively confirm that the RISE route operates a synergistic and kinetically favorable mechanism, wherein H_2_O_2_ is catalytically decomposed by MAX/MXene to generate highly reactive **·**OH radicals that serve as potent oxidants to drive rapid A‐layer removal. Overall, RISE redefines the role of H_2_O_2_ in MXene synthesis: Rather than serving merely as a single‐function oxidant or dominant etchant, H_2_O_2_ acts as an in situ radical precursor that generates **·**OH species to drive a radical‐mediated etching pathway distinct from previous reports [42, 43]. This controlled and efficient mechanism enables rapid, high‐yield, and scalable production of monolayer MXenes with improved safety, positioning RISE as a practical platform for MXene manufacturing.

**FIGURE 5:**
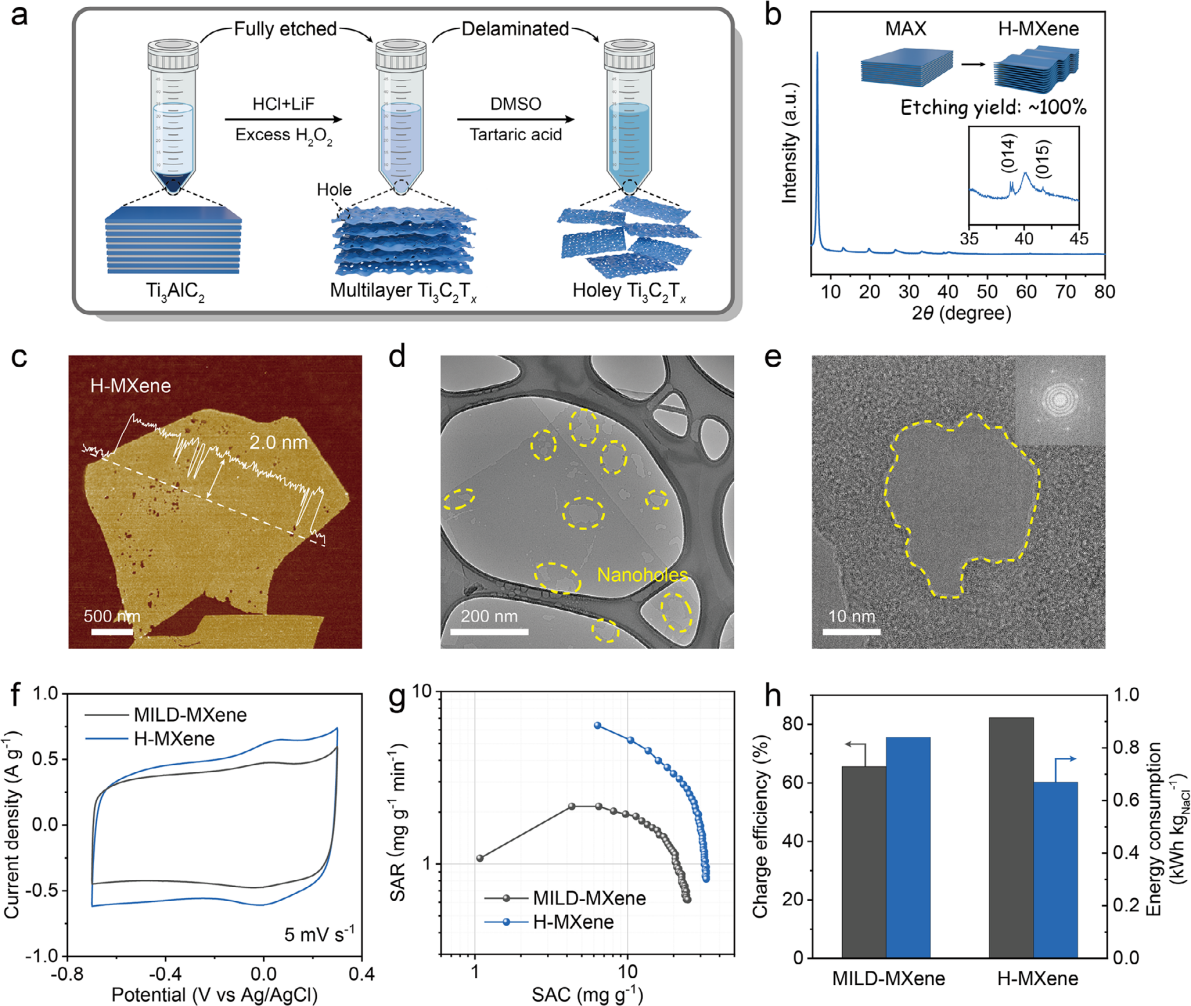
(a) Schematic illustration of the H‐MXene synthesis process. (b) XRD patterns of H‐MXene after washing with DMSO and tartaric acid. (c) AFM images of H‐MXene. (d) TEM image of a monolayer H‐MXene flake. (e) HR‐TEM image of H‐MXene flake and corresponding FFT image. (f) CV curves of H‐MXene and MILD‐MXene films at 5 mV s^−1^. (g) CDI Ragone plots, (h) charge efficiencies and energy consumptions of H‐MXene and MILD‐MXene films in 500 mg L^−1^ NaCl solution at 1.2 V.

In our previous studies, excess H_2_O_2_ was found to oxidize the MXene surface to form TiO_2_, which can subsequently be removed by tartaric acid to yield holey Ti_3_C_2_T*_x_* nanosheets with excellent colloidal stability [44, 45]. Along this research line, we investigated the feasibility of one‐step preparation of MXene with tailorable in‐plane structure via RISE strategy. Figure 5a shows the scheme of the process for creating holey Ti_3_C_2_T*_x_* bearing customized in‐plane holes (defined as H‐MXene). During the etching process, **·**OH initially facilitated the etching of Al layers. Meanwhile, excess **·**OH can react with exposed Ti atoms on the surface to form TiO_2_. To maximize delamination and purification, MXene was sequentially washed with DIW, DMSO, and aqueous tartaric acid. DMSO was introduced to enhance intercalation, while tartaric acid selectively removed TiO_2_ byproduct, yielding H‐MXene with abundant in‐plane holes. Unlike the two‐step process reported previously, which requires separate MXene synthesis and subsequent oxidation/perforation, the RISE strategy achieves a one‐step MAX‐to‐H‐MXene conversion by simply tuning the H_2_O_2_ dosage. In this process, the nanoholes are generated in situ during etching, without any post‐synthetic perforation step being required [46, 47, 48]. The XRD pattern of H‐MXene (Figure 5b) reveals a complete transformation of the precursor, that is, a quantitative etching yield. The characteristic (104) peaks of Ti_3_AlC_2_ have nearly vanished, and the (002) peak has shifted from 9.5° to 6.6°, indicative of a MAX‐to‐H‐MXene transformation. The etching yield of H‐MXene, including multilayer and monolayer Ti_3_C_2_T*_x_*, in the final products was quantitatively estimated from XRD using a fitting formula reported by Mashtalir et al.: [49]

(3) $$y=1-0.2x+0.013x^{2}$$

Where, *y* represents the mass fraction of Ti_3_C_2_T*_x_*, and *x* is the diffraction intensity ratio of the Ti_3_AlC_2_ (104) peak to the Ti_3_C_2_T*_x_* (002) peak (peak area to represent the diffraction intensity). The etching yield of H‐MXene through excess H_2_O_2_ etching was calculated to be 99.9%, suggesting near‐quantitative conversion of the MAX phase to H‐MXene. Raman spectra confirmed the absence of TiO_2_, validating the efficacy of tartaric acid treatment in selectively removing oxidized Ti species (Figure S30). Only the characteristic vibrational modes of Ti_3_C_2_T*_x_* were observed, corroborating the purity of the final product. Besides, AFM and TEM were used to visualize the microscopic morphologies of H‐MXene. Monolayers with uniformly distributed in‐plane nanopores of *ca*. 20 nm in diameter, alongside a well‐preserved hexagonal crystal lattice, confirmed the high structural integrity of the holey MXene (Figure 5c–e).

In general, the in‐plane nanoholes are highways for ion transport, which effectively improves the performance and potential of MXene for electrochemical applications [50]. To evaluate the practical implications of the engineered porosity, the MILD‐MXene and H‐MXene films were prepared by vacuum‐filtration deposition and employed directly as working electrodes. Electrochemical properties were first assessed through cyclic voltammetry (CV) in 1 M NaCl aqueous electrolyte at scan rates ranging from 2 to 50 mV s^−1^ (Figures 5f and S31).

The quasi‐rectangular and symmetric CV profiles suggest the typical pseudocapacitive behavior. Notably, the integral area of the CV curve for the H‐MXene surpasses that of MILD‐MXene, indicating the larger specific capacitance. Specifically, the calculated specific capacitance of H‐MXene is constantly greater than that of MILD‐MXene at all scan rates and achieves a high value of 116.9 F g^−1^ at 2 mV s^−1^ (Figure S32), which is indicative of the positive effect of nanoholes on ion accessibility and storage. In addition, the galvanostatic charge/discharge (GCD) curves of H‐MXene display quasi‐symmetric triangular shapes with longer discharge time than that of MILD‐MXene (Figures S33 and S34), which further indicates the enhanced capacitive performance of H‐MXene and is in good agreement with the CV results. Overall, the remarkable pseudocapacitive performance of H‐MXene underscores its promise as an advanced electrode material, in particular for electrosorption desalination and other ion storage‐driven applications.

A batch‐model CDI cell was constructed to assess the practical desalination performance of H‐MXene and MILD‐MXene films (Figure S35). As depicted in Figure S36a, the conductivity of the NaCl solution decreased rapidly in the initial adsorption period and tended to be saturated after 40 min under an applied potential of 1.2 V. Subsequent application of a reverse potential led to a recovery in conductivity, reflecting the good reversibility of ion intercalation and deintercalation processes. Importantly, the H‐MXene electrode exhibits a lower equilibrated conductivity (946.8 µS cm^−1^) than that of MILD‐MXene (961.0 µS cm^−1^), demonstrating its superior capability of ion removal. Consequently, the calculated salt adsorption capacity (SAC) of H‐MXene (32.71 mg g^−1^) is much higher than that of MILD‐MXene (24.81 mg g^−1^) (Figure S36b). This enhanced performance is further illustrated in the Ragone plot (Figure 5g), where H‐MXene occupies the upper‐right quadrant, indicative of both high desalination capacity and rapid ion adsorption kinetics. According to the current transient curve (Figure S37), the charge efficiency of H‐MXene is determined to be 82.3%, which is higher than that of MILD‐MXene (65.6%). The corresponding energy consumptions of H‐MXene and MILD‐MXene are 0.67 and 0.84 kWh kg_NaCl_^−1^, respectively (Figure 5h). These improvements can be directly attributed to the engineered in‐plane nanoholes within the H‐MXene nanosheets, which provide rapid ion transport pathways and increased accessible surface area for ion adsorption. This structural advantage facilitates more efficient charge transfer and accelerates desalination dynamics, highlighting the superiority of the RISE strategy for developing high‐performance CDI electrodes. We further evaluated the electrochemical performance of H‐MXene in H_2_SO_4_ electrolyte and subsequently fabricated a H‐MXene‐based micro‐supercapacitor (Figures S38–S40). The device delivers a high volumetric capacitance of 445.8 F cm^−3^ at 10 mV s^−1^, accompanied by excellent rate capability and cycling stability (Figure S41). Collectively, these proof‐of‐concept studies suggest that the RISE‐enabled porosity, together with preserved conductivity, could benefit other electrochemical energy‐storage architectures by enhancing ion accessibility and transport [51, 52].

## 3 Conclusion

In summary, we established a highly efficient RISE strategy that enables the one‐step synthesis of monolayer MXene nanosheets with customized in‐plane nanoholes within hours. By precisely modulating the concentration of H_2_O_2_ in a LiF/HCl medium, we simultaneously accelerated Al layer removal and regulated in‐plane structure, enabling a high monolayer yield of 81.6% within 3 h under mild conditions (40° C), with the lateral flake size of 4.1 ± 2.4 µm. Remarkably, when employing excess H_2_O_2_, the etching protocol achieves a near‐quantitative conversion (99.9%) of Ti_3_AlC_2_ to Ti_3_C_2_T*_x_* while introducing in‐plane nanoholes, realizing the first high‐yield fabrication of H‐MXene and ensuring maximal material utilization. The engineered nanopores significantly enhance ion accessibility and diffusion, translating into superior electrochemical performance in CDI. H‐MXene electrodes exhibit a high salt adsorption capacity of 32.71 mg g^−1^ in 500 mg L^−1^ NaCl solution at 1.2 V. Furthermore, the method is broadly applicable to other MAX phases (e.g., Ti_3_CNT*_x_*, Ti_2_CT*_x_*) and scalable to a gram‐level, making it suitable for industrial adoption. Mechanistically, a combination of gas evolution analysis, EPR spectroscopy, and DFT calculation reveals that the RISE strategy follows a distinct redox pathway from traditional proton‐driven route. The involvement of **·**OH facilitates faster A‐layer removal, reduces hydrogen gas evolution, and enables in situ structural engineering. Altogether, this study presents a paradigm shift in monolayer MXene synthesis by merging processing efficiency with structural precision, providing valuable insights for the development of swift synthesis methods for diverse MXene materials in future research endeavors.

## Author Contributions

**Chenxu Liu**: writing – original draft, methodology, investigation, data curation, and visualization. **Hao Zhang**: writing – original draft, investigation, and formal analysis. **Anirban Sikdar**: formal analysis. **Kanglei Pang**: data curation. **Guangyuan Ma**: software. **Kai Xi**: resources, project administration. **Shujiang Ding**: writing – review and editing, and validation. **Jiayin Yuan**: conceptualization, writing – review and editing, and investigation. **Miao Zhang**: conceptualization, writing – review and editing, supervision, and funding acquisition.

## Conflicts of Interest

The authors declare no conflicts of interest.

## Supporting information




**Supporting File 1**: anie72450‐sup‐0001‐SuppMat.docx.

## Data Availability

The data that support the findings of this study are available from the corresponding author upon reasonable request.
