# Supplementary material for: One‐Step Radical‐Intensified Selective Etching (RISE) Strategy for High‐Yield Synthesis of Monolayer MXene with Tailored Nanoholes
Source: Angew Chem Int Ed Engl. 2026 May 4;65(25):e9523099. doi: 10.1002/anie.9523099 (PMC13266965; doi:10.1002/anie.9523099)
Supplement: Supplementary file 1 — Supporting File 1: anie72450‐sup‐0001‐SuppMat.docx. [file ANIE-65-e9523099-s001.docx]

Supporting Information

One-step Radical-intensified Selective Etching (RISE) Strategy for High-yield Synthesis of Monolayer MXene with Tailored Nanoholes

Chenxu Liu,^[a]^ Hao Zhang,*^[a]^ Anirban Sikdar,^[b]^ Kanglei Pang,^[b]^ Guangyuan Ma,^[a]^ Kai Xi,^[a]^ Shujiang Ding,*^[a]^ Jiayin Yuan,*^[b]^ and Miao Zhang*^[a]^

[a] C. Liu, Dr. H. Zhang, G. Ma, Prof. K. Xi, Prof. S. Ding, Prof. M. Zhang
School of Chemistry
Xi’an Jiaotong University
Xi’an 710049, P. R. China
E-mail: zhanghao1997@xjtu.edu.cn
 dingsj@mail.xjtu.edu.cn
 miaozhangchem@xjtu.edu.cn

[b] Dr. A. Sikdar, Dr. K. Pang, Prof. J. Yuan
Department of Chemistry
Stockholm University
Stockholm 10691, Sweden
E-mail: jiayin.yuan@su.se

Experimental section

**1. Materials**

Ti_3_AlC_2_ powder (400 mesh, 98%) was purchased from Laizhou Kai Kai Ceramic Materials Co., Ltd. Lithium fluoride (LiF, 99.9%), lithium hydroxide (LiOH, anhydrous, 98%), sodium chloride (NaCl, 99.5%), dimethyl sulfoxide (DMSO, 99%), and tartaric acid (ACS, ≥ 99.5%) were obtained from Shanghai Aladdin Biochemical Technology Co., Ltd. Hydrogen peroxide (H_2_O_2_, 30 wt%) and hydrochloric acid (HCl, 36.0-38.0 wt%) were purchased from Sinopharm Chemical Reagent Co., Ltd. All reagents were used as received without further purification.

**2. Method**

**2.1 Synthesis of RISE-MXene_x_**

The RISE-MXene_x_ was prepared by selectively etching the Al layers of the MAX precursor using the RISE method. Briefly, 1.5 g of LiF was added to 20 mL of 9 M HCl under stirring. Subsequently, 1 g of MAX and a controlled volume of 30 wt% H_2_O_2_ were added to the solution in six equal portions to suppress the rapid self-decomposition of H_2_O_2_. After the etching reaction at 40 °C for 3 h, the product was repeatedly washed with deionized water (DIW) and collected *via* centrifugation until the pH of the supernatant reached 6. Finally, the supernatant was collected by centrifugation following 30 min of shaking and ultrasonication to obtain delaminated RISE-MXene_x_ nanosheets. The conventional MILD-MXene control sample was prepared under identical conditions without the addition of H_2_O_2_, except that the etching temperature was 35 °C and the reaction time was 24 h. The monolayer yield of MXene was determined by the equation as follows:

$$\text{Yield}=\frac{\text{m}_{\text{MXene}}}{\text{m}_{\text{MAX}}}\times\text{100\%}$$

where m_MXene_ is the mass of monolayer MXene, m_MAX_ represents the mass of original MAX.^[1]^

**2.2 Synthesis of H-MXene**

H-MXene was prepared via the RISE protocol with an H_2_O_2_ dosage of 1.5 mL per batch. The etching solution was washed 5-6 times with DIW until the pH exceeded 6. Subsequently, the product was transferred to DMSO and stirred for 12 h. After washing 5 times with DIW, 20 g of tartaric acid was added to the mixture, which was shaken for 2 h. After five additional DIW washing cycles, delaminated H-MXene nanosheets were collected via sequential shaking, ultrasonication (30 min), and centrifugation.

**2.3 Fabrication of H-MXene MSC (micro-supercapacitor)**

MXene interdigital electrodes were fabricated *via* a mask-assisted vacuum filtration process of a H-MXene dispersion (0.1 mg mL^−1^) through a PTFE membrane. The patterned electrode was then transferred onto a PET substrate at 15 MPa and dried in a vacuum oven at 40 °C for 12 h. Separately, a PVA-H_2_SO_4_ gel electrolyte was prepared by dissolving 1 g of PVA in 10 mL of water at 90 °C for 4 h, followed by the dropwise addition of 1 g of concentrated H_2_SO_4_ upon cooling. The gel electrolyte was applied to the electrode surface, and the device was packed with Kapton and copper tape.

**3. Characterization**

X-ray diffraction (XRD) patterns were obtained using a Bruker D8 ADVANCE X-Ray diffractometer equipped with a Cu Kα radiation source. The thicknesses of MXene nanosheets were measured by atomic force microscopy (AFM, Veeco Instruments, CA) utilizing a Nanoscope V in tapping mode. The morphology of products was examined by field-emission scanning electron microscopy (SEM, GeminiSEM 500, ZEISS) and transmission electron microscopy (Talos F200X, Thermo Fisher). High-angle annular dark-field scanning transmission electron microscopy characterization (HAADF-STEM) and corresponding energy-dispersive X-ray spectroscopy (EDS) elemental mapping were performed on Talos F200X (Thermo Fisher) equipped with an energy-dispersive spectroscopy detector. The atomic structure of the samples was investigated by scanning transmission electron microscopy (JEM-ARM200F NEOARM, JEOL). Samples for STEM were prepared using a focused ion beam (FIB, FEI Helios Nanolab 600i) system. X-ray photoelectron spectroscopy (XPS) measurements were conducted on ESCALAB Xi+ (Thermo Fisher) with Al Kα radiation. The Raman spectra were recorded from 100 cm^−1^ to 4000 cm^−1^ using InVia Qontor spectrometer (Renishaw). The zeta potential measurements of MXene dispersion were carried out using a Zetasizer Nano ZS90 (Malvern Panalytical). The lateral size distributions were collected across multiple randomly selected regions; over 100 individual flakes were measured for each sample. Tensile stress-strain curves were obtained using a universal testing machine (CMT1102, SASCK) equipped with a 100 N load cell at a loading rate of 1 mm min^−1^. The electrical conductivity of the MXene films was obtained on a four-probe resistivity tester (RTS-8, 4Probes Tech Ltd.).

**4. Electrochemical characterization**

The electrochemical characterization was performed on a CHI 760E electrochemical workstation (Shanghai CH Instruments Co.). The electrochemical measurements of H-MXene and MILD-MXene films were performed in a three-electrode configuration using 1.0 M NaCl and 0.5 M H_2_SO_4_ as the aqueous electrolyte, respectively. MXene-based films were cut into strips with an effective contact area of 1.5 × 1.0 cm^2^ and directly used as the working electrodes. An Ag/AgCl electrode served as the reference electrode and a platinum plate was used as the counter electrode. The specific capacitance (*C_s_*, F g^−1^) was calculated from cyclic voltammetry (CV) curves according to:

$$C_{s}=\int\frac{IdV}{2mv\Delta V}$$

where *I* (A) is the current, *v* (mV s^−1^) is the scan rate, Δ*V* (V) is the potential window, and *m* (g) stands for the mass of the electrode material.

The two-electrode configuration was applied to test the H-MXene MSC. The areal capacitance (*C_a_*, mF cm^−2^) was calculated from cyclic voltammetry (CV) curves according to:

$$C_{a}=\int\frac{IdV}{2Av\Delta V}$$

The volumetric capacitance (*C_v_*, F cm^−3^) was calculated from cyclic voltammetry (CV) curves according to:

$$C_{v}=\int\frac{IdV}{2Uv\Delta V}$$

where *I* (A) is the current, *v* (mV s^−1^) is the scan rate, Δ*V* (V) is the potential window, *A* (cm^−2^) is the area of the electrode, and *U* (cm^−3^) is the volume of the electrode.

**5. Capacitive Deionization (CDI) measurements**

The CDI cell consisted of two acrylic support plates, freestanding MXene film electrodes, and ion exchange membranes (IEMs) positioned near the current collectors. The as-prepared MXene-based films with a diameter of 3.8 cm were directly pasted onto the conductive carbon paper as binder-free electrodes. During the CDI measurement, 50 mL of 500 mg L^−1^ NaCl solution was circulated through the cell at a flow rate of 25 mL min^−1^ using a peristaltic pump. A conductivity meter (EC-400F, Shanghai INESA Scientific Instrument Co., Ltd.) was utilized to record the real-time conductivity of the NaCl solution. The conductivity can be converted to the corresponding NaCl concentration through mathematical calculations. The salt adsorption capacity (SAC, mg g^−1^) and salt adsorption rate (SAR, mg g^−1^ min^−1^) of the electrodes were calculated according to the following equations:

$$\text{SAC}=\frac{\left(C_{0}-C_{e}\right)V}{m}$$

$$\text{SAR}=\frac{\text{SAC}}{t}$$

where *C*_0_ and *C*_e_ (mg L^−1^) are the initial and equilibrated NaCl concentrations, respectively, *V* (L) is the volume of the NaCl solution, *m* (g) is the total effective mass of the film electrodes, and *t* (min) is the desalination time.

The charge efficiency (Λ) and energy consumption (*E*, kWh kg_NaCl_^−1^) were calculated according to the following equations:

$$\Lambda=\frac{\Gamma\times F}{\Sigma}$$

$$E=\frac{U\int I(t)\text{d}t}{m_{\text{NaCl}}}$$

where Γ (mol g^−1^) is the desalination capacity, *F* is the Faraday constant (96485 C mol^−1^), and Σ (charge, C g^−1^) is the charge consumption and obtained by integrating current during the desalination process, *U* is the applied potential (V), *I*(*t*) is the current (A), and *m*_NaCl_ is the mass of removed NaCl.

**6. Density functional theory (DFT) calculation**

Calculations based on first-principles density functional theory (DFT), including molecular dynamics simulations, were performed utilizing the Vienna Ab initio Simulation Package (VASP)^[2]^ in conjunction with the Projector Augmented Wave (PAW) methodology.^[3]^ The exchange-correlation functional was managed within the parameters of the Generalized Gradient Approximation (GGA), adopting the Perdew-Burke-Ernzerhof (PBE) functional.^[4]^ The long-range van der Waals interactions were accounted for using the DFT-D3 approach.^[5]^ We implemented a plane wave basis set with an energy cutoff of 500 eV. The geometric relaxation proceeded until the forces on each atom were less than 0.03 eV Å^-1^. The sampling of the Brillouin zone was conducted using a 3 × 2 × 1 k-point grid. To assure rigorous consistency, calculations were performed until the energy convergence threshold was less than 10^−5^ eV. To effectively isolate periodic structures and preclude their interaction, a vacuum buffer of 15 Å was inserted along the z-axis.

The free energy (∆G) of the intermediates is calculated as follows:

$$\Delta G=\Delta E_{DFT}+\Delta ZPE-T\Delta S$$

where ΔE_DFT_, ΔZPE, and ΔS are the changes of the reaction energy obtained from DFT calculations, zero-point energy, and the changes of entropy from the initial state to the final state, respectively. T is temperature and the T of 298.15 K was used in all computations.

**7. Supplementary Figures**


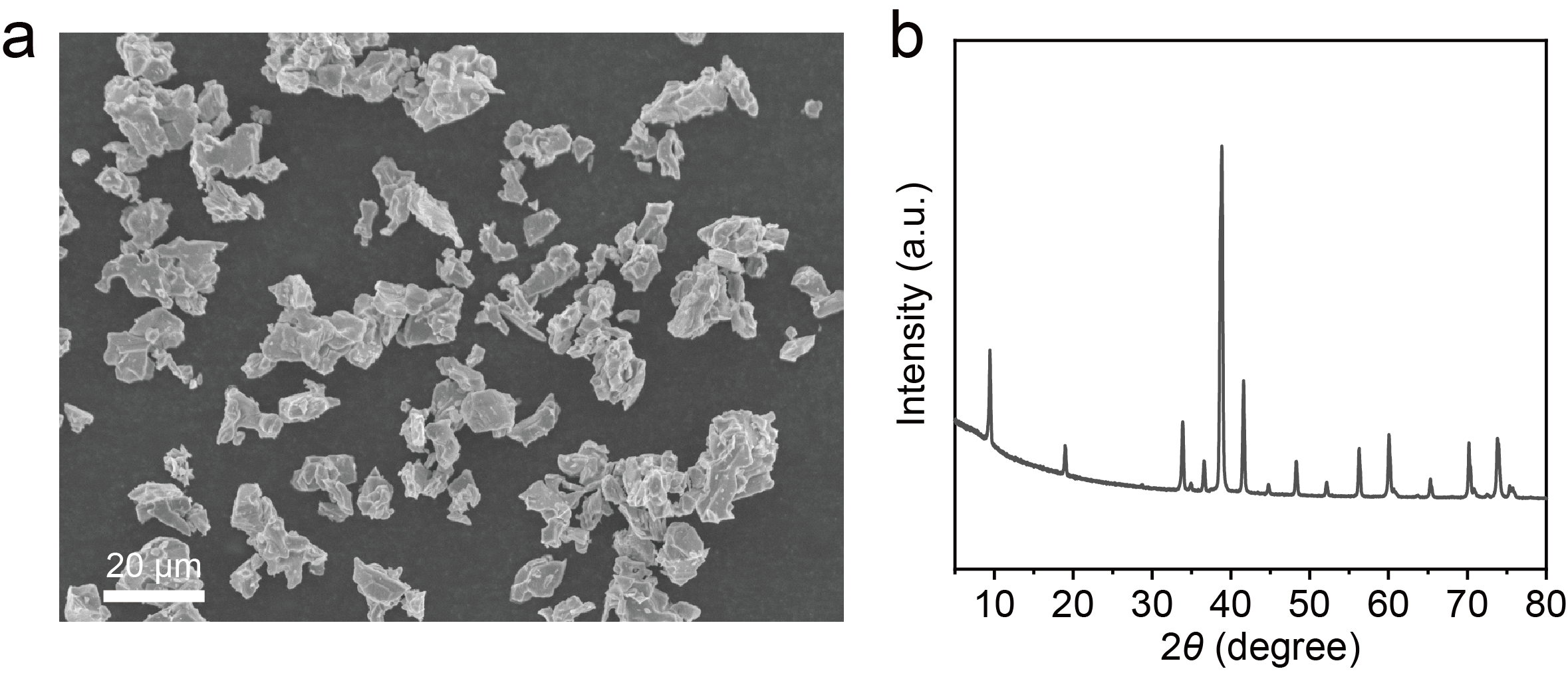


**Figure S1.** (a) SEM image and (b) XRD pattern of pristine Ti_3_AlC_2_ MAX powders.


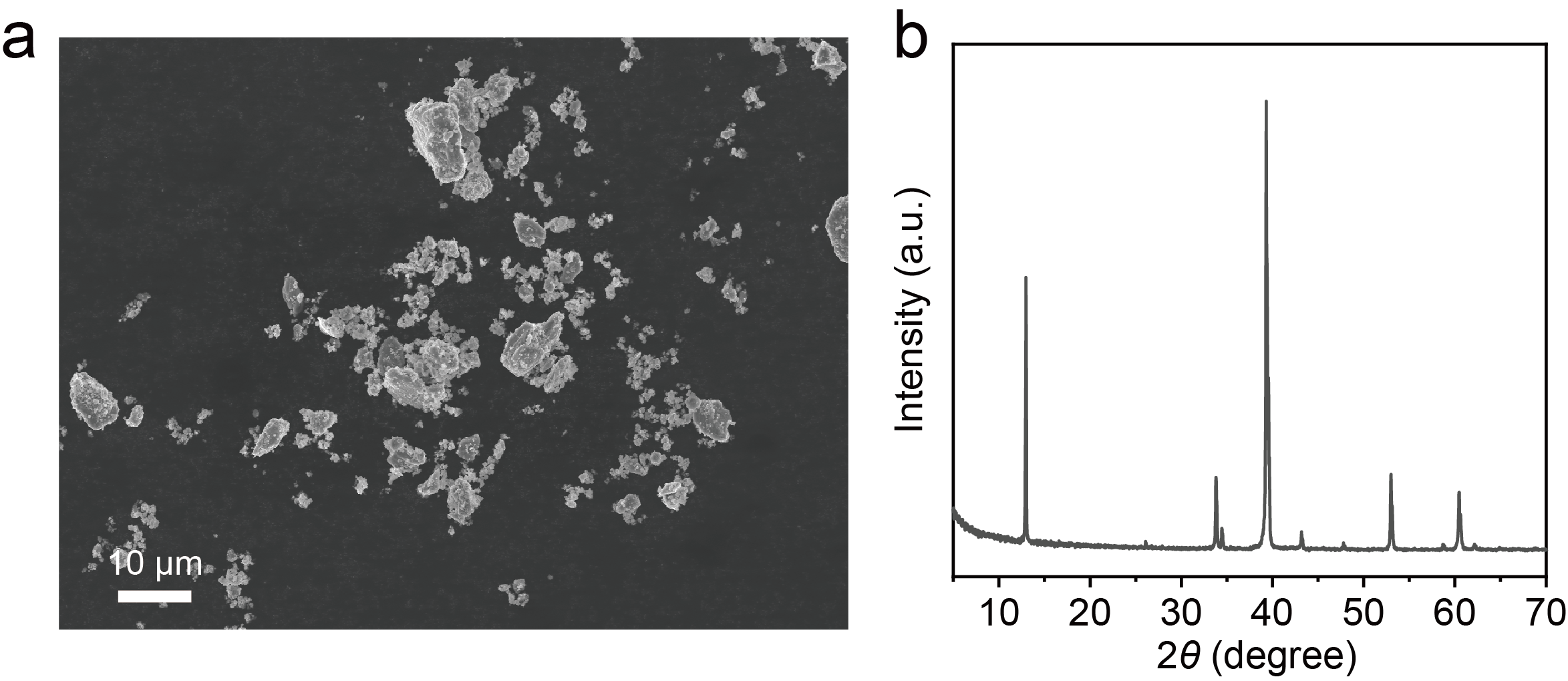


**Figure S2.** (a) SEM image and (b) XRD pattern of pristine Ti_2_AlC MAX powders.


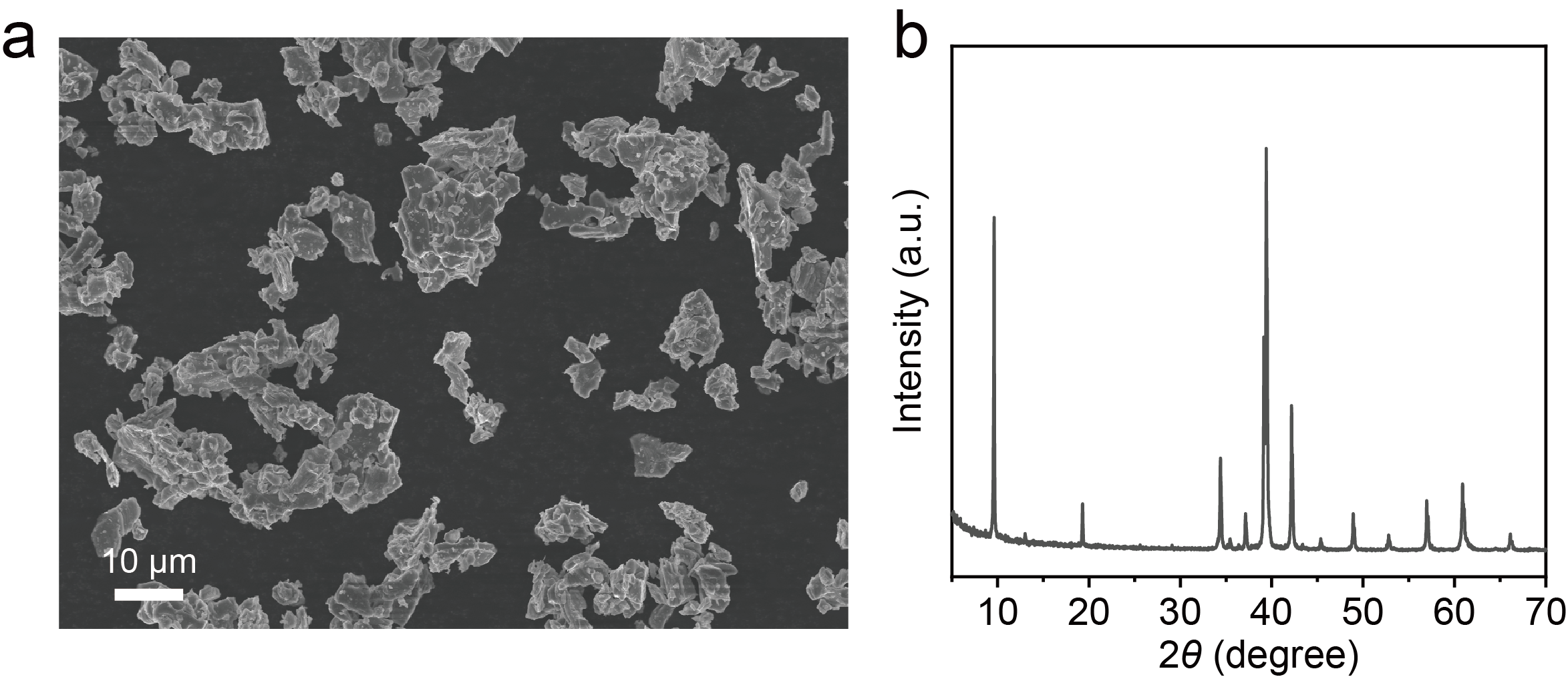


**Figure S3.** (a) SEM image and (b) XRD pattern of pristine Ti_3_AlCN MAX powders.


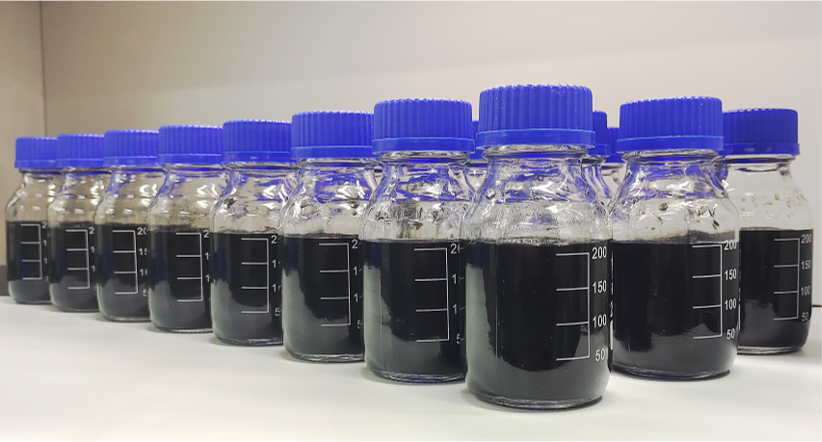


**Figure S4.** A photograph of scaled-up MXene dispersions in 200-mL bottles with a concentration of 1 mg mL^−1^.


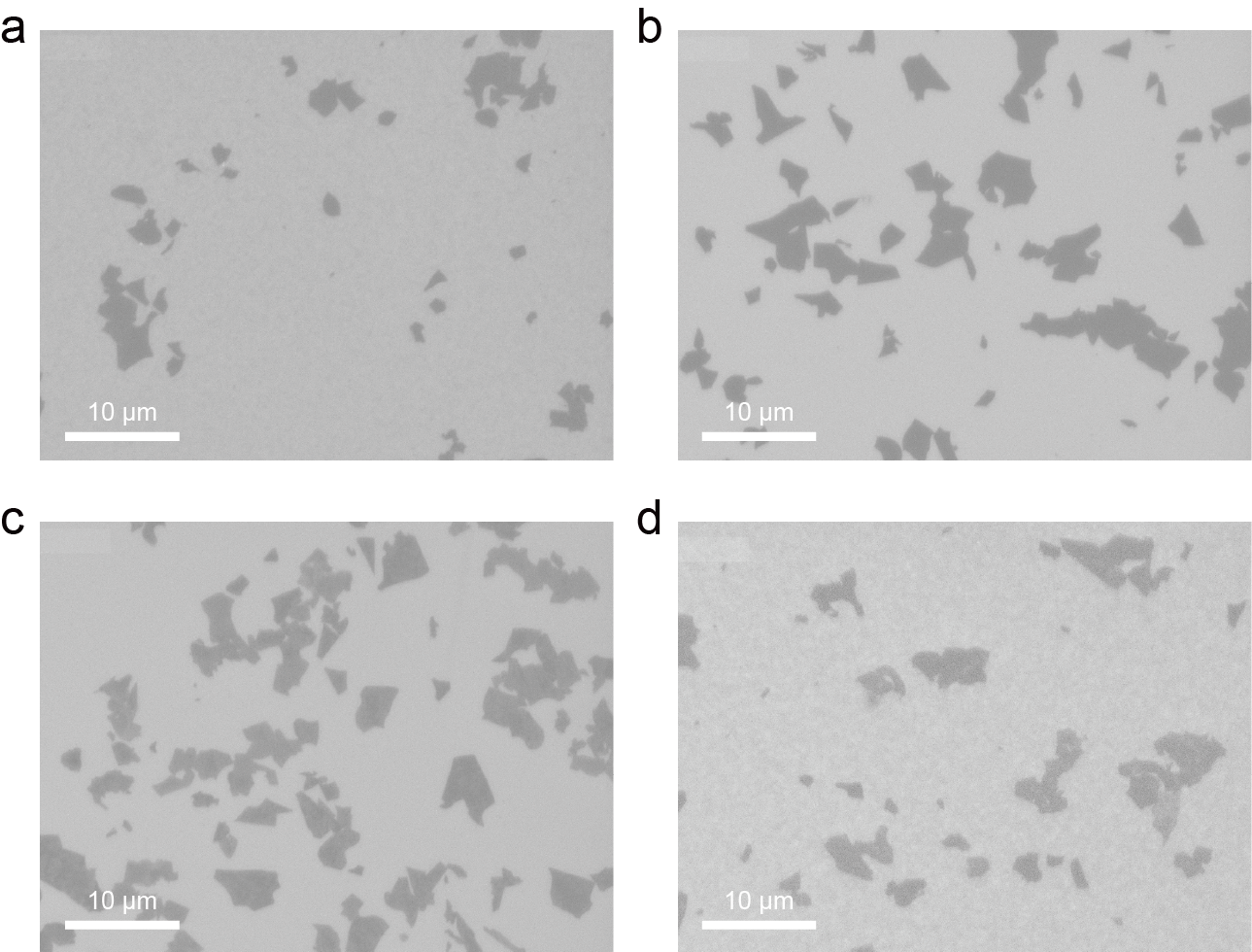


**Figure S5.** (a-d) Typical SEM images of RISE-MXene_0_, RISE-MXene_0.5_, RISE-MXene_1_, and RISE-MXene_1.5_ nanosheets prepared under varying H_2_O_2_ dosages.


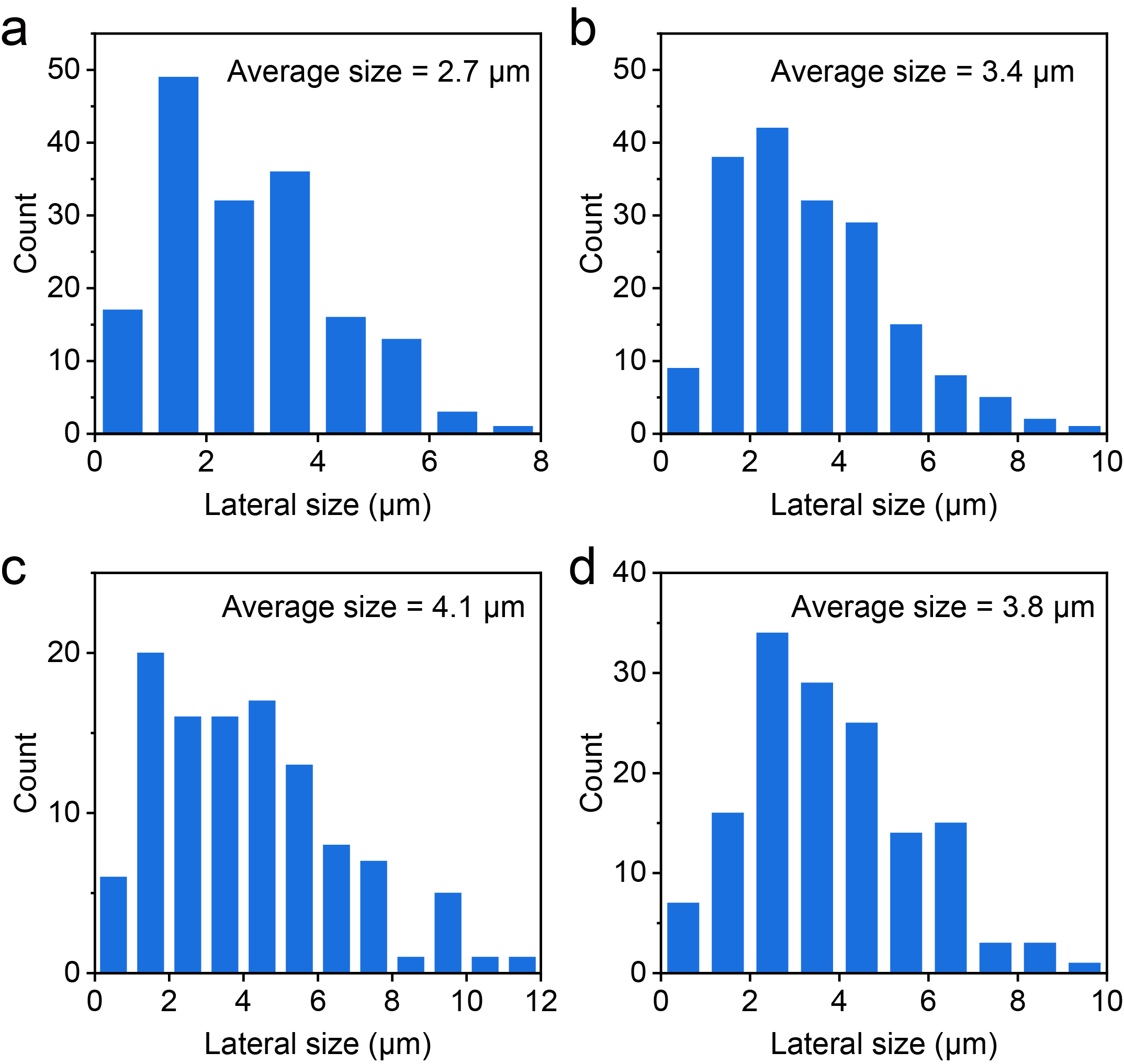


**Figure S6.** Lateral size distributions of (a) RISE-MXene_0_, (b) RISE-MXene_0.5_, (c) RISE-MXene_1_, and (d) RISE-MXene_1.5_ nanosheets. Statistics for each sample were derived from over 100 individual flakes.


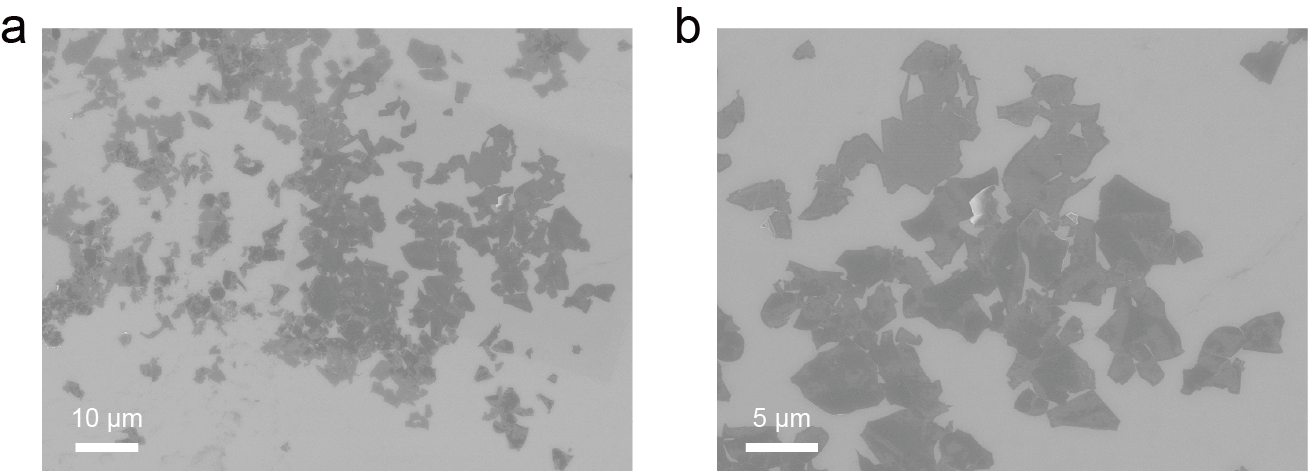


**Figure S7.** SEM images of Ti_3_CNT*_x_* flakes obtained *via* the RISE method.


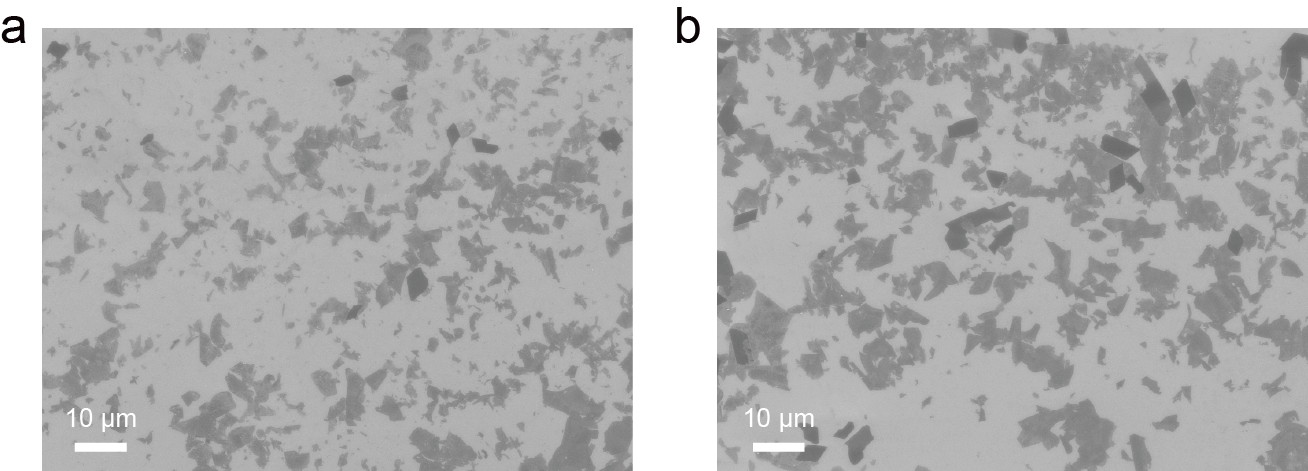


**Figure S8.** SEM images of Ti_2_CT*_x_* flakes obtained *via* the RISE method.


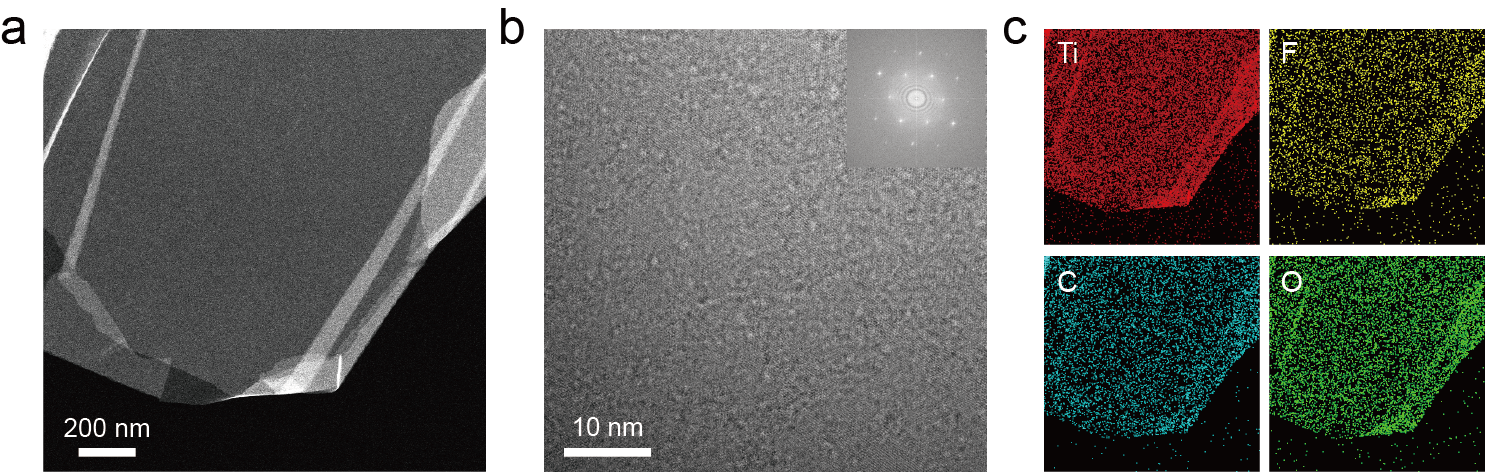


**Figure S9.** (a) HAADF-STEM image, (b) High-resolution TEM image, and (c) EDS mapping images of RISE-MXene_1_.


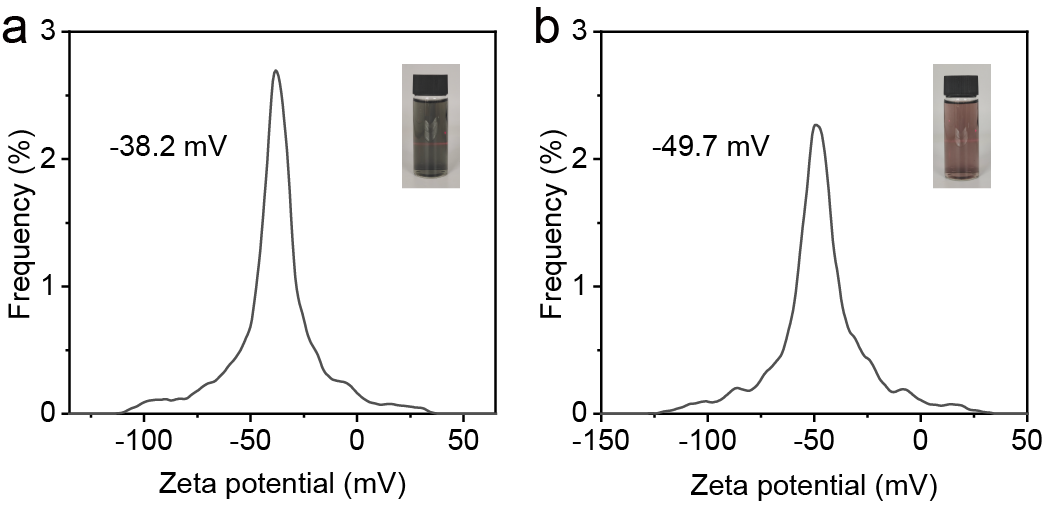


**Figure S10.** Zeta potential and corresponding Tyndall effect of (a) Ti_3_CNT*_x_* and (b) Ti_2_CT*_x_* aqueous dispersions.


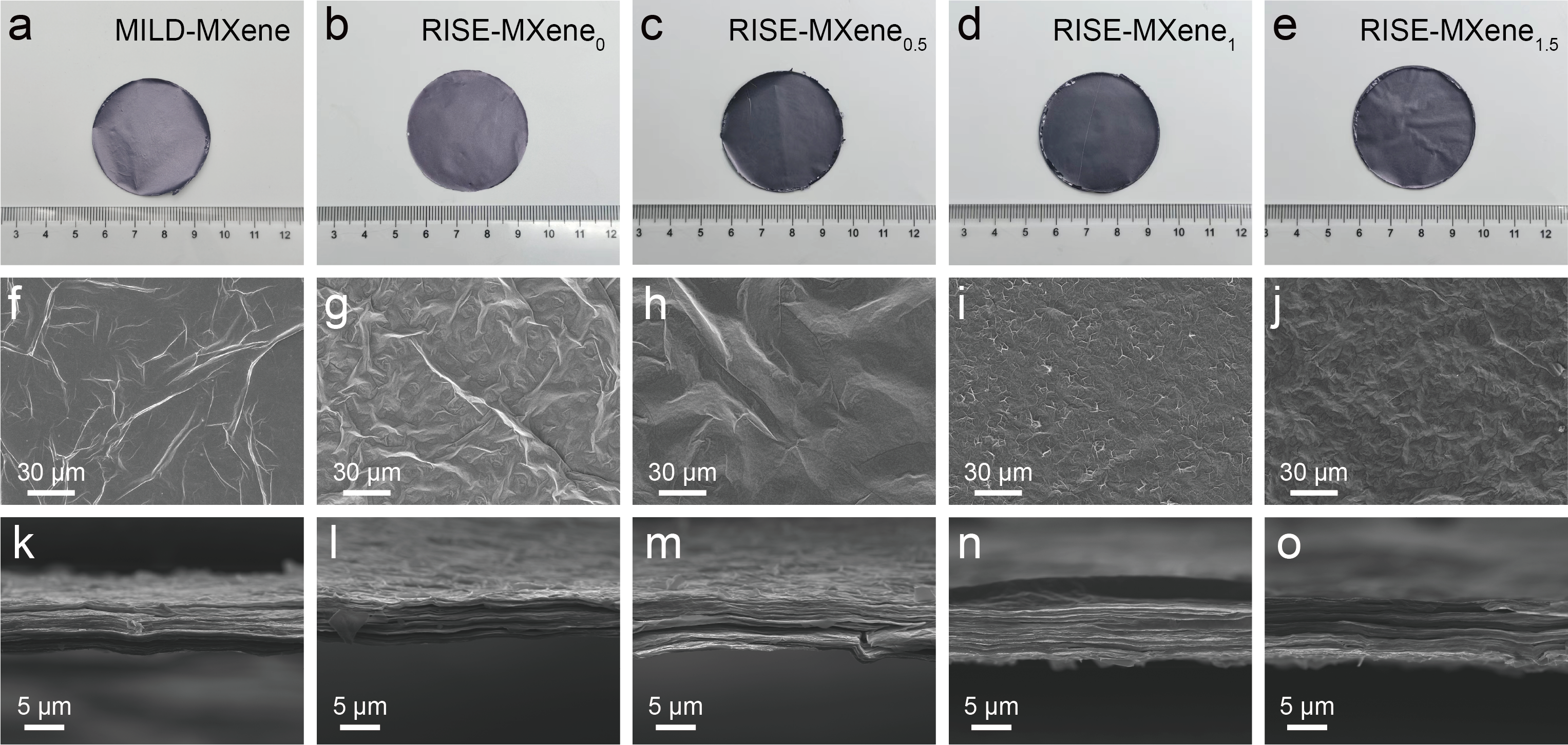


**Figure S11.** Optical photographs, surface SEM images, and cross-sectional SEM images of freestanding MXene films: (a, f, k) MILD-MXene, (b, g, l) RISE-MXene_0_, (c, h, m) RISE-MXene_0.5_, (d, i, n) RISE-MXene_1_, and (e, j, o) RISE-MXene_1.5_.


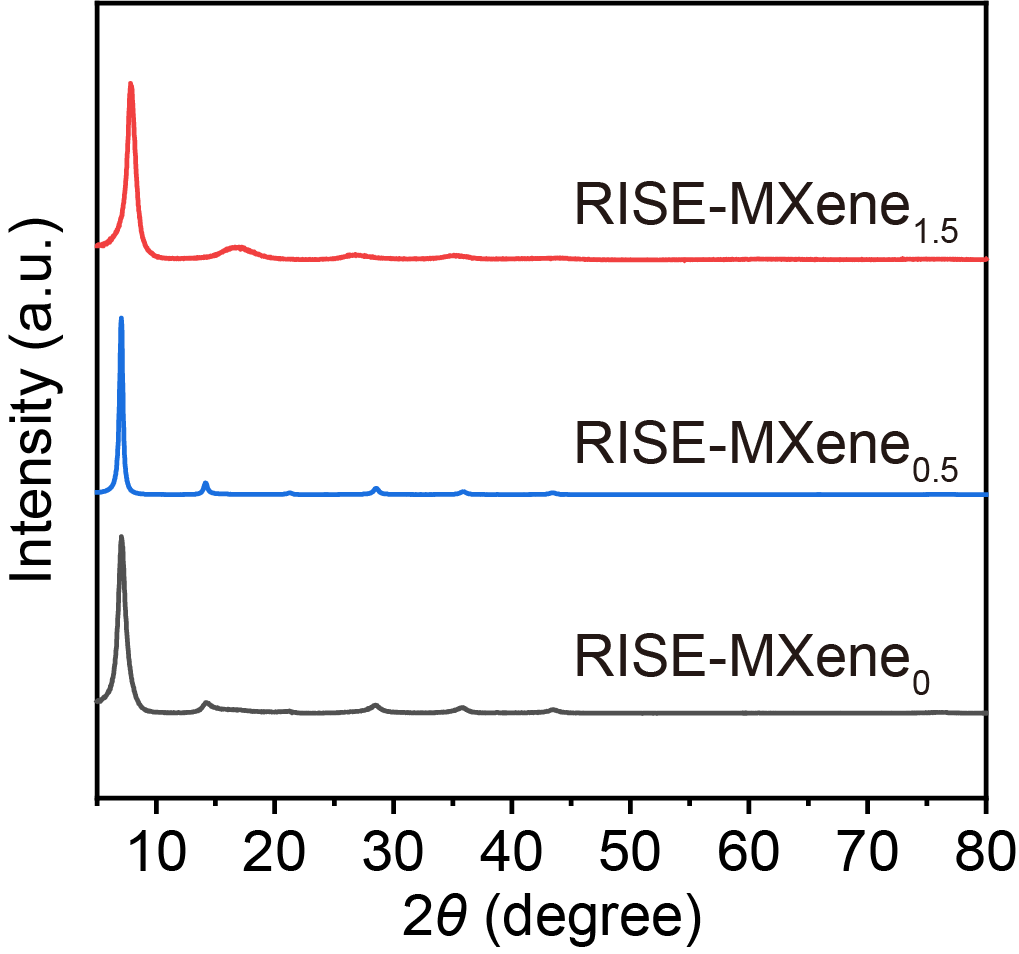


**Figure S12.** XRD patterns of RISE-MXene_0_, RISE-MXene_0.5_, and RISE-MXene_1.5_ films.\


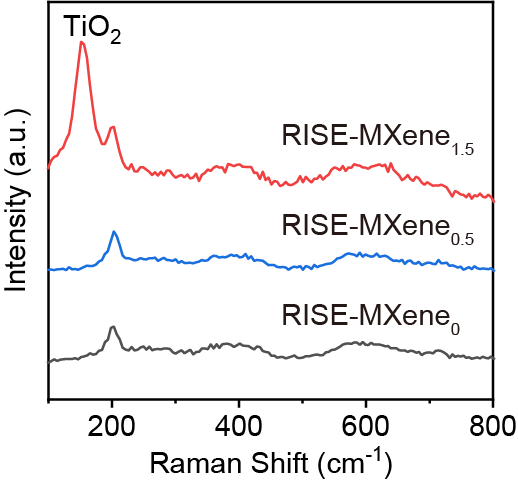


**Figure S13.** Raman spectra of RISE-MXene_0_, RISE-MXene_0.5_, and RISE-MXene_1.5_.


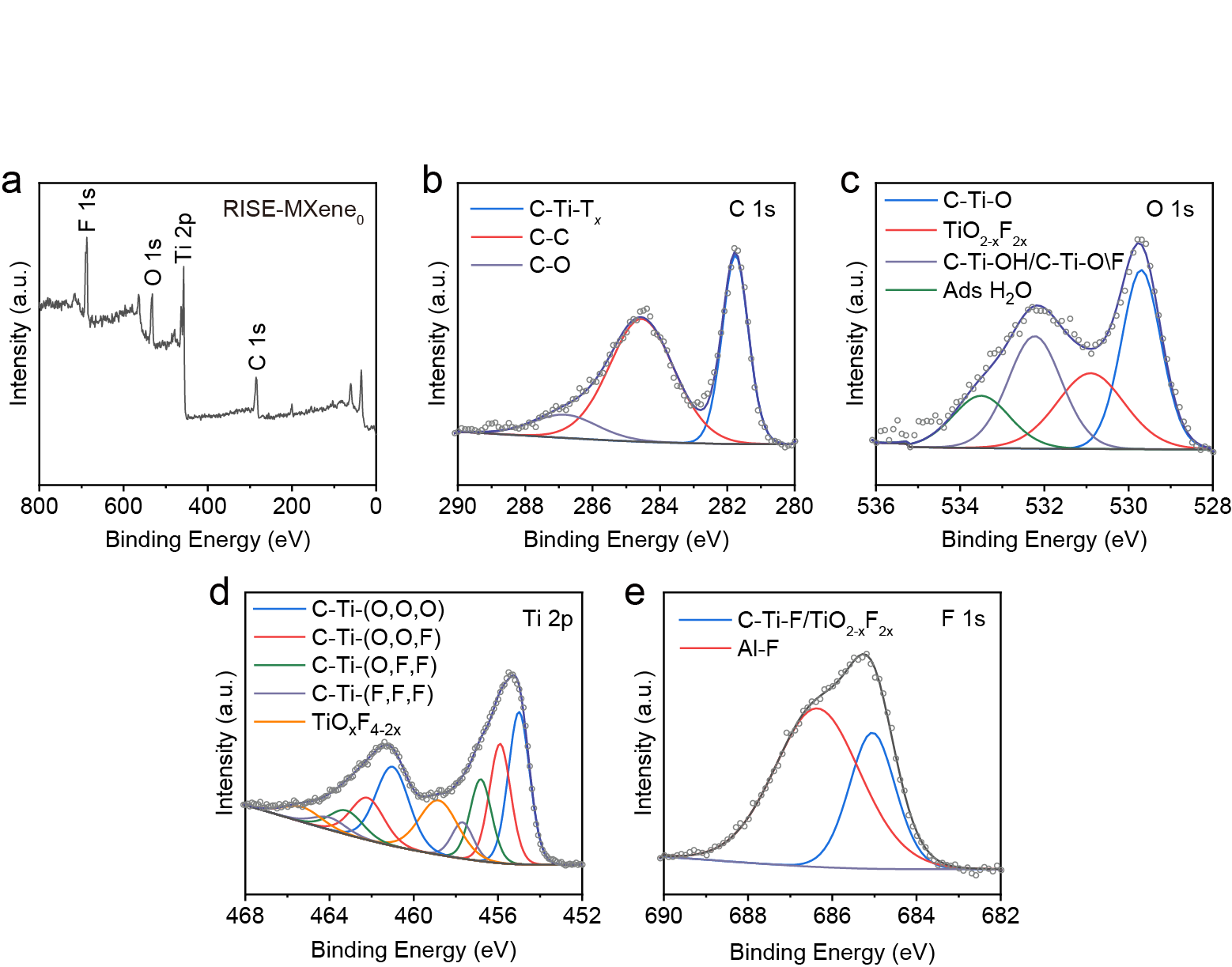


**Figure S14.** XPS analysis of RISE-MXene_0_: (a) survey spectrum and high-resolution spectra of (b) C 1s, (c) O 1s, (d) Ti 2p, and (e) F 1s with fitting curves.


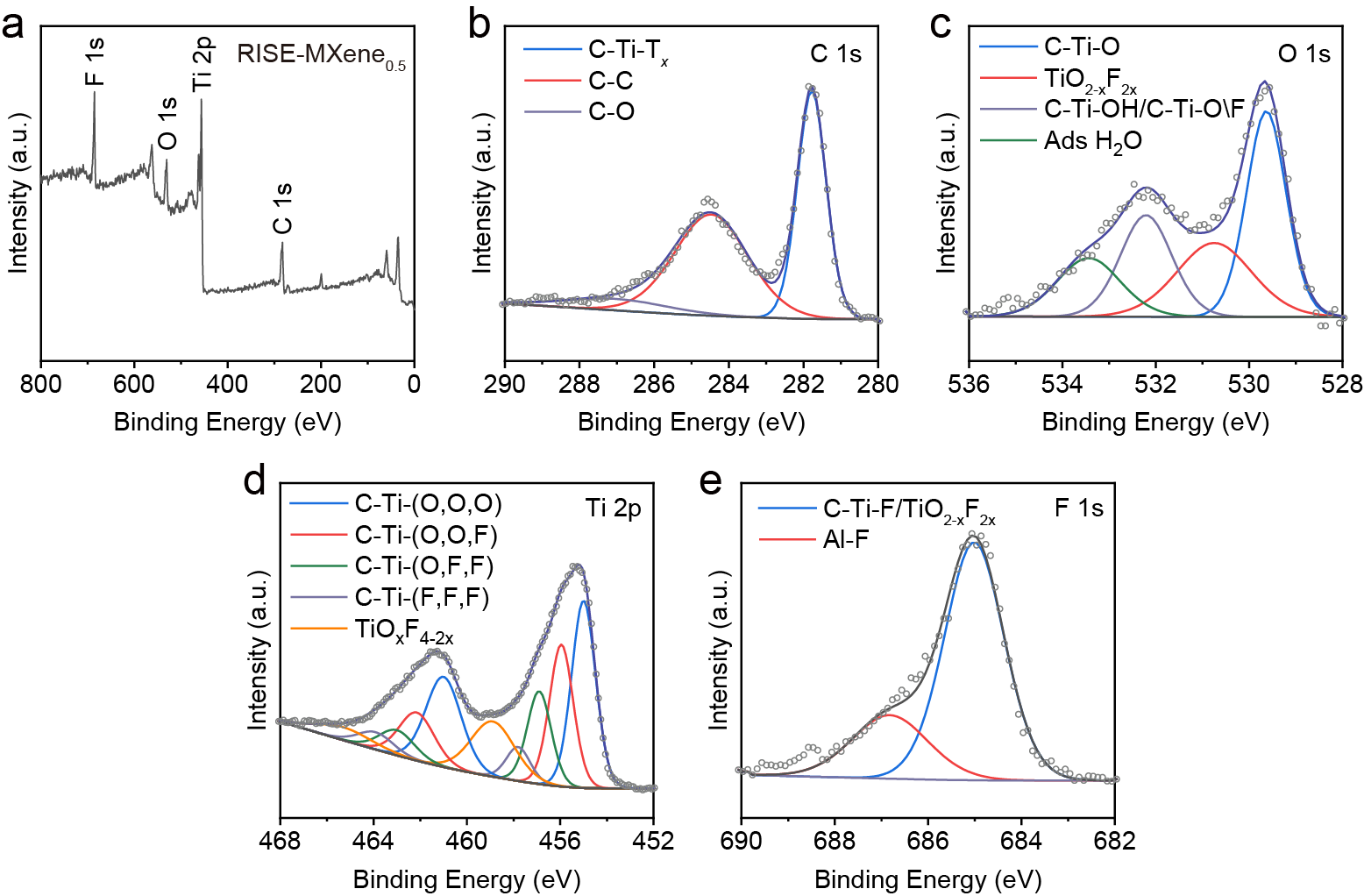


**Figure S15.** XPS analysis of RISE-MXene_0.5_: (a) survey spectrum and high-resolution spectra of (b) C 1s, (c) O 1s, (d) Ti 2p, and (e) F 1s with fitting curves.


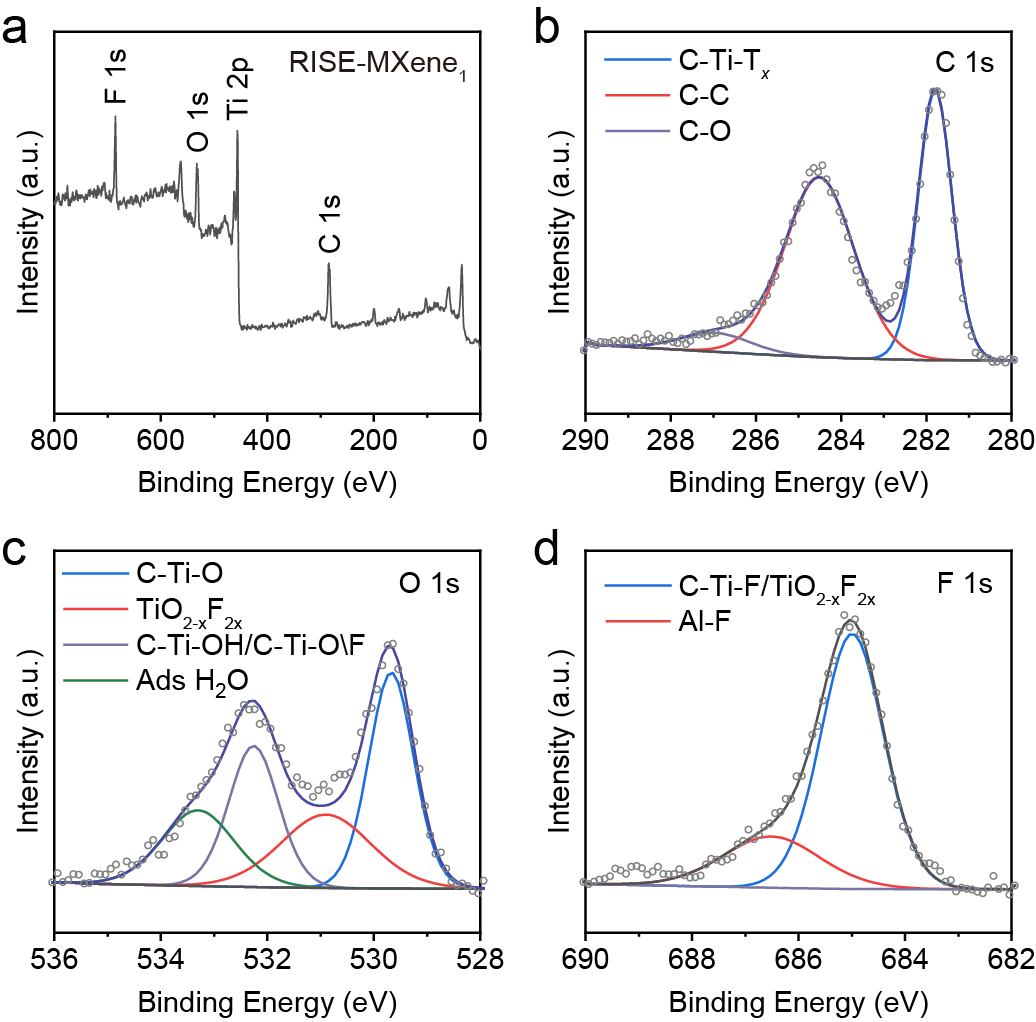


**Figure S16.** XPS analysis of RISE-MXene_1_: (a) survey spectrum and high-resolution spectra of (b) C 1s, (c) O 1s, and (d) F 1s with fitting curves.


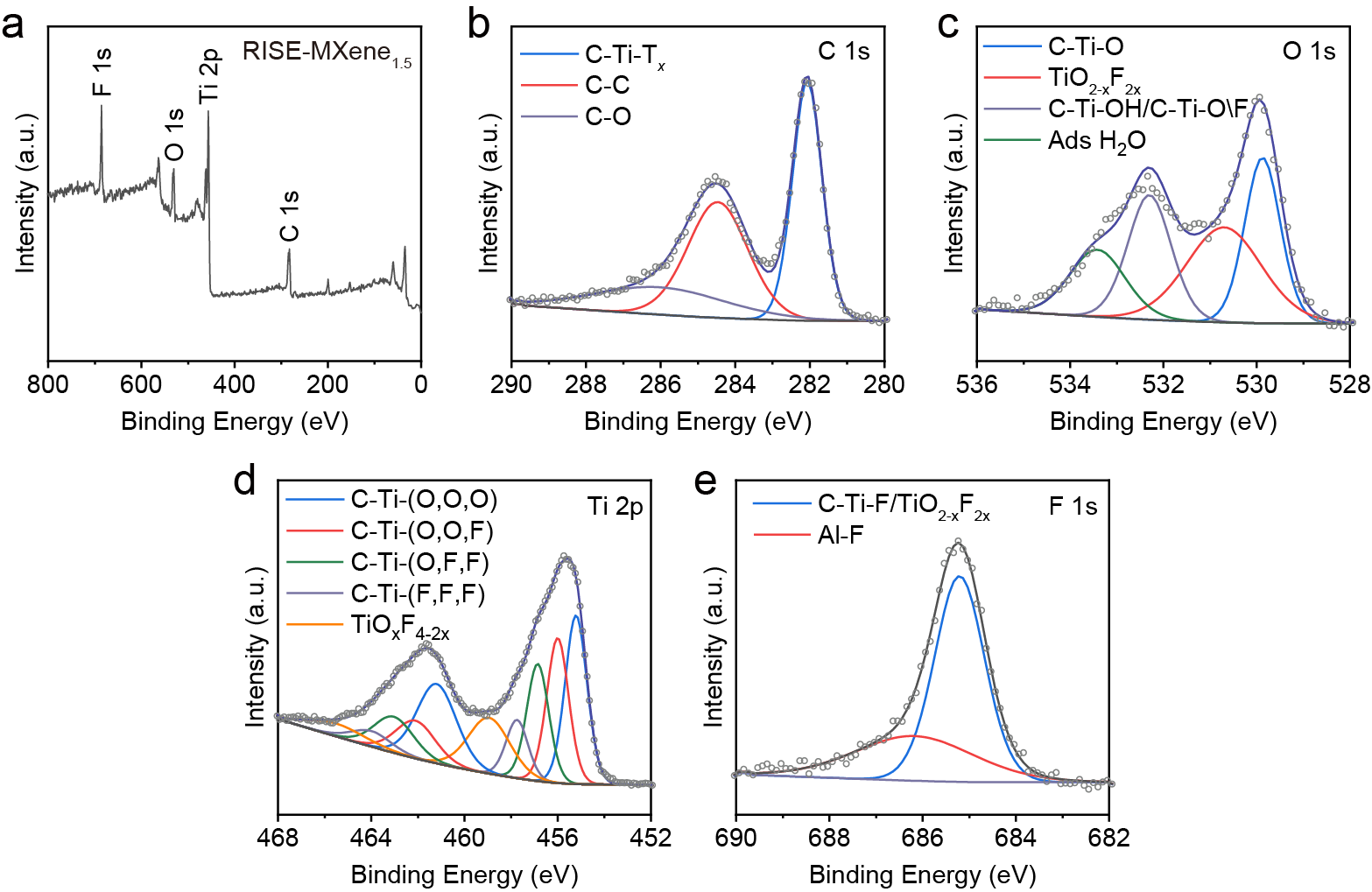


**Figure S17.** XPS analysis of RISE-MXene_1.5_: (a) survey spectrum and high-resolution spectra of (b) C 1s, (c) O 1s, (d) Ti 2p, and (e) F 1s with fitting curves.


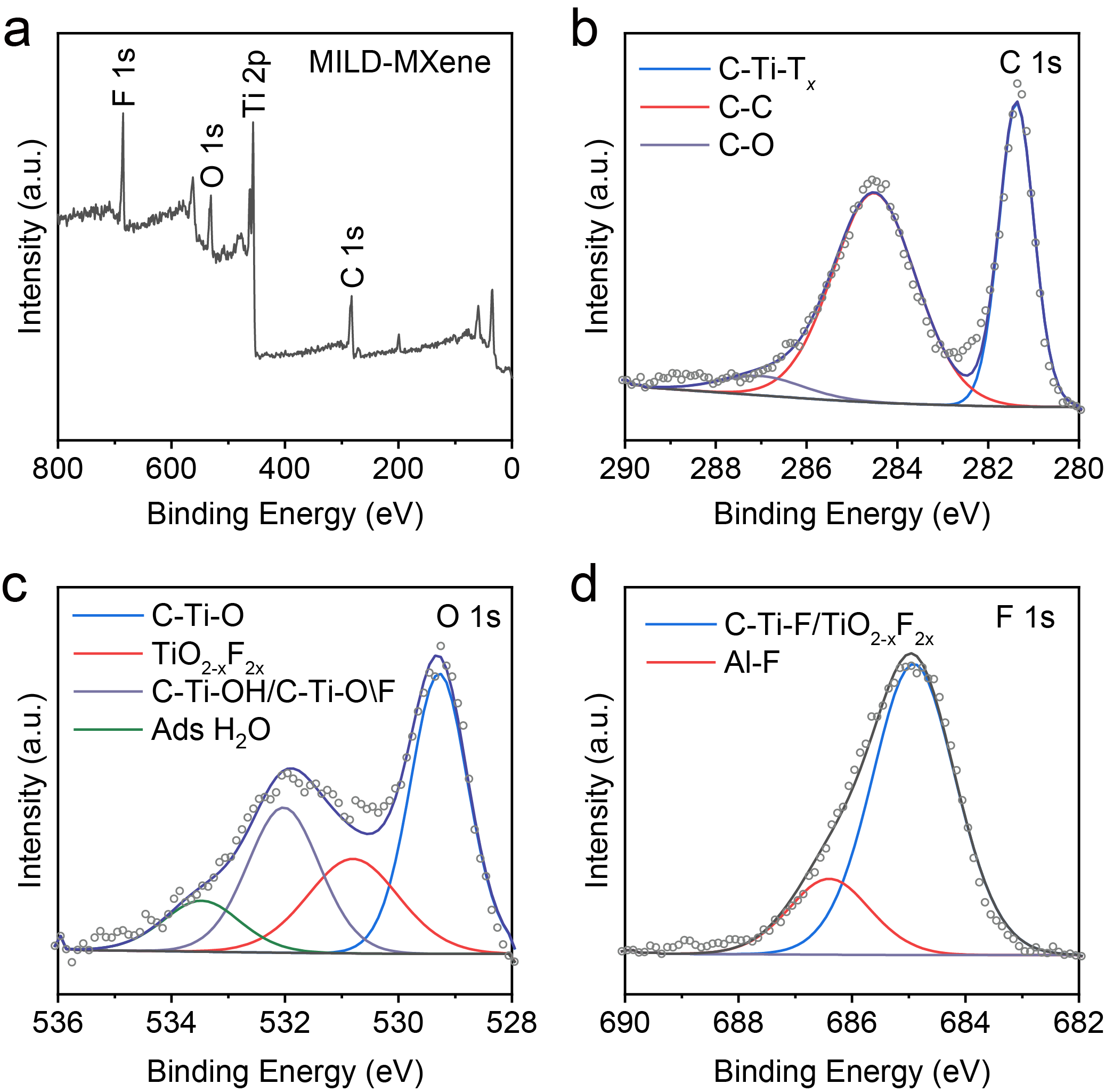


**Figure S18.** XPS analysis of MILD-MXene: (a) survey spectrum and high-resolution spectra of (b) C 1s, (c) O 1s, and (d) F 1s spectra with fitting curves.


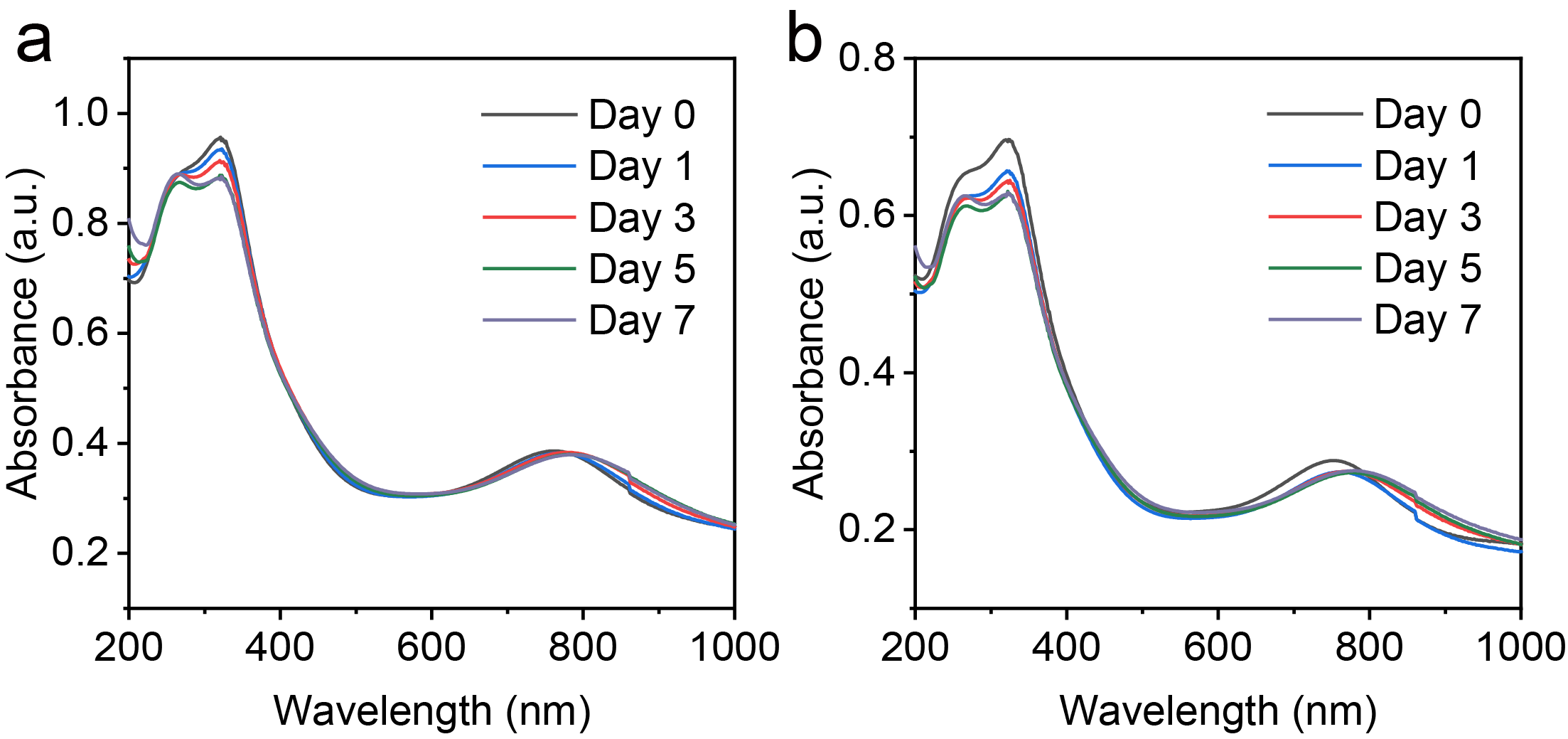


**Figure S19.** UV-Vis spectra recorded over time for (a) RISE-MXene_1_ and (b) MILD-MXene aqueous dispersions stored under ambient conditions. Both dispersions had a starting concentration of approximately 0.01 mg mL^−1^.


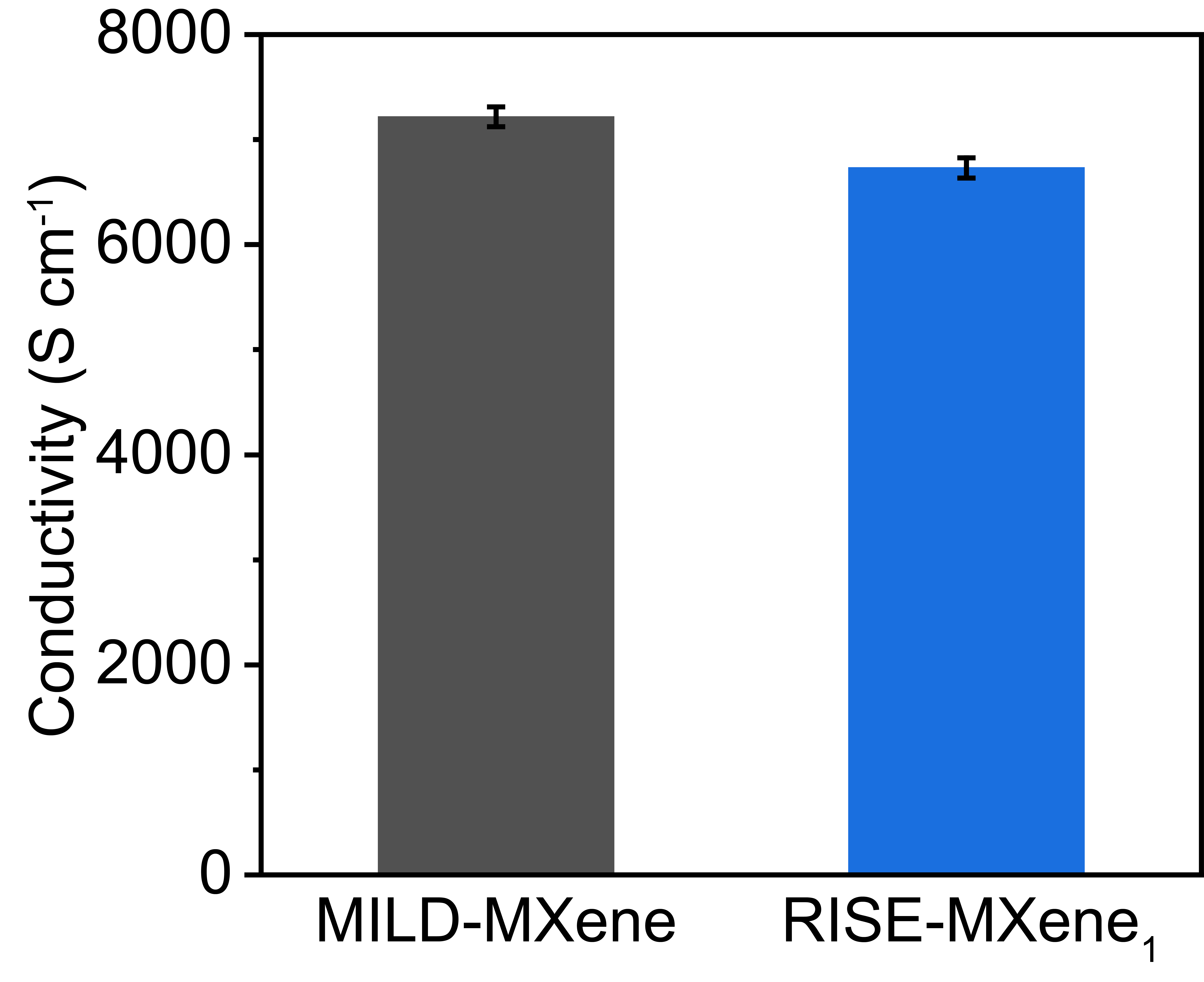


**Figure S20.** Electrical conductivity of MILD-MXene and RISE-MXene_1_ films.


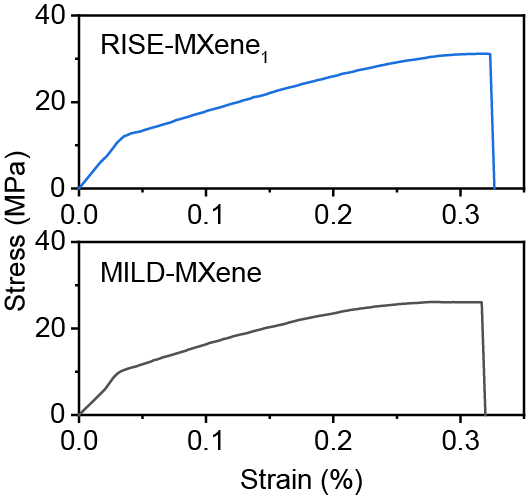


**Figure S21.** Tensile stress-strain curves of MILD-MXene and RISE-MXene_1_ films.


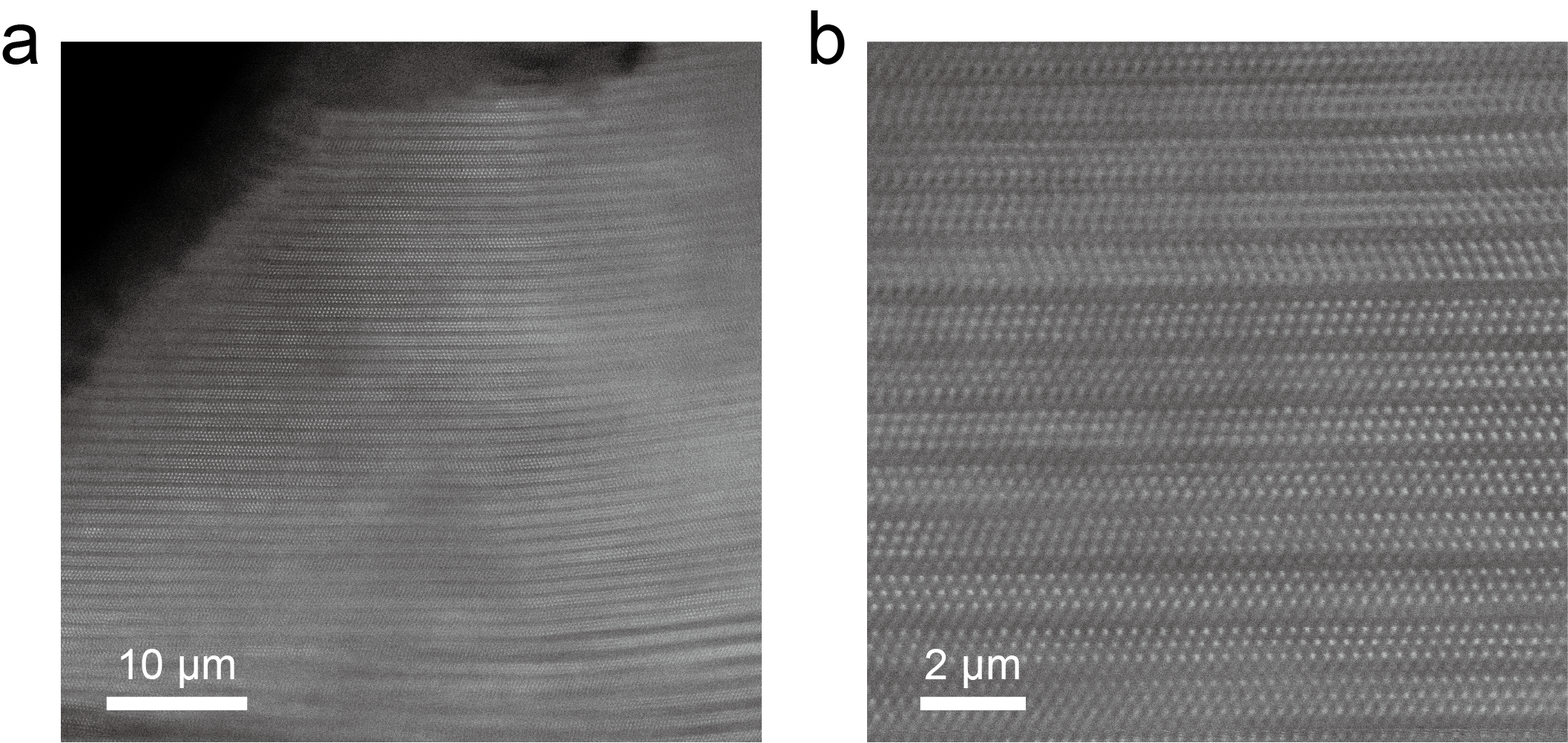


**Figure S22.** HAADF-STEM images of the RISE-MXene_1_ central region after 90 min of etching.


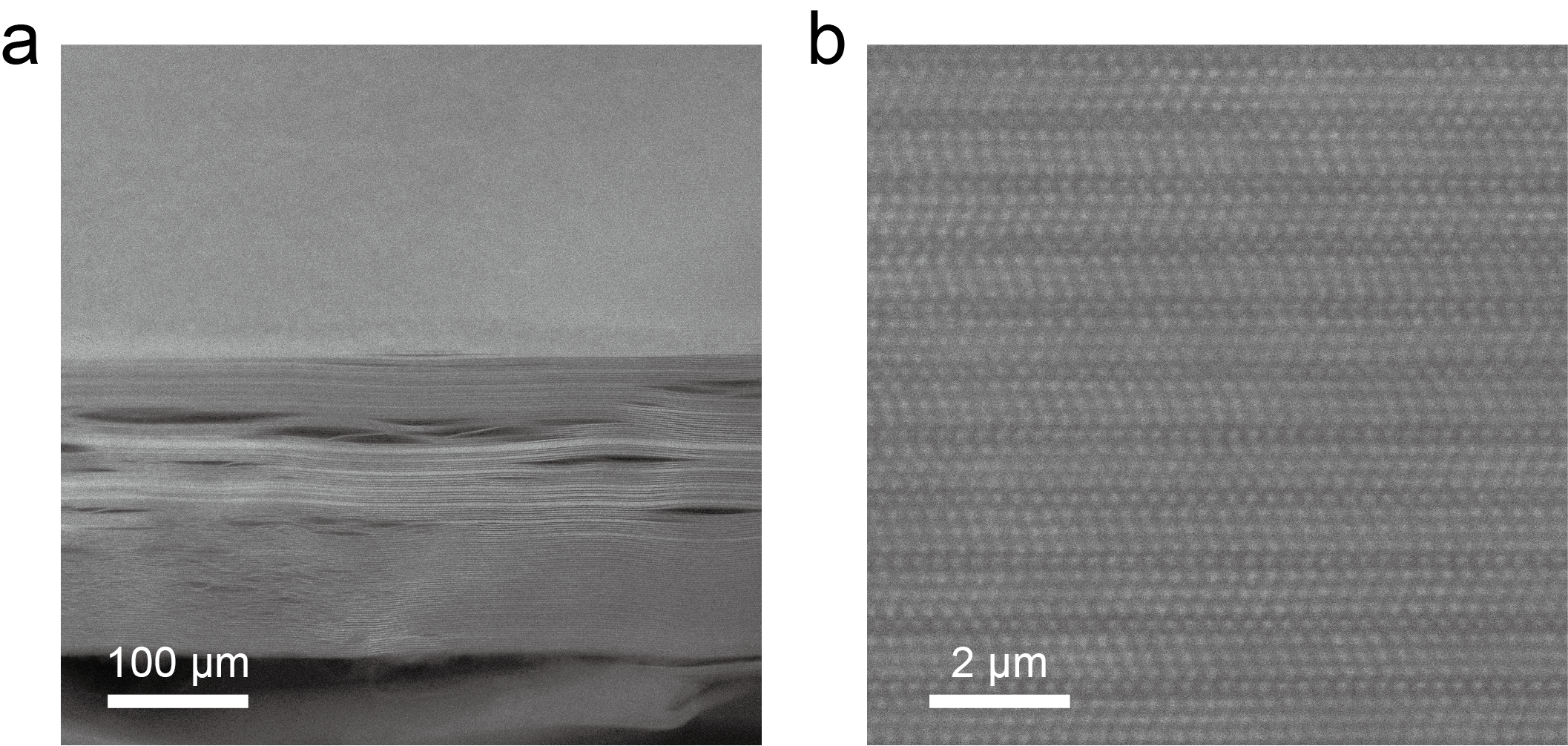


**Figure S23.** HAADF-STEM images of the MILD-MXene central region after 90 min of etching.


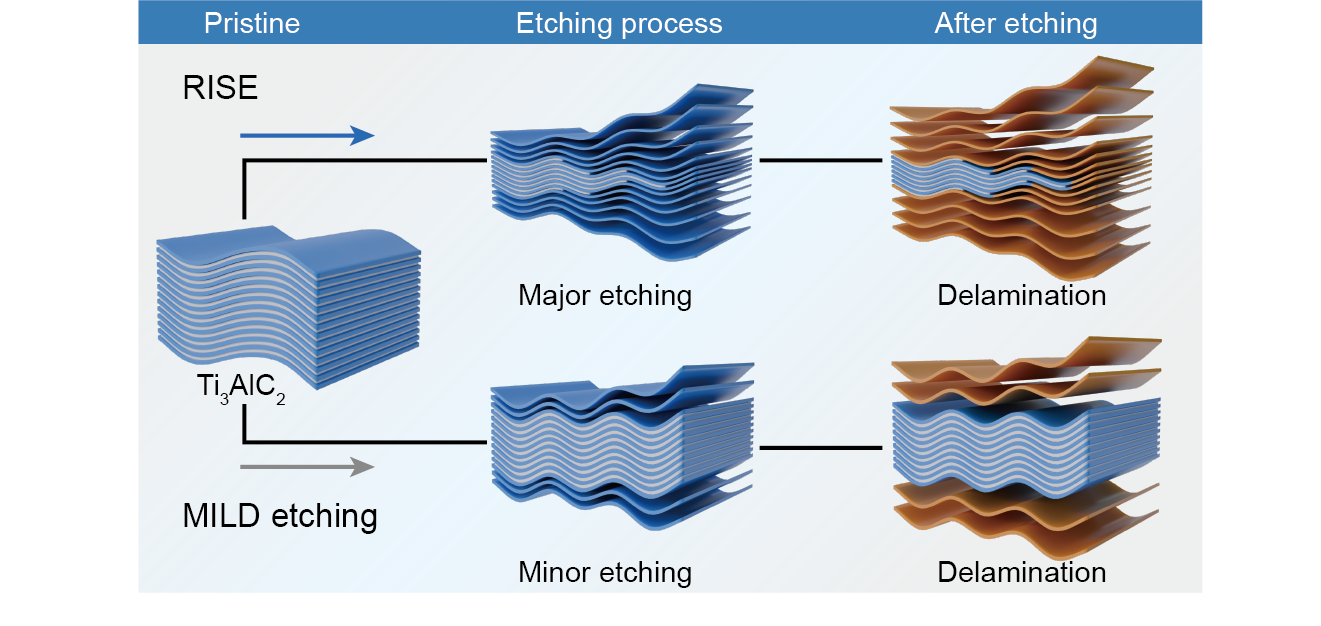


**Figure S24.** Schematic illustration of the etching process in RISE and MILD methods.


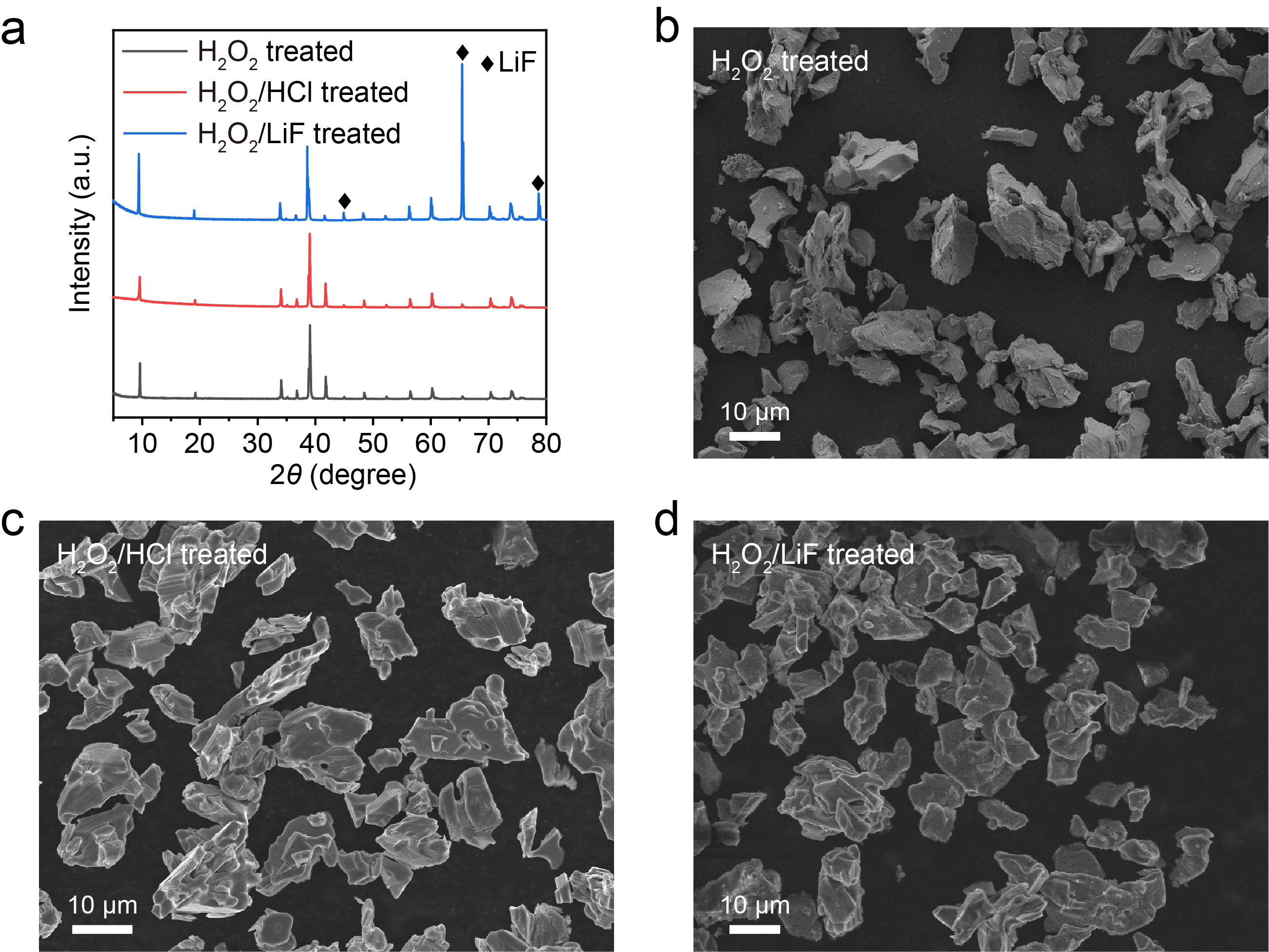


**Figure S25.** (a) XRD patterns and (b) SEM images of H_2_O_2_, H_2_O_2_/HCl, and H_2_O_2_/LiF etched MAX powders.


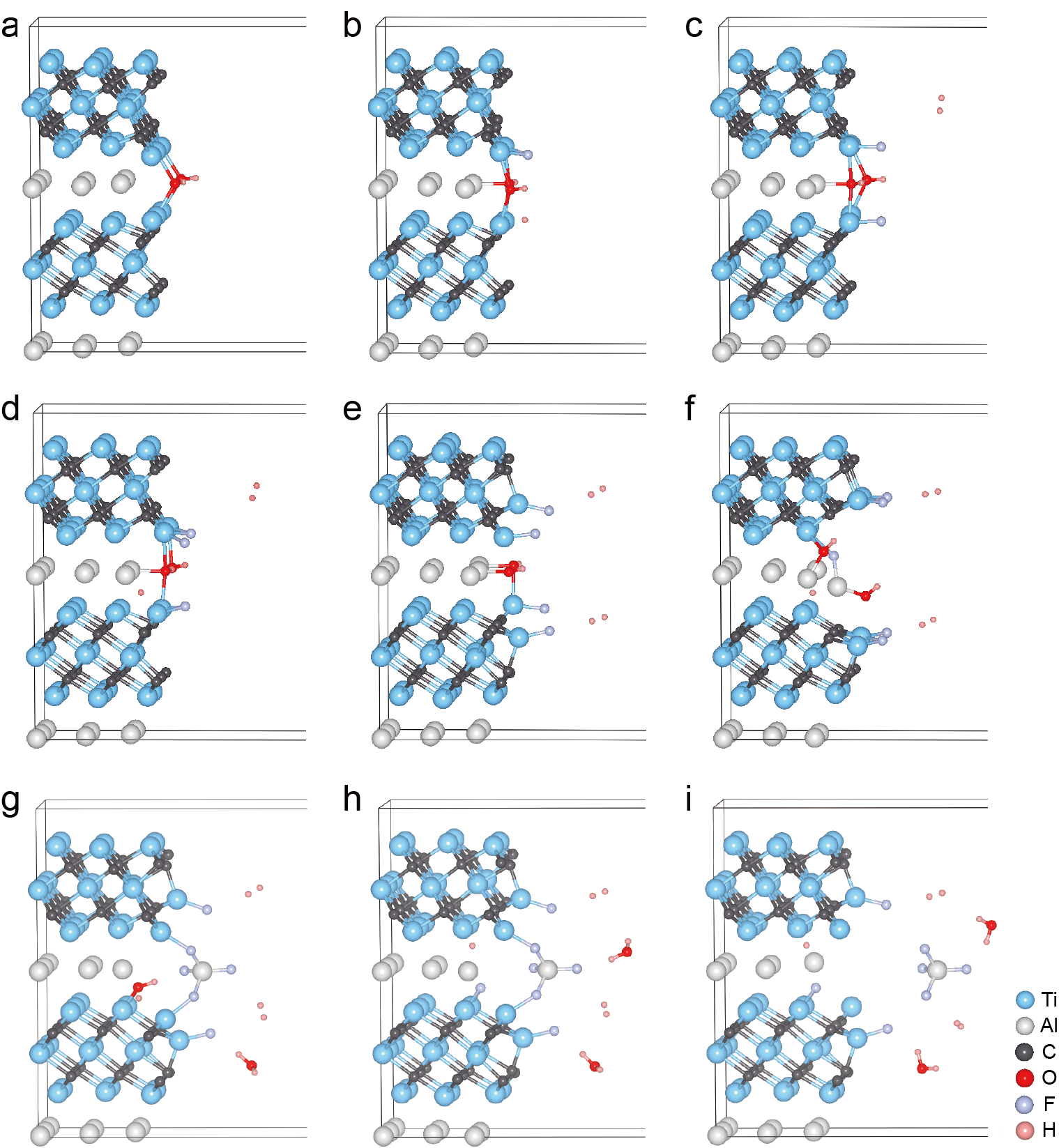


**Figure S26.** Lowest-energy atomic configurations during the RISE process: (a) pristine, (b-h) sequential HF intercalation steps, and (i) extraction of AlF^4−^.


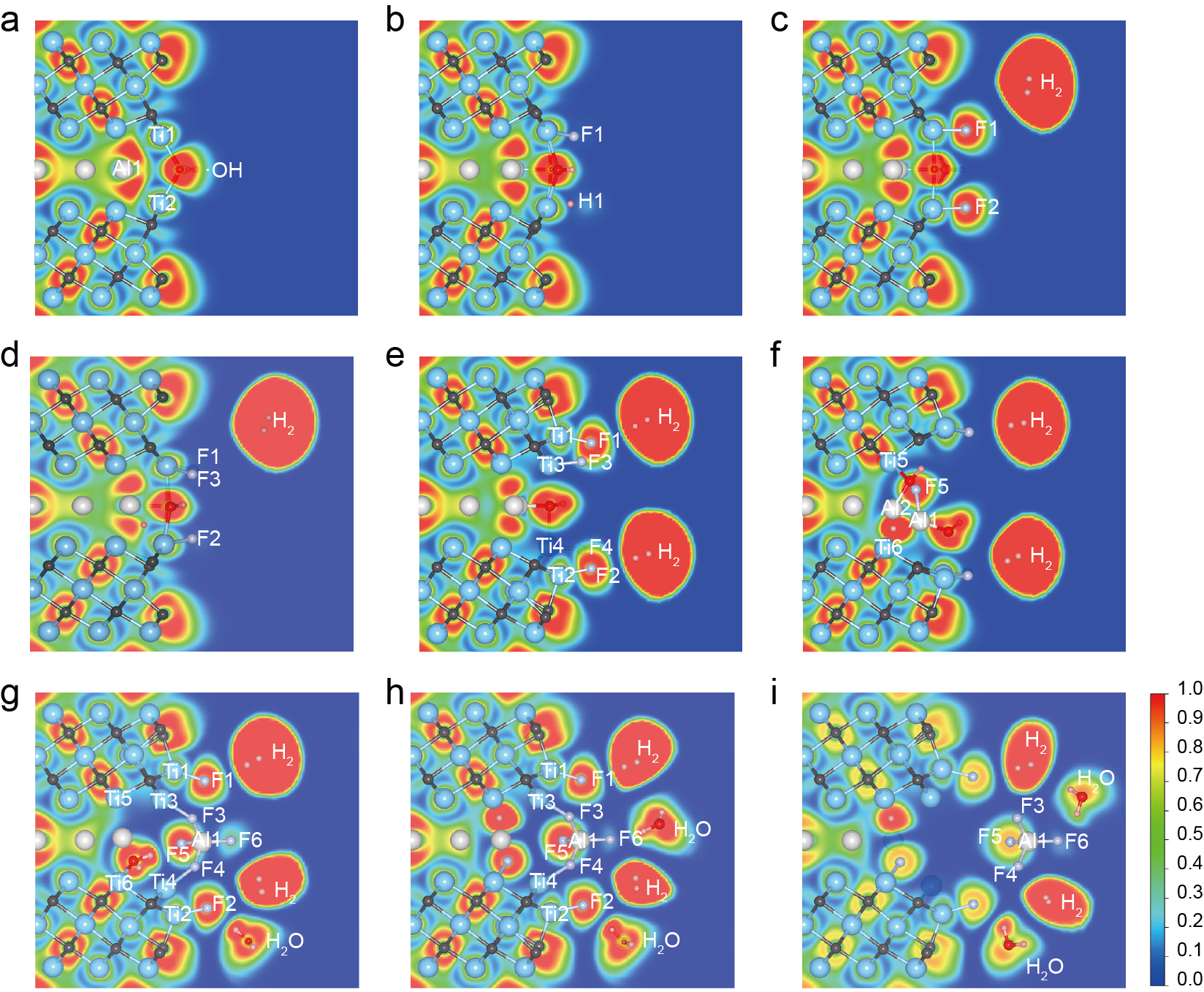


**Figure S27.** Electron localization function (ELF) plots for the RISE process at different stages.


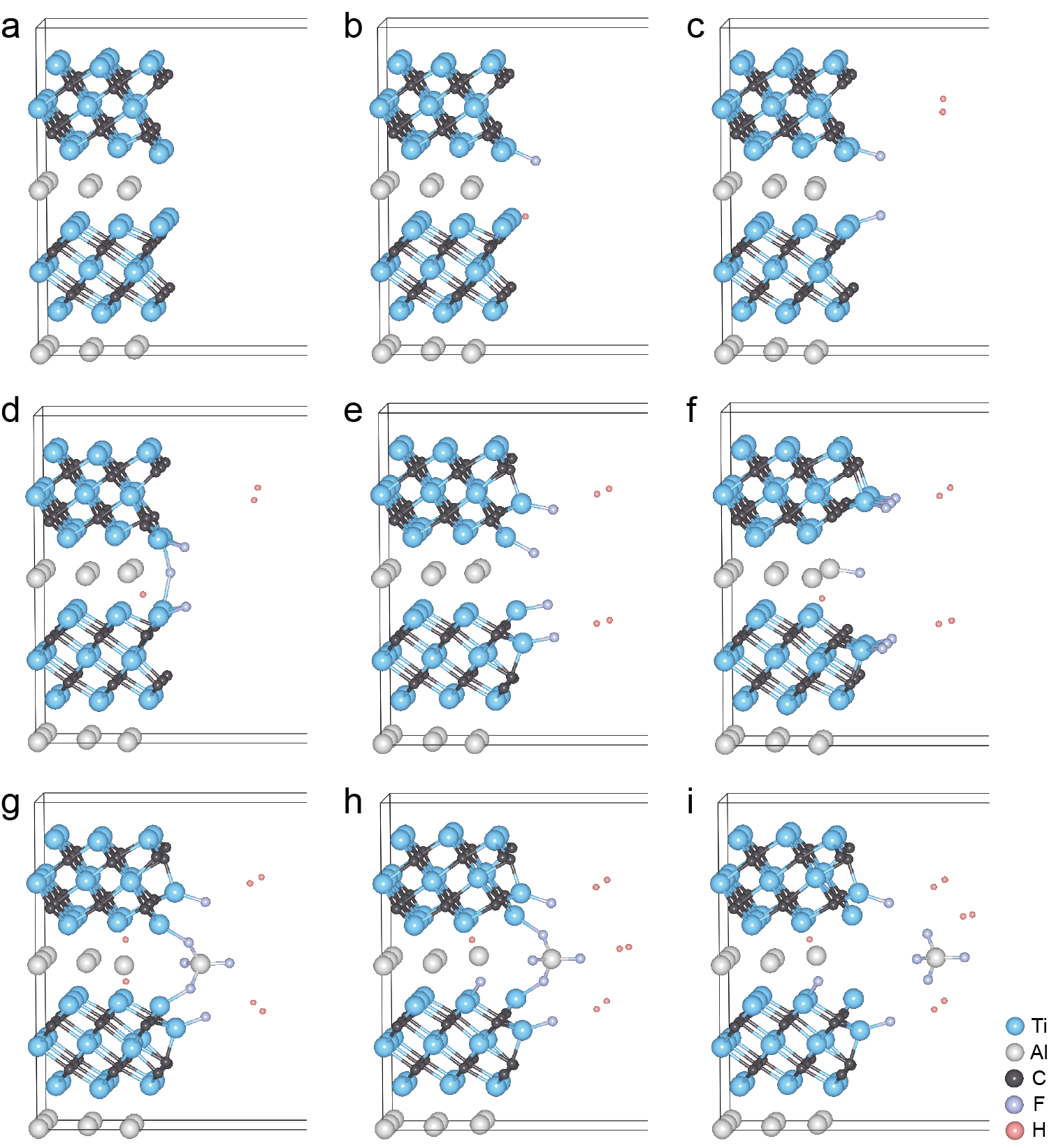


**Figure S28.** Lowest-energy atomic configurations during the conventional MILD process: (a) pristine, (b-h) sequential HF intercalation steps, and (i) extraction of AlF^4−^.


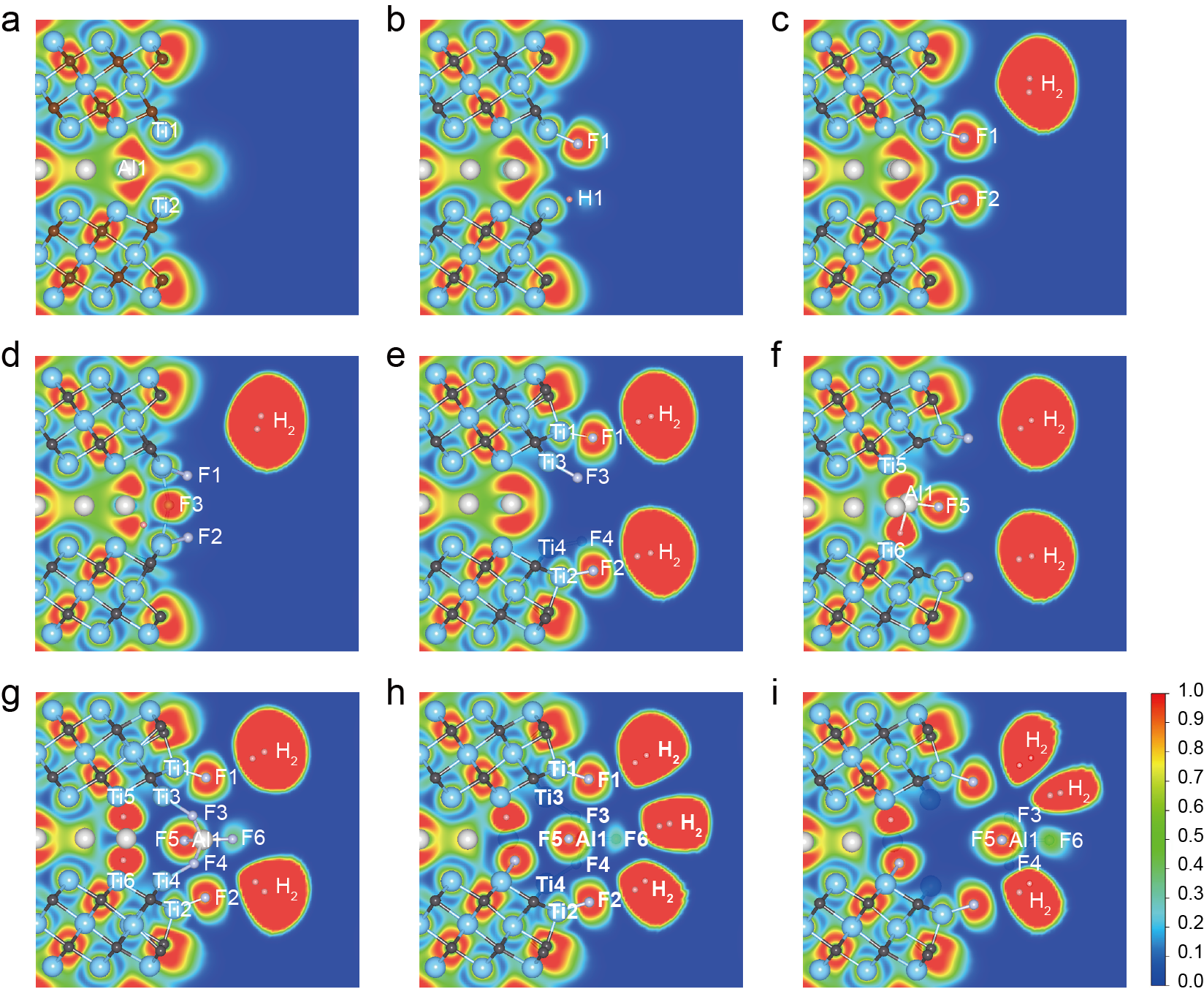


**Figure S29.** ELF plots for the conventional MILD etching process at different stages.


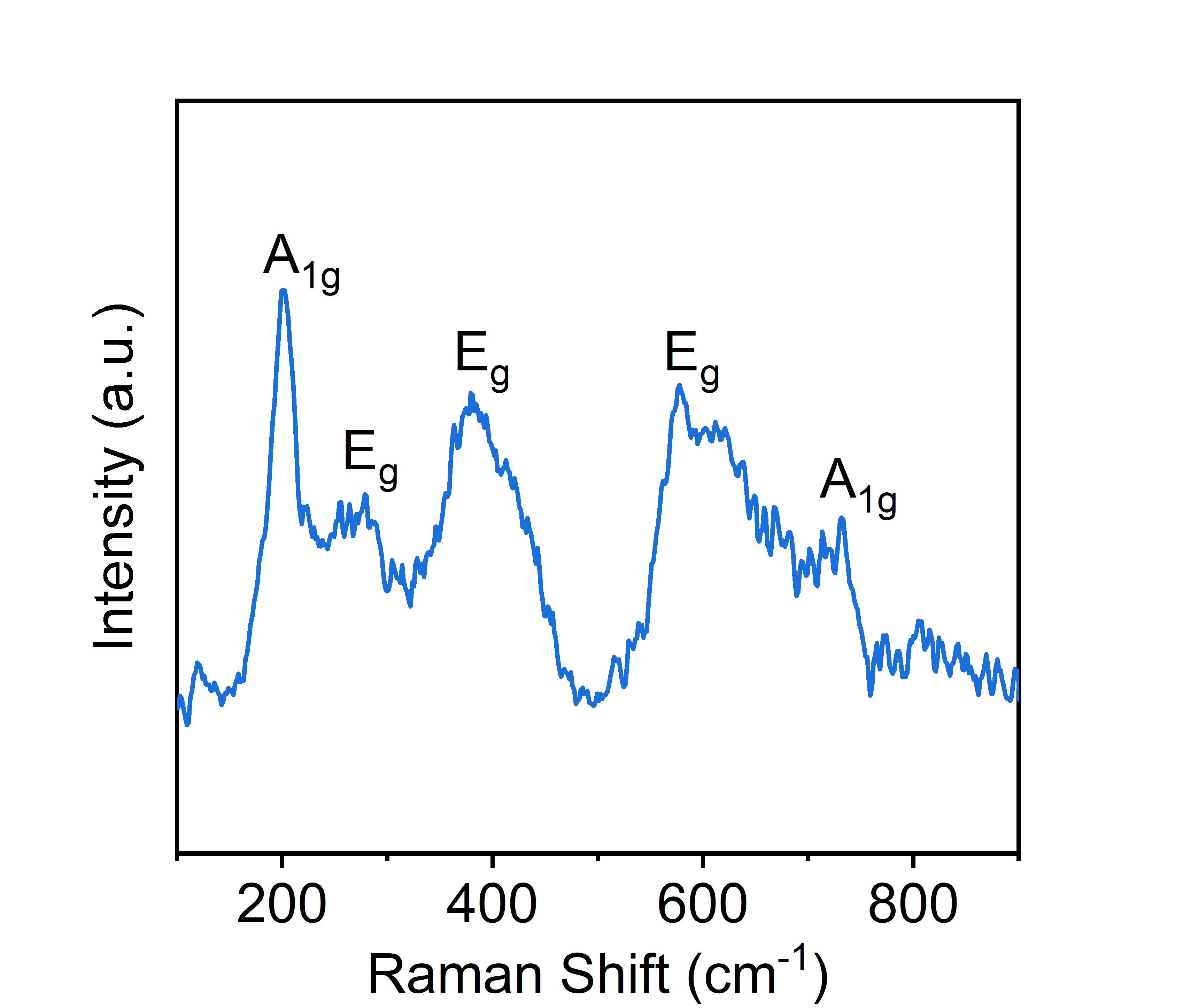


**Figure S30.** Raman spectrum of H-MXene.


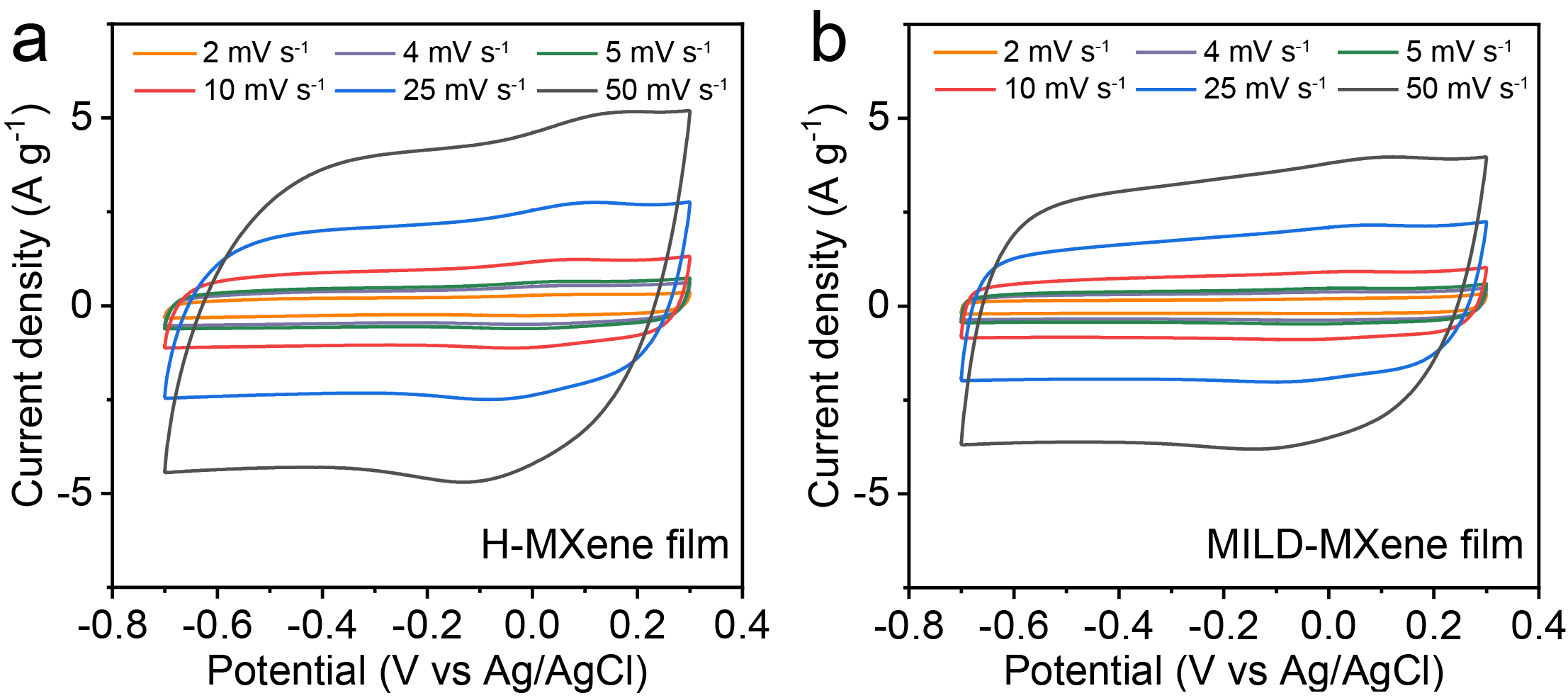


**Figure S31.** CV curves of (a) H-MXene and (b) MILD-MXene films at scan rates from 2 to 50 mV s^−1^.


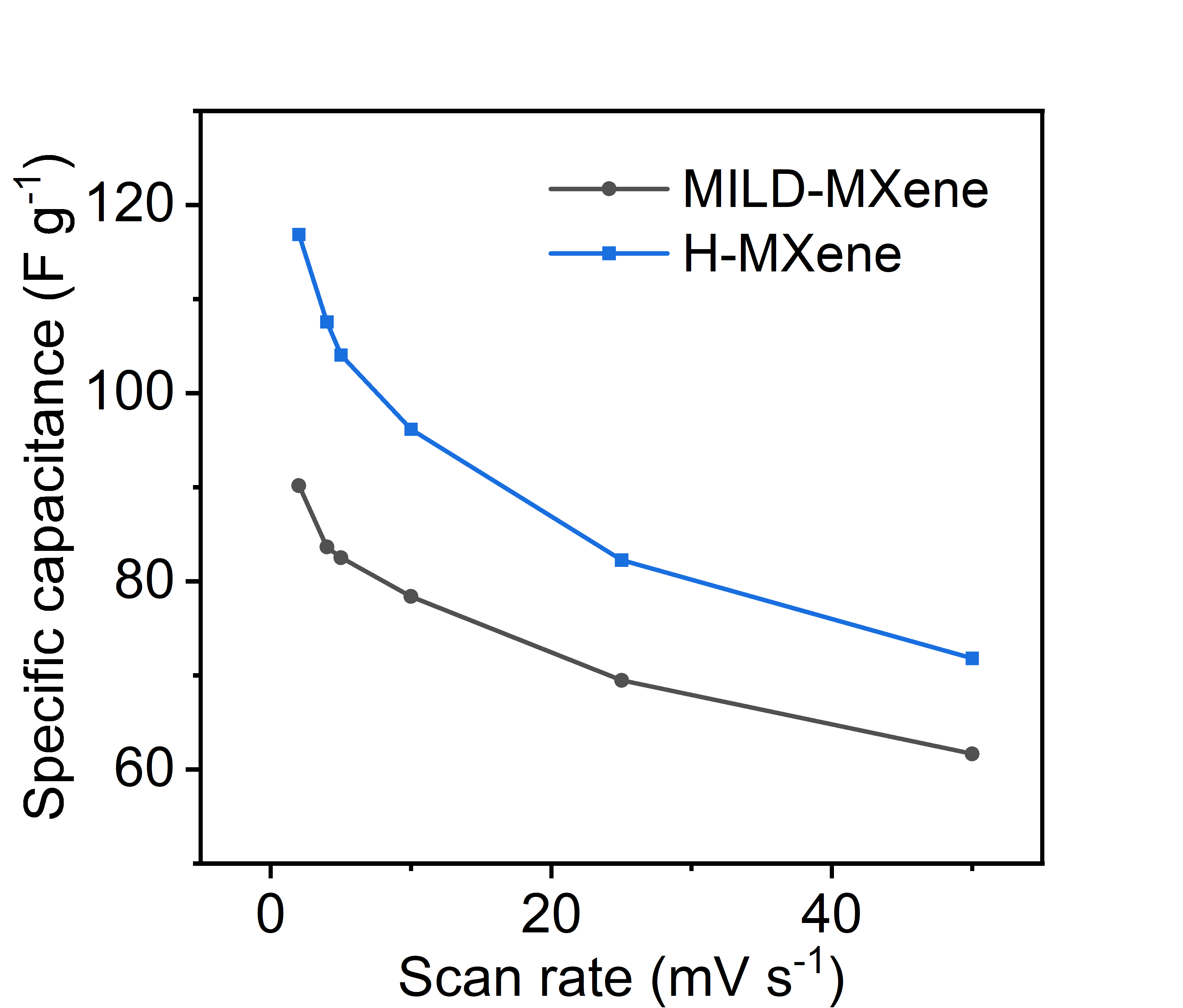


**Figure S32.** The specific capacitance of H-MXene and MILD-MXene films at various scan rates ranging from 2 to 50 mV s^−1^.


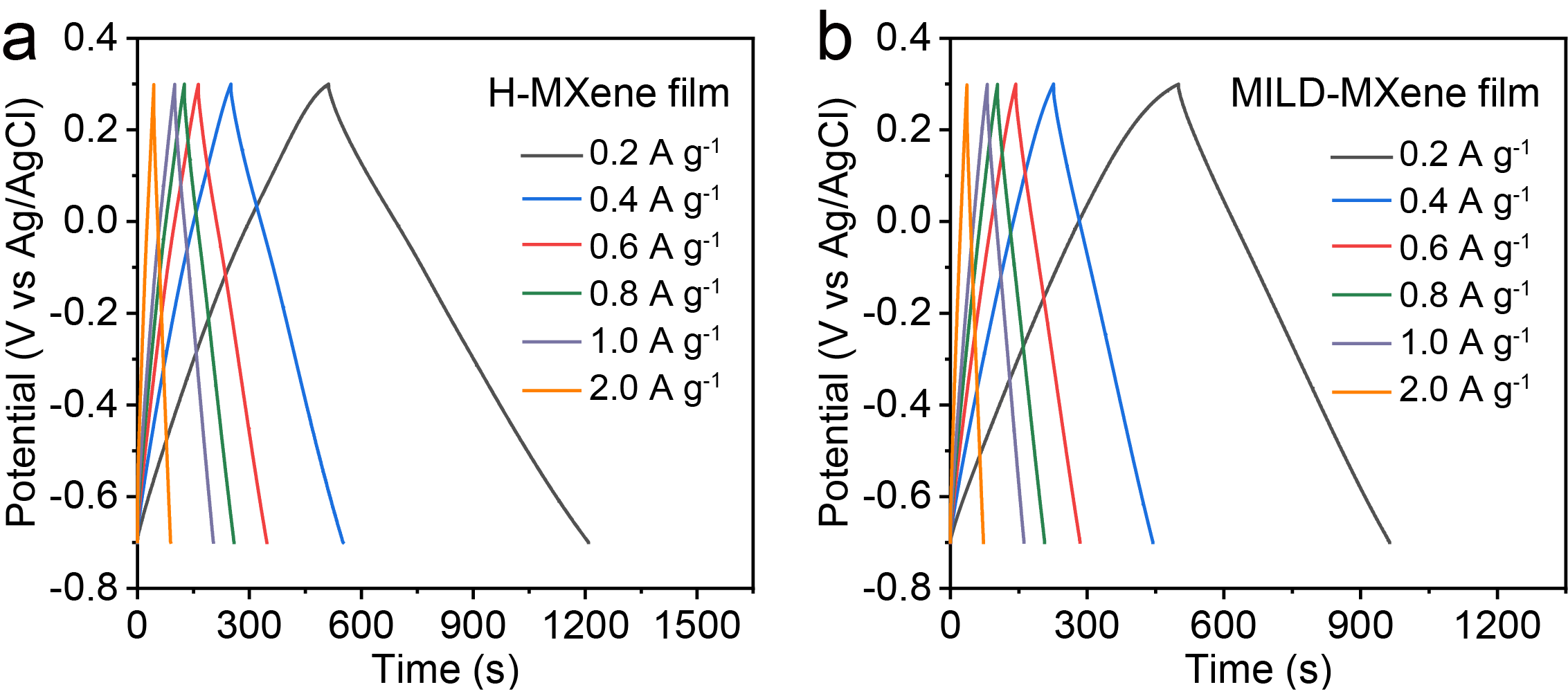


**Figure S33.** Galvanostatic charge/discharge (GCD) profiles of (a) H-MXene and (b) MILD-MXene films at current densities from 0.2 to 2.0 A g^−1^.


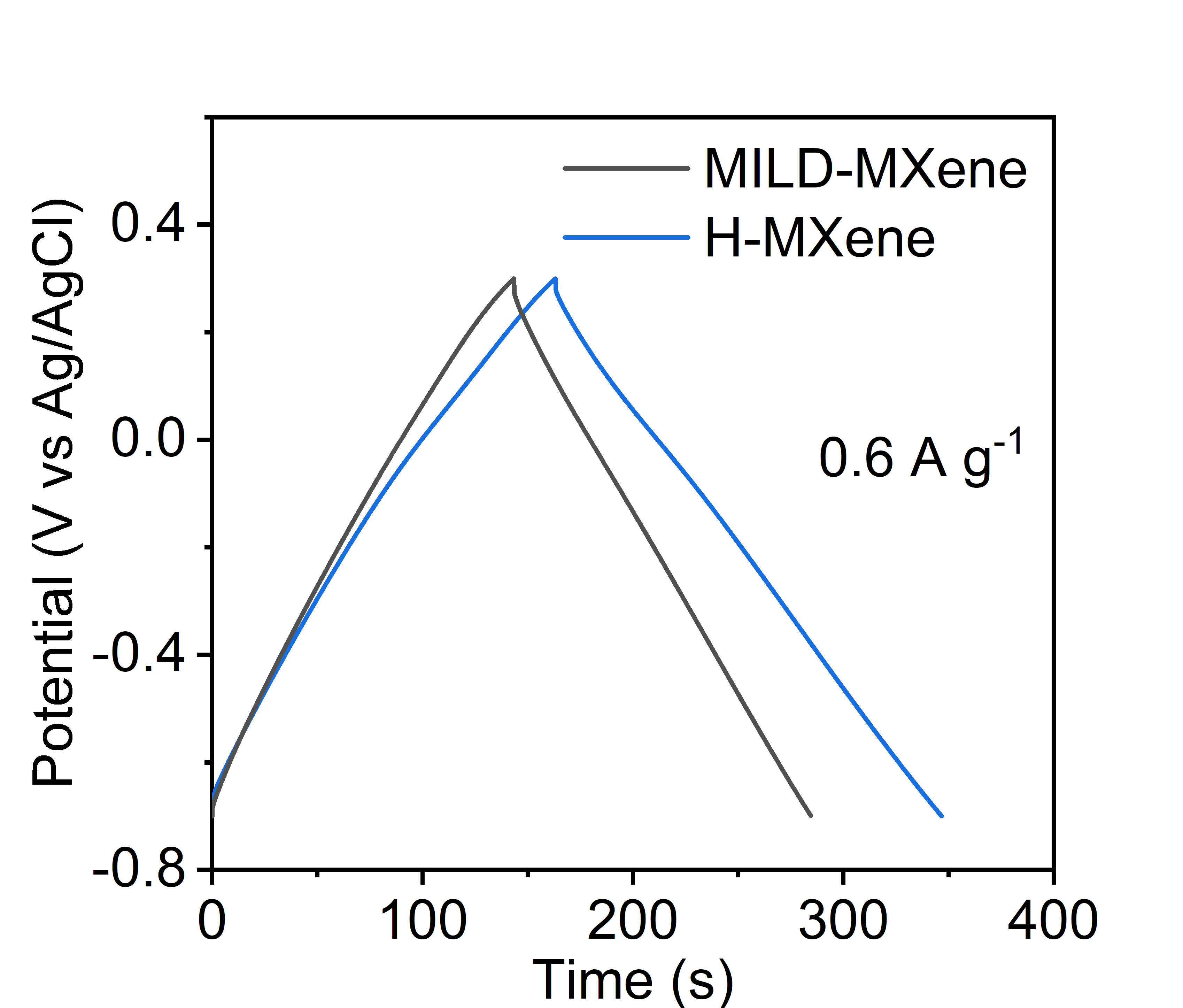


**Figure S34.** GCD profiles of H-MXene and MILD-MXene films at 0.6 A g^−1^.


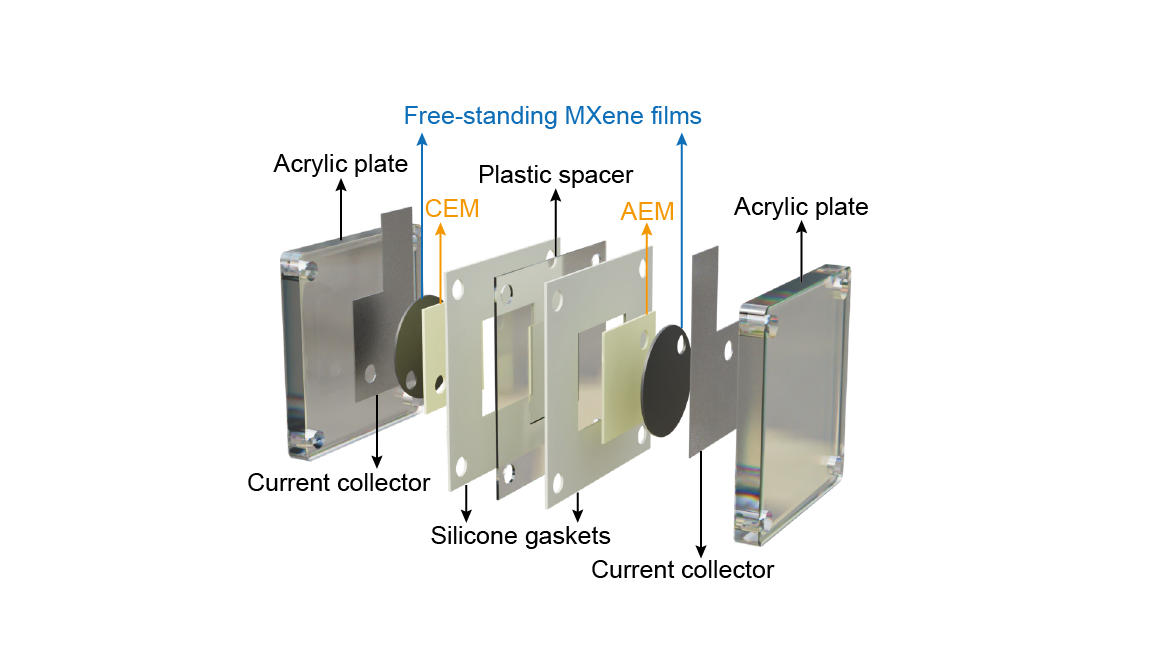


**Figure S35.** Schematic illustration of the CDI cell.


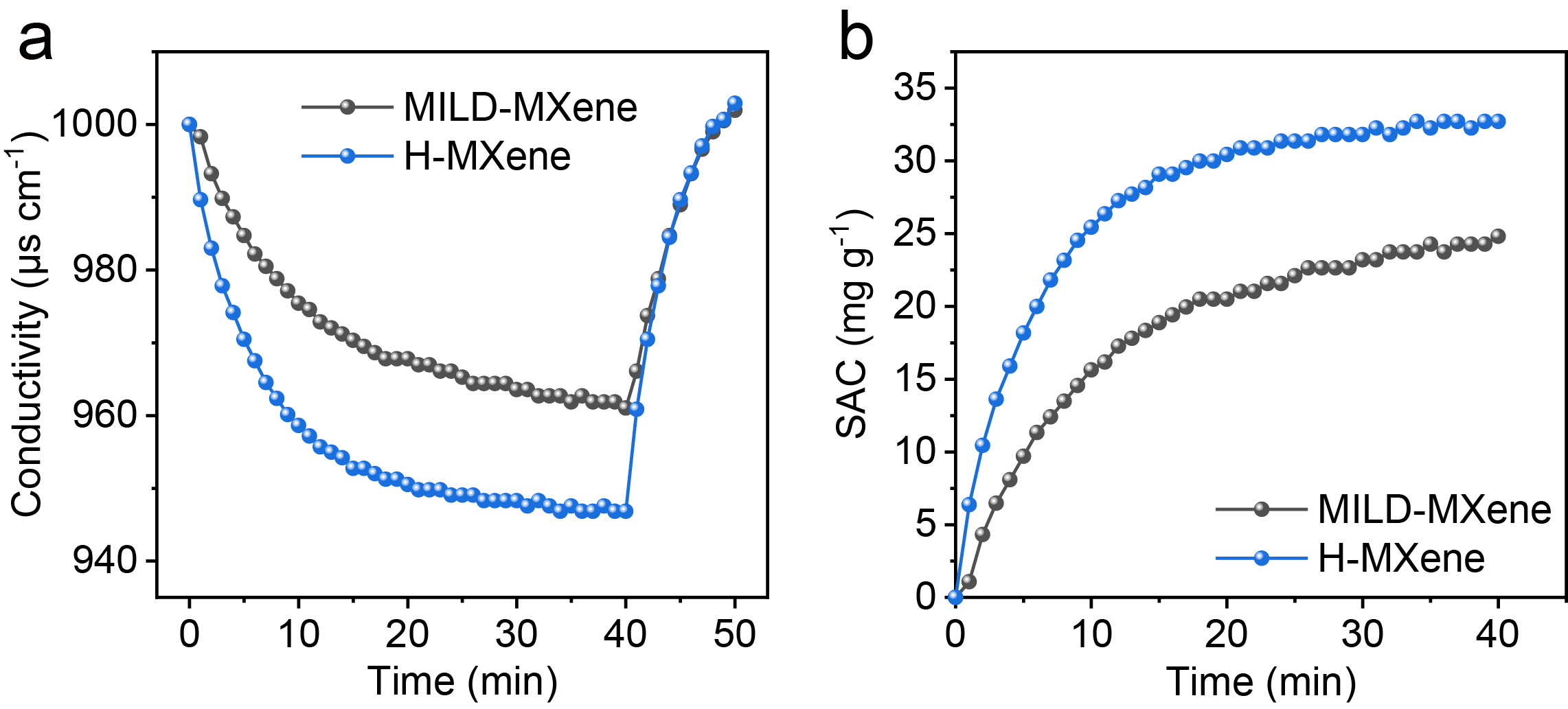


**Figure S36.** (a) Conductivity profiles and (b) deionization capacities of H-MXene and MILD-MXene films in 500 mg L^−1^ NaCl solutions at 1.2 V.


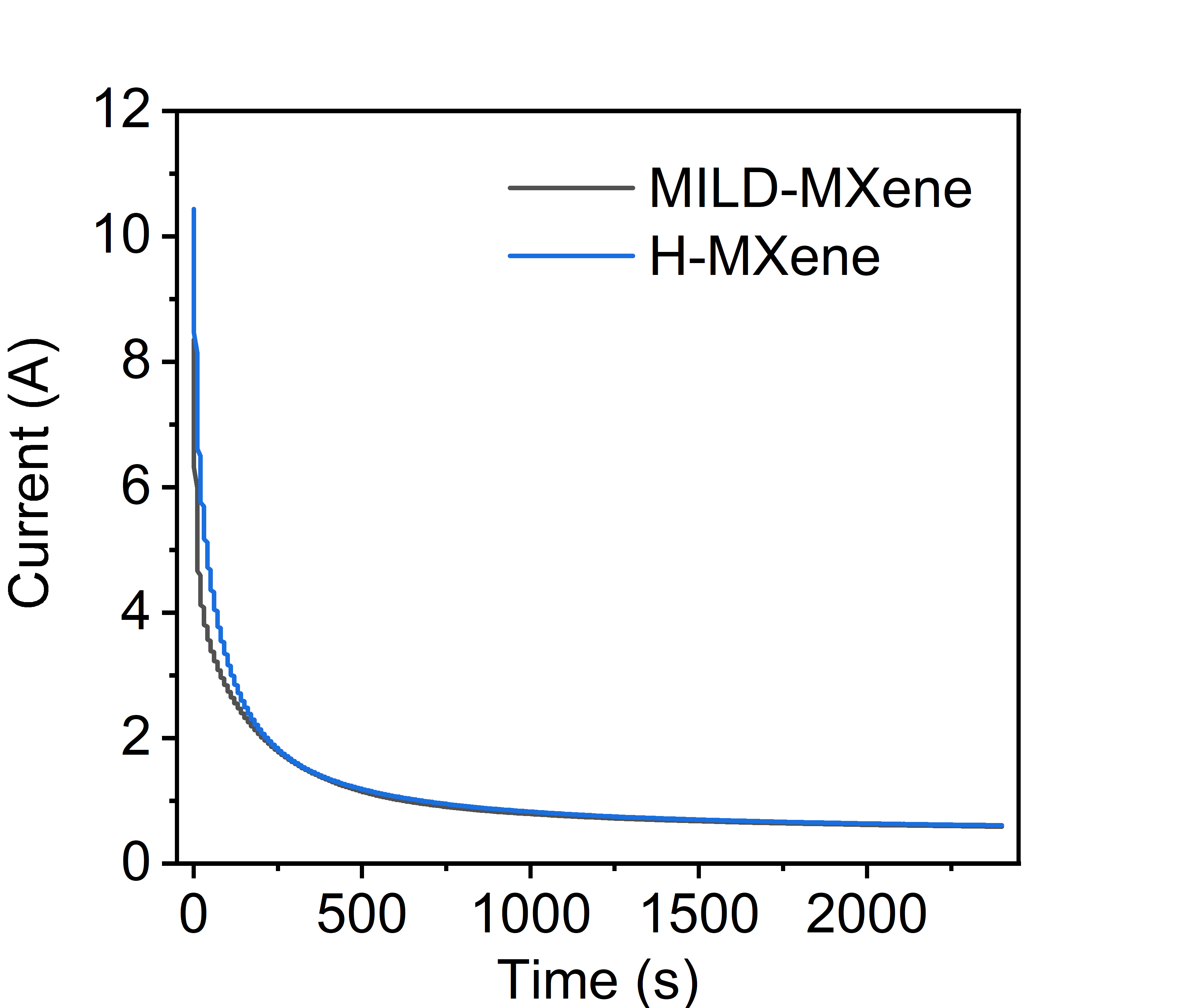


**Figure S37.** Current response curves of H-MXene and MILD-MXene films in 500 mg L^−1^ NaCl solutions at 1.2 V.


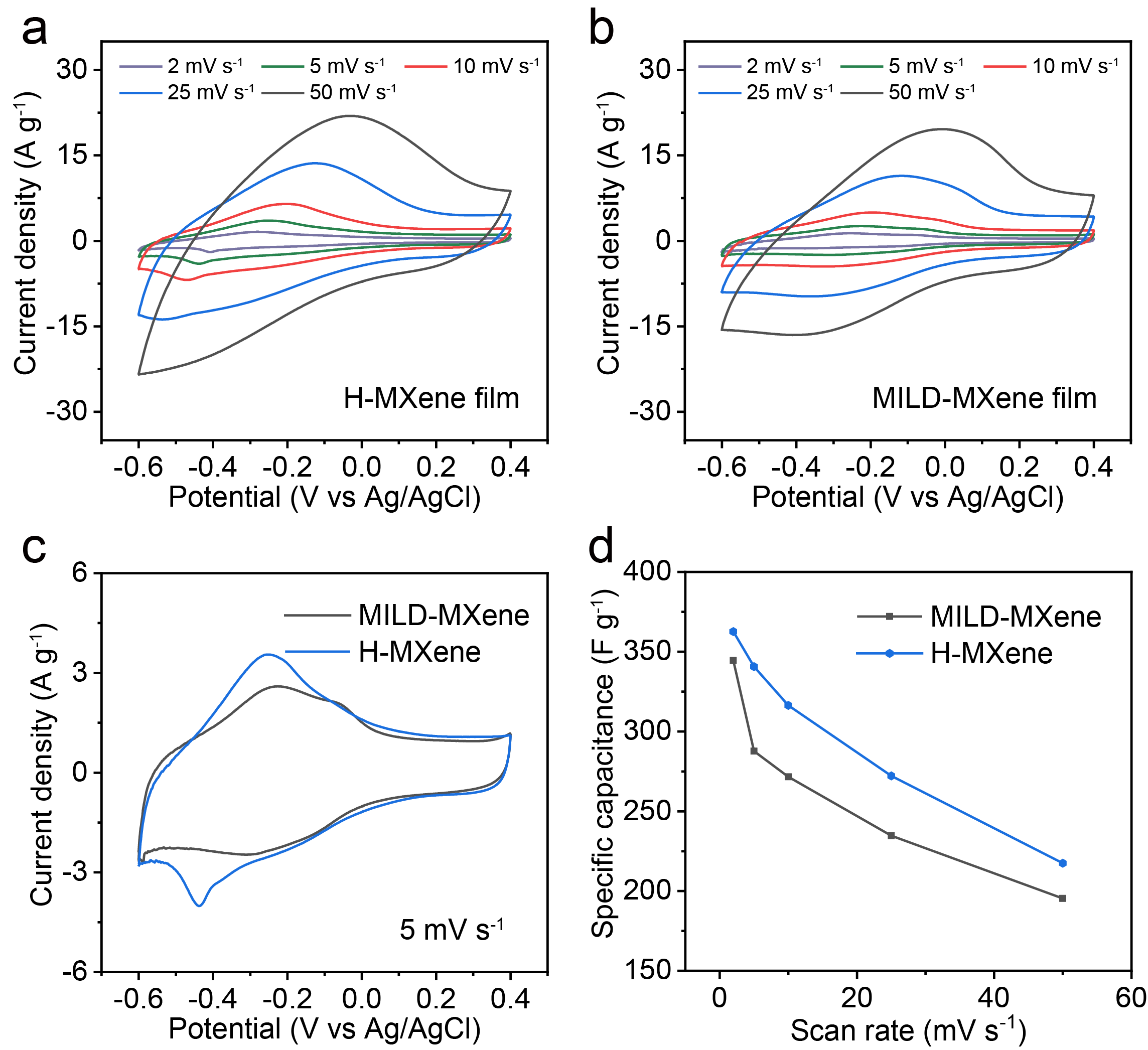


**Figure S38.** CV curves of (a) H-MXene and (b) MILD-MXene films at scan rates from 2 to 50 mV s^−1^. (c) CV curves of H-MXene and MILD-MXene at 5 mV s^−1^. (d) Specific capacitance of H-MXene and MILD-MXene at various scan rates ranging from 2 to 50 mV s^−1^.

The CV curves of both H-MXene and MILD-MXene films exhibit pronounced redox peaks, which are consistent with the pseudocapacitive energy storage mechanism inherent to MXene (Figure S38a–b). Benefiting from the increased electrochemically active sites provided by its nanohole structure, H-MXene exhibits a larger integrated CV area compared to MILD-MXene at a scan rate of 5 mV s^−1^ (Figure S38c). Furthermore, H-MXene consistently outperforms MILD-MXene in specific capacitance across all tested scan rates, achieving a high value of 362.6 F g^−1^ at 2 mV s^−1^ (Figure S38d).


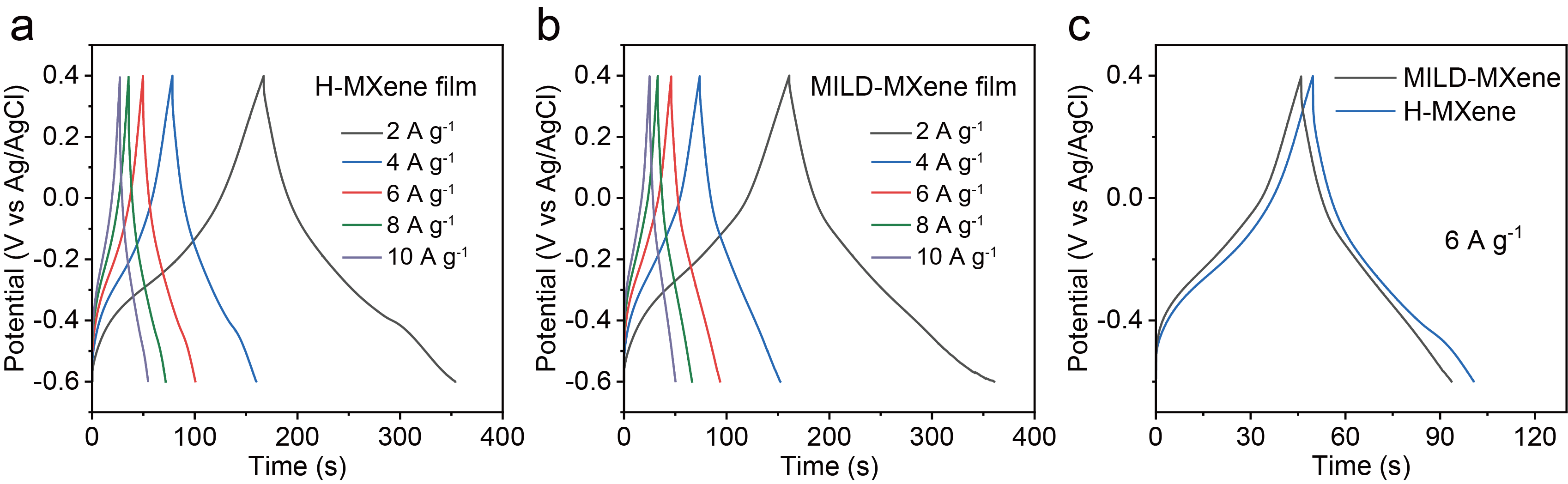


**Figure S39.** GCD profiles of (a) H-MXene and (b) MILD-MXene films at current densities from 2 to 10 A g^−1^. (c) Comparison of GCD profiles of H-MXene and MILD-MXene at 6 A g^−1^.

The GCD profiles of H-MXene and MILD-MXene films both exhibit quasi-triangular characteristics (Figure S39a–b), consistent with pseudocapacitive charge-storage behavior. Notably, H-MXene displays longer discharge times than MILD-MXene under the same current density (Figure S39c), indicating enhanced charge storage/capacitance, which is consistent with the improved ion accessibility associated with the in-plane nanohole architecture.


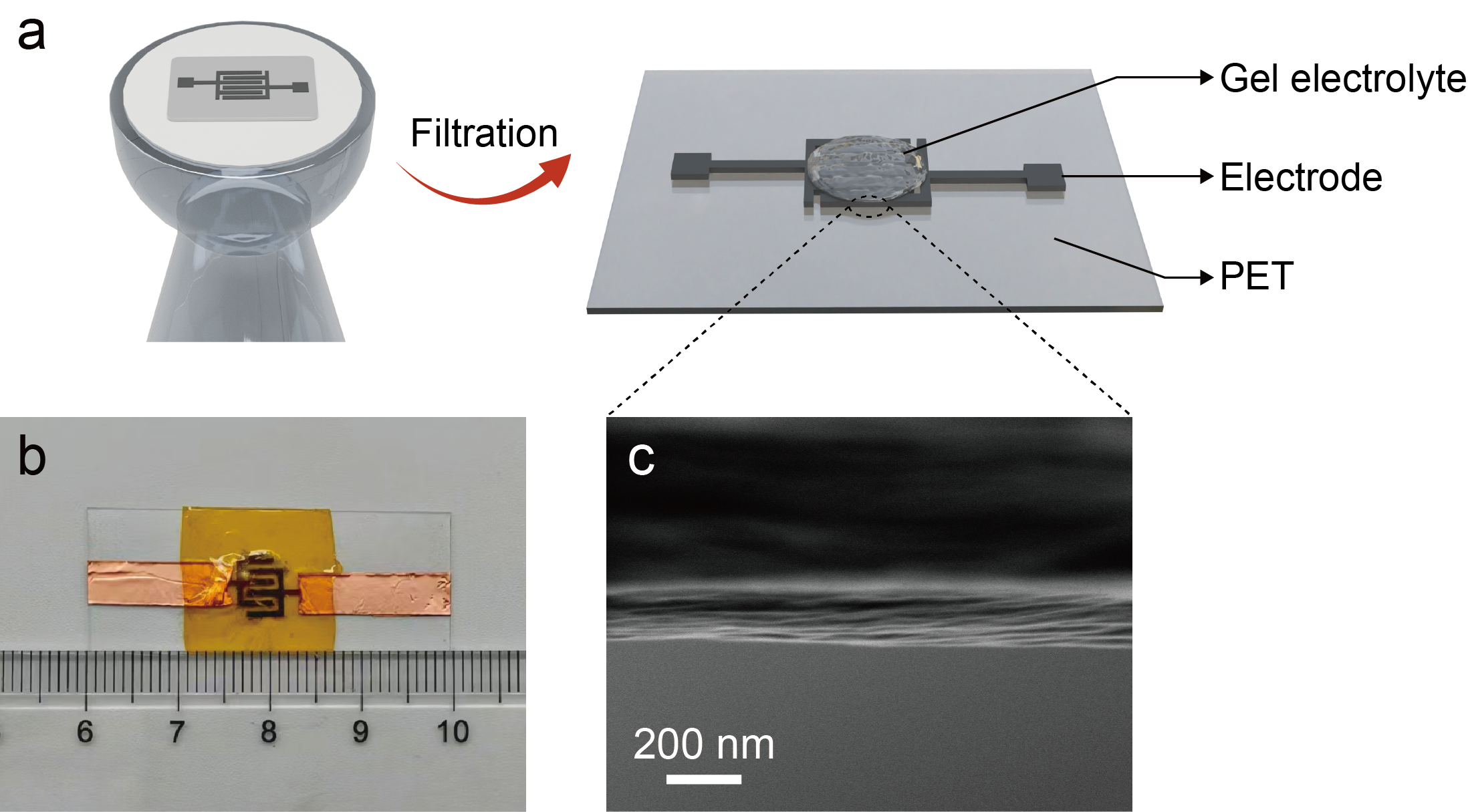


**Figure S40.** (a) Schematic illustration of the fabrication process of H-MXene MSC. (b) Optical photographs of MSC. (c) Cross-sectional SEM images of the MXene electrode.


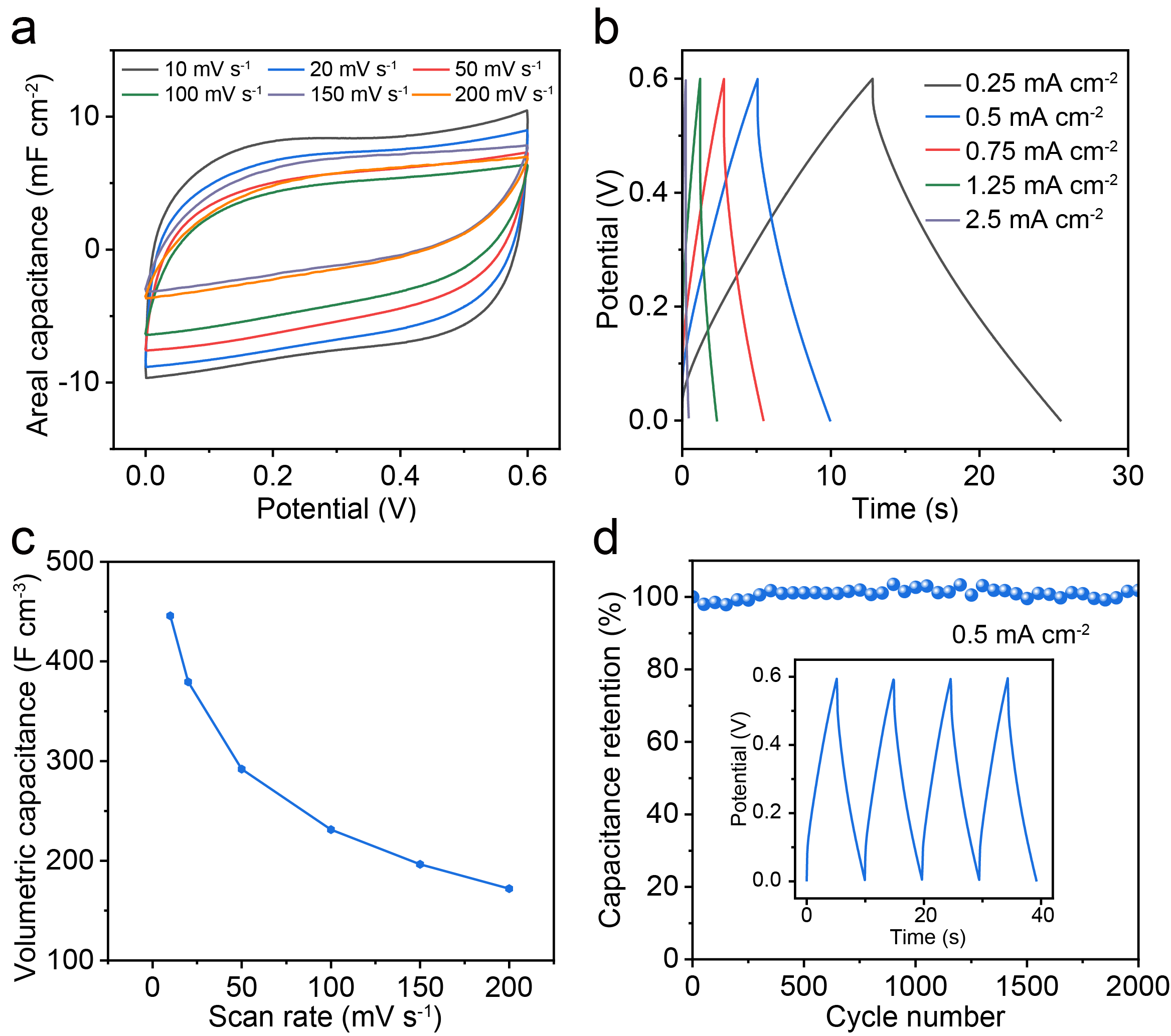


**Figure S41.** CV curves of the MSC measured at different scan rates. (b) GCD profiles of the MSC at different current densities. (c) The volumetric capacitances of the MSC at various scan rates ranging from 10 to 200 mV s^−1^. (d) Capacitance retention over 2000 charging/discharging cycles.

The CV curves of the H-MXene MSC exhibit quasi-rectangular profiles across various scan rates, with the characteristic profile well maintained even at high scan rates (Figure S41a). Consistently, the GCD profiles display nearly symmetric quasi-triangular profiles, indicative of excellent coulombic efficiency (Figure S41b). The H-MXene MSC device delivers an outstanding volumetric capacitance of 445.8 F cm^−3^ at 10 mV s^−1^, and retains 38.6% of its initial capacitance as the scan rate increases to 200 mV s^−1^ (Figure S41c). Moreover, the H-MXene MSC demonstrates good cycling stability, retaining near-initial performance after 2000 charge-discharge cycles (Figure S41d).

**Table S1.** Comparison of different synthetic strategies of MXene nanosheets (etching by HF or *in-situ* generated HF).

| Methods | Later size  (μm) | Relative etching efficiency  (% h^-1^) | Yield  (%) | Ref. |
| --- | --- | --- | --- | --- |
| Organic solvent-assisted intercalation and collection | 3.02 | 1.68 | 40.2 | [6] |
| Power-focused delamination | 4.9 | 2.55 | 61.2 | [7] |
| Hydrothermal assisted intercalation | 0.3~1 | 3.08 | 74.0 | [8] |
| Freeze-and-thaw-assisted approach | 5.52  0.878 | 1.63  3.39 | 39.0  81.4 | [9] |
| Scalable synthesis | 2.5 | 2.17 | 52.0 | [10] |
| Evaporated-Nitrogen MILD | 1.8 | 2.50 | 60.0 | [11] |
| Edge-anchored CNT-assisted exfoliation | 10.2  7.9 | 2.10  2.79 | 50.4  67.0 | [12] |
| Rigid biologic fibrils assisted exfoliation | 4~6 | 2.67 | 64.0 | [13] |
| Tuned microenvironment method | 2.885 | 2.66 | 63.9 | [14] |
| RISE | 4.1 | 27.2 | 81.6 | This work |

**Table S2.** The element compositions of RISE-MXene and MILD-MXene by XPS analysis.

| Films | Atomic percentage (%) | | | |
| --- | --- | --- | --- | --- |
|  | Ti | O | C | F |
| RISE-MXene_0_ | 17.81 | 16.88 | 42.92 | 22.39 |
| RISE-MXene_0.5_ | 22.77 | 14.97 | 46.85 | 15.41 |
| RISE-MXene_1_ | 21.70 | 15.97 | 50.34 | 11.99 |
| RISE-MXene_1.5_ | 23.38 | 13.09 | 50.17 | 13.36 |
| MILD-MXene | 19.12 | 14.26 | 45.85 | 20.77 |

**Table S3.** EDS data for the different etching processes of RISE-MXene_1_ and MILD-MXene.

| Etching time | Powders | Element weight (%) | | | | |
| --- | --- | --- | --- | --- | --- | --- |
|  |  | Ti | Al | C | O | F |
| 0 min | MAX | 82.06 | 10.86 | 6.72 | 0.34 | 0.02 |
| 30 min | MILD-MXene | 84.52 | 10.00 | 5.24 | 0.13 | 0.11 |
|  | RISE-MXene_1_ | 84.93 | 7.07 | 5.95 | 0.78 | 1.27 |
| 90 min | MILD-MXene | 83.97 | 9.09 | 6.19 | 0.51 | 0.24 |
|  | RISE-MXene_1_ | 86.4 | 3.94 | 6.25 | 1.38 | 2.03 |
| 150 min | MILD-MXene | 82.85 | 8.44 | 7.71 | 0.59 | 0.41 |
|  | RISE-MXene_1_ | 89.53 | 1.10 | 4.81 | 2.02 | 2.54 |
| 180 min | MILD-MXene | 85.72 | 7.54 | 5.19 | 0.66 | 0.89 |
|  | RISE-MXene_1_ | 89.28 | 0.20 | 5.73 | 2.08 | 2.71 |

Supplementary references

[1] X. Shi, Z. Yu, Z. Liu, N. Cao, L. Zhu, Y. Liu, K. Zhao, T. Shi, L. Yin, Z. Fan, “Scalable, High-Yield Monolayer MXene Preparation from Multilayer MXene for Many Applications” *Angew. Chem. Int. Ed.* **2025**, *64*, e202418420.

[2] G. Kresse, J. Furthmüller, “Efficiency of ab-initio total energy calculations for metals and semiconductors using a plane-wave basis set” *Comput. Mater.* **1996**, *6*, 15–50.

[3] P. E. Blöchl, O. Jepsen, O. K. Andersen, “Improved tetrahedron method for Brillouin-zone integrations” *Phys. Rev. B* **1994**, *49*, 16223–16233.

[4] J. P. Perdew, J. A. Chevary, S. H. Vosko, K. A. Jackson, M. R. Pederson, D. J. Singh, C. Fiolhais, “Atoms, molecules, solids, and surfaces: Applications of the generalized gradient approximation for exchange and correlation” *Phys. Rev. B* **1992**, *46*, 6671–6687.

[5] S. Grimme, J. Antony, S. Ehrlich, H. Krieg, “A consistent and accurate ab initio parametrization of density functional dispersion correction (DFT-D) for the 94 elements H-Pu” *J. Chem. Phys.* **2010**, *132*, 154104.

[6] D. Qu, Y. Jian, L. Guo, C. Su, N. Tang, X. Zhang, W. Hu, Z. Wang, Z. Zhao, P. Zhong, P. Li, T. Du, H. Haick, W. Wu, “An Organic Solvent-Assisted Intercalation and Collection (OAIC) for Ti_3_C_2_T*_x_* MXene with Controllable Sizes and Improved Yield” *Nano-Micro Lett.* **2021**, *13*, 188.

[7] Q. Zhang, R. Fan, W. Cheng, P. Ji, J. Sheng, Q. Liao, H. Lai, X. Fu, C. Zhang, H. Li, “Synthesis of Large-Area MXenes with High Yields through Power-Focused Delamination Utilizing Vortex Kinetic Energy” *Adv. Sci.* **2022**, *9*, 2202748.

[8] F. Han, S. Luo, L. Xie, J. Zhu, W. Wei, X. Chen, F. Liu, W. Chen, J. Zhao, L. Dong, K. Yu, X. Zeng, F. Rao, L. Wang, Y. Huang, “Boosting the Yield of MXene 2D Sheets via a Facile Hydrothermal-Assisted Intercalation” *ACS Appl. Mater. Interfaces* **2019**, *11*, 8443–8452.

[9] X. Huang, P. Wu, “A Facile, High-Yield, and Freeze-and-Thaw-Assisted Approach to Fabricate MXene with Plentiful Wrinkles and Its Application in On-Chip Micro-Supercapacitors” *Adv. Funct. Mater.* **2020**, *30*, 1910048.

[10] C. E. Shuck, A. Sarycheva, M. Anayee, A. Levitt, Y. Zhu, S. Uzun, V. Balitskiy, V. Zahorodna, O. Gogotsi, Y. Gogotsi, “Scalable Synthesis of Ti_3_C_2_T*_x_* MXene” *Adv. Eng. Mater.* **2020**, *22*, 1901241.

[11] A. S. Zeraati, S. A. Mirkhani, P. Sun, M. Naguib, P. V. Braun, U. Sundararaj, “Improved synthesis of Ti_3_C_2_T*_x_* MXenes resulting in exceptional electrical conductivity, high synthesis yield, and enhanced capacitance” *Nanoscale* **2021**, *13*, 3572–3580.

[12] X. Huang, J. Huang, J. Yang, D. Yang, T. Li, A. Dong, “High-Yield Exfoliation of Large MXene with Flake Sizes over 10 µm Using Edge-Anchored Carbon Nanotubes” *Adv. Funct. Mater.* **2023**, *33*, 2303003.

[13] X. Che, W. Zhang, L. Long, X. Zhang, D. Pei, M. Li, C. Li, “Mildly Peeling Off and Encapsulating Large MXene Nanosheets with Rigid Biologic Fibrils for Synchronization of Solar Evaporation and Energy Harvest” *ACS Nano* **2022**, *16*, 8881–8890.

[14] Q. Zhang, H. Lai, R. Fan, P. Ji, X. Fu, H. Li, “High Concentration of Ti_3_C_2_T*_x_* MXene in Organic Solvent” *ACS Nano* **2021**, *15*, 5249–5262.
